# Supplementary material for: Synthesis and Study of Dibenzo[b, f]oxepine Combined with Fluoroazobenzenes—New Photoswitches for Application in Biological Systems
Source: Molecules. 2022 Sep 8;27(18):5836. doi: 10.3390/molecules27185836 (PMC9504382; doi:10.3390/molecules27185836)
Supplement: Supplementary file 1 [file molecules-27-05836-s001.zip › Supporting information.pdf]

Synthesis and study of dibenzo[*b*, *f*]oxepine combined with fluoroazobenzenes - new photoswitches for application in biological systems

Filip Borys[a,b], Piotr Tobiasz[a], Jakub Sobel[a] and Hanna Krawczyk*[a]

Department of Organic Chemistry, Faculty of Chemistry, Warsaw University of Technology, Noakowskiego 3,00-664 Warsaw, Poland.

[a]Department of Organic Chemistry, Faculty of Chemistry, Warsaw University of Technology, Noakowskiego 3, 00-664 Warsaw, Poland.

hkraw@ch.pw.edu.pl or hanna.krawczyk@pw.edu.pl

[b] Laboratory of Cytoskeleton and Cilia Biology Nencki Institute of Experimental Biology of Polish Academy of Sciences, 3 Pasteur Street, 02-093Warsaw, Poland. fborys@ch.pw.edu.pl

Supporting Information

Table of Contents:

1. **Computational aspects and molecular docking** 3
2. **Experimental section**.. 85
3. **NMR spectra of obtained compounds, yield, time of reaction, melting point and high resolution mass date** 87
4. **Scope of methyl proton NMR spectrum (DMSO-D_6) of (*5h anti*) and (*5h syn*) reaction products measured at different temperatures**.. 135
5. **References**..137

1. Computational aspects and molecular docking

The optimum ground-state geometry for (**4a-4h, *E* / *Z* and 5a-5h, *E* / *Z***) compounds was calculated using the density functional theory (DFT). In calculation, the B3LYP functional and 6-31G*for basis set was employed and the continuum model (PCM; Gaussian 03W) [1,2] was used to simulate the effects of the solvent. HOMO and LUMO molecular orbitals for all compounds (**4a-4h, *E* /*Z*** and **5a-5h, *E* / *Z***) were computed. All the calculations were performed on a server equipped with a 16 quad-core XEON (R) CPU E7310 processor operating at 1.60 GHz. The operating system was Open SUSE 10.3, in DMSO as a solvent. The Avogadro program (version:1.2.0; http://avogadro.cc/) was used to obtain corresponding difference densities and molecular orbitals.

1.1 Calculated Structures

1.1.1 Optimized Geometries:

- The calculated coordinates of (**4a*E***) (the part of calculated log file) -

Standard orientation:

Center Number	Atomic Number	Atomic Type	Coordinates (Angstroms) X	Y	Z
1	6	0	1.664929	1.331860	-0.502191
2	6	0	2.008510	2.691329	-0.404910
3	6	0	3.340504	3.078516	-0.407153
4	6	0	4.387573	2.142717	-0.497953
5	6	0	4.011416	0.792232	-0.615783
6	6	0	2.685724	0.374841	-0.609635
7	6	0	5.778772	2.586703	-0.460520
8	6	0	6.853106	1.882549	-0.041442
9	6	0	6.865533	0.517745	0.488450
10	6	0	5.895252	-0.436978	0.134860
11	8	0	4.972315	-0.181488	-0.871074
12	6	0	7.860240	0.137071	1.409219
13	6	0	7.868837	-1.133717	1.977193
14	6	0	6.887426	-2.062875	1.627776
15	6	0	5.895643	-1.717749	0.706518
16	7	0	0.295874	1.005222	-0.479209
17	6	0	-0.300565	-0.219795	-0.685126
18	8	0	0.322260	-1.239974	-0.972180
19	6	0	-1.795871	-0.232817	-0.547308

20	6	0	-2.500341	-1.212623	-1.260235
21	6	0	-3.884922	-1.289720	-1.166232
22	6	0	-4.589604	-0.400445	-0.341945
23	6	0	-3.887651	0.569730	0.393753
24	6	0	-2.505518	0.649694	0.287925
25	7	0	-5.997586	-0.574611	-0.318239
26	7	0	-6.613269	0.230740	0.433396
27	6	0	-8.019752	0.066683	0.464843
28	6	0	-8.706201	0.949308	1.311383
29	6	0	-10.093913	0.877590	1.422090
30	6	0	-10.800448	-0.075638	0.685876
31	6	0	-10.116493	-0.958160	-0.161886
32	6	0	-8.733095	-0.893082	-0.276558
33	1	0	1.226838	3.443903	-0.330384
34	1	0	3.586453	4.134516	-0.328824
35	1	0	2.456712	-0.675410	-0.709819
36	1	0	5.938592	3.630072	-0.727767
37	1	0	7.807895	2.403440	0.001648
38	1	0	8.621821	0.862240	1.684507
39	1	0	8.642199	-1.405245	2.690130
40	1	0	6.873475	-3.061607	2.054309
41	1	0	-0.327537	1.797915	-0.392892
42	1	0	-1.945873	-1.904874	-1.884886
43	1	0	-4.445339	-2.035472	-1.722223
44	1	0	-4.438040	1.240614	1.043669
45	1	0	-1.977519	1.384352	0.890887
46	1	0	-8.130704	1.680638	1.871486
47	1	0	-10.622083	1.562472	2.079511
48	1	0	-11.882407	-0.134347	0.768974
49	1	0	-10.670942	-1.698047	-0.733143
50	1	0	-8.188132	-1.568337	-0.927051
51	8	0	4.911632	-2.628306	0.398519
52	6	0	5.060728	-3.272174	-0.872906
53	1	0	4.225413	-3.970718	-0.959907
54	1	0	5.019052	-2.546424	-1.691248
55	1	0	6.006428	-3.827751	-0.919347

Visualization of calculated geometry of (**4a*E***):

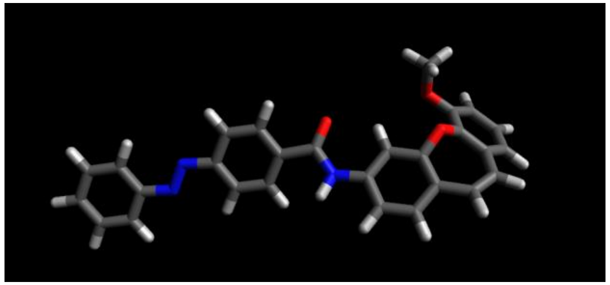

- The calculated coordinates of (**4a*Z***) (the part of calculated log file) -

Standard orientation:

Center Number	Atomic Number	Atomic Type	Coordinates (Angstroms) X	Y	Z
1	6	0	1.015002	1.079552	-0.134309
2	6	0	1.159127	2.439330	0.191474
3	6	0	2.406972	3.043185	0.141489
4	6	0	3.563718	2.330532	-0.223903
5	6	0	3.385279	0.976491	-0.563590
6	6	0	2.147890	0.344170	-0.515388
7	6	0	4.865663	2.993038	-0.239720
8	6	0	6.078862	2.421470	-0.074333
9	6	0	6.364887	1.008862	0.185159
10	6	0	5.509749	-0.017291	-0.239537
11	8	0	4.443208	0.245414	-1.090413
12	6	0	7.519851	0.655620	0.912346
13	6	0	7.783978	-0.672490	1.218764
14	6	0	6.912673	-1.687959	0.811356
15	6	0	5.764522	-1.365820	0.078009
16	7	0	-0.278354	0.529039	-0.051878
17	6	0	-0.704299	-0.732224	-0.406614
18	8	0	0.030625	-1.578334	-0.912071
19	6	0	-2.155014	-1.019867	-0.144995
20	6	0	-2.922511	-0.360808	0.828346
21	6	0	-4.258705	-0.694825	1.029205
22	6	0	-4.844229	-1.712958	0.262637
23	6	0	-4.069939	-2.413872	-0.673411
24	6	0	-2.745665	-2.054679	-0.886281
25	7	0	-6.149531	-2.232766	0.550174
26	6	0	-7.246742	-0.144207	0.303074
27	6	0	-8.009448	0.672630	1.149647
28	6	0	-8.211740	2.012063	0.824878
29	6	0	-7.708356	2.525422	-0.373693
30	6	0	-6.993747	1.695263	-1.242127
31	6	0	-6.750288	0.364710	-0.906981
32	7	0	-7.179742	-1.531857	0.658326
33	1	0	0.288022	3.022919	0.480891
34	1	0	2.498032	4.095626	0.398338
35	1	0	2.072076	-0.695990	-0.794337
36	1	0	4.831074	4.078630	-0.317739
37	1	0	6.942249	3.082888	-0.030185
38	1	0	8.195843	1.440320	1.241194
39	1	0	8.676116	-0.933463	1.781273

40	1	0	7.138974	-2.719223	1.056240
41	1	0	-0.996597	1.182907	0.230691
42	1	0	-2.480506	0.398305	1.468715
43	1	0	-4.836496	-0.188413	1.795403
44	1	0	-4.520662	-3.231765	-1.228305
45	1	0	-2.140179	-2.582738	-1.615127
46	1	0	-8.424726	0.246716	2.058726
47	1	0	-8.779856	2.649548	1.496624
48	1	0	-7.887051	3.563716	-0.638904
49	1	0	-6.622257	2.085370	-2.185784
50	1	0	-6.203725	-0.279709	-1.587497
51	8	0	4.857485	-2.269407	-0.385506
52	6	0	5.066925	-3.642461	-0.075389
53	1	0	4.228022	-4.178086	-0.522760
54	1	0	6.006123	-4.013452	-0.505110
55	1	0	5.071894	-3.813643	1.008546

Visualization of calculated geometry of (**4a*Z***):

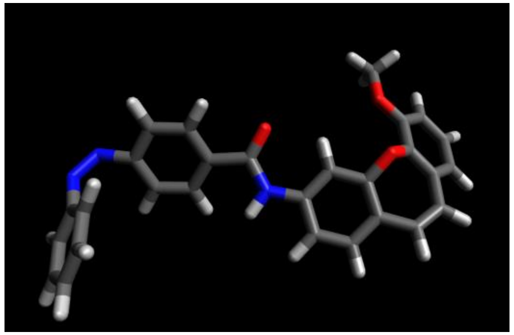

- The calculated coordinates of (**4b*E***) (the part of calculated log file) -

Standard orientation:

Center Number	Atomic Number	Atomic Type	Coordinates (Angstroms) X	Y	Z
1	6	0	-2.072991	-1.414567	-0.411623
2	6	0	-2.409635	-2.751376	-0.136256
3	6	0	-3.739375	-3.144141	-0.092769
4	6	0	-4.790524	-2.234660	-0.311507
5	6	0	-4.421816	-0.909145	-0.607044
6	6	0	-3.098120	-0.486242	-0.649689
7	6	0	-6.180556	-2.675956	-0.226226
8	6	0	-7.260827	-1.926537	0.086304
9	6	0	-7.281703	-0.504527	0.435930
10	6	0	-6.310583	0.393044	-0.029005
11	8	0	-5.388153	0.011228	-0.995309

12	6	0	-8.287560	-0.012980	1.292790
13	6	0	-8.294641	1.319338	1.683220
14	6	0	-7.307184	2.202351	1.234038
15	6	0	-6.303391	1.742980	0.373192
16	7	0	-0.704847	-1.082735	-0.428929
17	6	0	-0.111789	0.116904	-0.755418
18	8	0	-0.737252	1.103379	-1.138834
19	6	0	1.384366	0.146424	-0.624052
20	6	0	2.082067	1.068107	-1.416742
21	6	0	3.466644	1.158587	-1.335659
22	6	0	4.179226	0.340539	-0.446850
23	6	0	3.484445	-0.570142	0.367603
24	6	0	2.102213	-0.662958	0.275867
25	7	0	5.585908	0.521233	-0.443558
26	7	0	6.209479	-0.226408	0.360223
27	6	0	7.612263	-0.053549	0.373731
28	6	0	8.312130	-0.881391	1.264872
29	6	0	9.698246	-0.801547	1.367921
30	6	0	10.365451	0.117801	0.565932
31	6	0	9.698373	0.954972	-0.330336
32	6	0	8.316622	0.867493	-0.425429
33	9	0	11.707315	0.206757	0.654694
34	1	0	-1.624089	-3.482484	0.041224
35	1	0	-3.979544	-4.181975	0.124201
36	1	0	-2.876700	0.543581	-0.886196
37	1	0	-6.335013	-3.746160	-0.355061
38	1	0	-8.214653	-2.441045	0.187996
39	1	0	-9.051783	-0.695750	1.654250
40	1	0	-9.073355	1.687456	2.345436
41	1	0	-7.332946	3.239698	1.546969
42	1	0	-0.079647	-1.860639	-0.261205
43	1	0	1.521812	1.705622	-2.092367
44	1	0	4.020817	1.859960	-1.952366
45	1	0	4.040027	-1.184918	1.066713
46	1	0	1.581562	-1.348534	0.939821
47	1	0	7.748570	-1.584076	1.871137
48	1	0	10.258978	-1.431968	2.050349
49	1	0	10.268163	1.655347	-0.933162
50	1	0	7.764795	1.501828	-1.110069
51	8	0	-5.304136	2.513435	-0.139031
52	6	0	-5.253589	3.881652	0.249132
53	1	0	-4.383068	4.301208	-0.257770
54	1	0	-6.154569	4.422777	-0.066744
55	1	0	-5.129207	3.987454	1.334289

Visualization of calculated geometry of (**4b*E***):

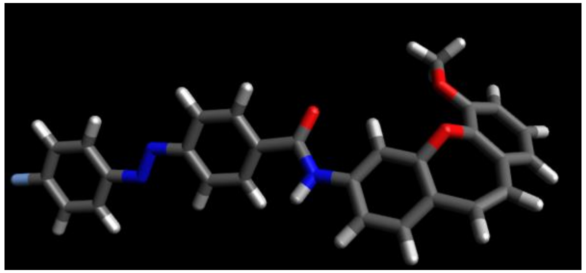

- The calculated coordinates of (**4b*Z***) (the part of calculated log file) -

Standard orientation:

Center Number	Atomic Number	Atomic Type	Coordinates (Angstroms) X	Y	Z
1	6	0	1.474593	-1.260341	-0.561295
2	6	0	1.842704	-2.511758	-1.085257
3	6	0	3.124288	-3.004533	-0.887145
4	6	0	4.096272	-2.281753	-0.171082
5	6	0	3.692465	-1.041696	0.357809
6	6	0	2.417560	-0.520547	0.168591
7	6	0	5.443842	-2.820393	-0.003315
8	6	0	6.586973	-2.126069	0.189917
9	6	0	6.740788	-0.670716	0.245532
10	6	0	5.700718	0.174993	0.654838
11	8	0	4.536406	-0.340745	1.210784
12	6	0	7.958620	-0.083843	-0.154530
13	6	0	8.107635	1.296480	-0.167060
14	6	0	7.055192	2.133700	0.217408
15	6	0	5.839083	1.576519	0.630297
16	7	0	0.161630	-0.815711	-0.808181
17	6	0	-0.478077	0.299995	-0.313849
18	8	0	0.033946	1.079447	0.487135
19	6	0	-1.878775	0.505175	-0.813150
20	6	0	-2.331432	0.060480	-2.064785
21	6	0	-3.631766	0.329295	-2.481896
22	6	0	-4.516812	0.999368	-1.627685
23	6	0	-4.065358	1.473386	-0.385034
24	6	0	-2.753075	1.238450	0.003827
25	7	0	-5.796460	1.363082	-2.161354
26	6	0	-7.024210	0.450840	-0.348024
27	6	0	-6.433737	-0.805619	-0.133232
28	6	0	-6.742847	-1.531989	1.013037

29	6	0	-7.625036	-0.983250	1.939698
30	6	0	-8.236976	0.250926	1.743387
31	6	0	-7.954489	0.952008	0.574938
32	7	0	-6.881601	1.204131	-1.558467
33	9	0	-7.910947	-1.682106	3.056724
34	8	0	4.754700	2.290779	1.040473
35	6	0	4.846690	3.710772	1.020573
36	1	0	1.118630	-3.098800	-1.645782
37	1	0	3.390248	-3.974016	-1.300979
38	1	0	2.164055	0.433840	0.604891
39	1	0	5.529222	-3.899067	-0.125803
40	1	0	7.517895	-2.689803	0.210836
41	1	0	8.776659	-0.727876	-0.465827
42	1	0	9.049838	1.738615	-0.478855
43	1	0	7.192669	3.208708	0.204113
44	1	0	-0.408158	-1.457788	-1.344183
45	1	0	-1.661588	-0.454621	-2.748506
46	1	0	-3.972266	0.022494	-3.466501
47	1	0	-4.735415	2.033879	0.259101
48	1	0	-2.384941	1.616010	0.952098
49	1	0	-5.755995	-1.226752	-0.867353
50	1	0	-6.311919	-2.511511	1.194406
51	1	0	-8.933250	0.633535	2.482671
52	1	0	-8.439825	1.901732	0.370227
53	1	0	3.878814	4.075047	1.368835
54	1	0	5.635074	4.072426	1.692891
55	1	0	5.034380	4.087274	0.006997

Visualization of calculated geometry of (**4b*Z***):

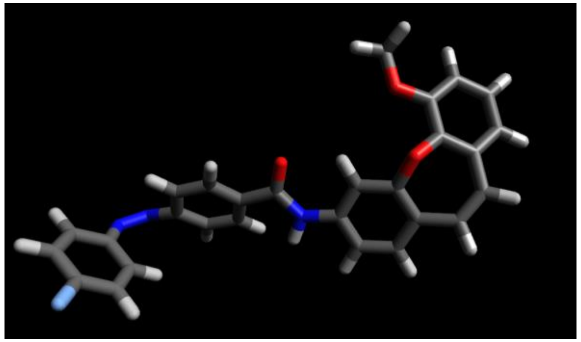

- The calculated coordinates of (**4c*E***) (the part of calculated log file) -

Standard orientation:

Center Number	Atomic Number	Atomic Type	Coordinates (Angstroms) X	Y	Z
1	6	0	2.326308	1.322534	-0.562815
2	6	0	2.671489	2.684634	-0.525721
3	6	0	4.004067	3.069444	-0.536867
4	6	0	5.050231	2.129117	-0.578109
5	6	0	4.672684	0.775206	-0.637460
6	6	0	3.346288	0.360253	-0.620482
7	6	0	6.441631	2.572876	-0.551125
8	6	0	7.512937	1.886443	-0.096443
9	6	0	7.522540	0.544628	0.489237
10	6	0	6.553383	-0.423963	0.171994
11	8	0	5.632912	-0.210256	-0.845820
12	6	0	8.515134	0.201476	1.426921
13	6	0	8.523529	-1.045875	2.044646
14	6	0	7.543879	-1.989055	1.729766
15	6	0	6.553847	-1.681408	0.793429
16	7	0	0.956329	1.000314	-0.537083
17	6	0	0.359197	-0.236758	-0.643130
18	8	0	0.983124	-1.278856	-0.833057
19	6	0	-1.137944	-0.236830	-0.523086
20	6	0	-1.832106	-1.287778	-1.138122
21	6	0	-3.217746	-1.357558	-1.055284
22	6	0	-3.934284	-0.386987	-0.340152
23	6	0	-3.243172	0.657125	0.298387
24	6	0	-1.859754	0.727618	0.204468
25	7	0	-5.342731	-0.564369	-0.320489
26	7	0	-5.961557	0.323439	0.332809
27	6	0	-7.362070	0.210326	0.394997
28	6	0	-7.995305	1.219113	1.146508
29	6	0	-9.372662	1.265208	1.316597
30	6	0	-10.136572	0.270254	0.713730
31	6	0	-9.569584	-0.751570	-0.041729
32	6	0	-8.190696	-0.772362	-0.193872
33	9	0	-11.472456	0.289249	0.860643
34	9	0	-7.669974	-1.765112	-0.926246
35	8	0	5.571641	-2.604696	0.519587
36	6	0	5.720885	-3.295214	-0.727149
37	1	0	1.890730	3.440997	-0.490108
38	1	0	4.251286	4.127568	-0.504797
39	1	0	3.115515	-0.693059	-0.673395
40	1	0	6.604127	3.603893	-0.861251
41	1	0	8.467680	2.408509	-0.069504
42	1	0	9.275889	0.937328	1.674726

43	1	0	9.295532	-1.288335	2.769435
44	1	0	7.529883	-2.970351	2.194983
45	1	0	0.334287	1.798407	-0.520283
46	1	0	-1.268218	-2.040706	-1.678362
47	1	0	-3.770580	-2.158740	-1.536524
48	1	0	-3.802538	1.392407	0.865695
49	1	0	-1.341146	1.522799	0.734550
50	1	0	-7.356732	1.972408	1.596966
51	1	0	-9.853653	2.044757	1.897275
52	1	0	-10.184144	-1.516500	-0.503518
53	1	0	4.887899	-3.999437	-0.786227
54	1	0	5.675174	-2.600782	-1.571927
55	1	0	6.668375	-3.848996	-0.754465

Visualization of calculated geometry of (**4c*E***):

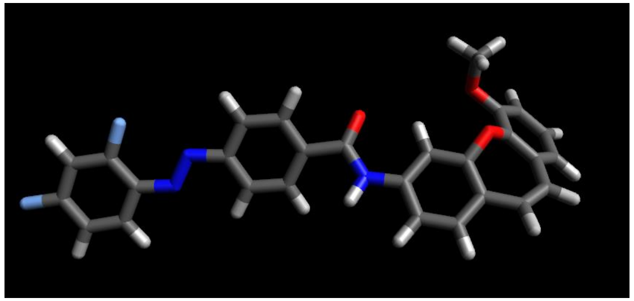

- The calculated coordinates of (**4c*Z***) (the part of calculated log file) -

Standard orientation:

Center Number	Atomic Number	Atomic Type	Coordinates (Angstroms) X	Y	Z
1	6	0	1.424415	0.965766	-0.067314
2	6	0	1.530210	2.346319	0.174307
3	6	0	2.760297	2.980906	0.083719
4	6	0	3.936717	2.280921	-0.242013
5	6	0	3.795037	0.905206	-0.499685
6	6	0	2.577016	0.241633	-0.408783
7	6	0	5.219282	2.978211	-0.293702
8	6	0	6.447377	2.452387	-0.091669
9	6	0	6.773192	1.064389	0.241429
10	6	0	5.951531	-0.014045	-0.133130
11	8	0	4.871598	0.170220	-0.986669
12	6	0	7.933353	0.781095	0.986956
13	6	0	8.246094	-0.520668	1.367933
14	6	0	7.410473	-1.576879	1.001113
15	6	0	6.258962	-1.327600	0.250582

16	7	0	0.147986	0.383821	0.051638
17	6	0	-0.236104	-0.912760	-0.209879
18	8	0	0.526923	-1.770727	-0.649198
19	6	0	-1.679217	-1.225637	0.066251
20	6	0	-2.467513	-0.528622	0.994073
21	6	0	-3.794651	-0.889278	1.214519
22	6	0	-4.345622	-1.970984	0.512876
23	6	0	-3.548248	-2.707889	-0.374820
24	6	0	-2.235188	-2.324918	-0.607235
25	7	0	-5.638961	-2.497601	0.813397
26	6	0	-6.754618	-0.430049	0.502189
27	6	0	-7.482330	0.433808	1.335097
28	6	0	-7.710673	1.759148	0.980685
29	6	0	-7.254312	2.199896	-0.258698
30	6	0	-6.579872	1.365839	-1.143567
31	6	0	-6.340583	0.058396	-0.745750
32	9	0	-7.485595	3.473079	-0.627356
33	7	0	-6.679197	-1.806698	0.895189
34	9	0	-5.720512	-0.769088	-1.607831
35	8	0	5.423055	-2.371324	-0.071605
36	6	0	5.511012	-2.819870	-1.429718
37	1	0	0.643686	2.921437	0.431946
38	1	0	2.821972	4.048921	0.276847
39	1	0	2.528026	-0.815415	-0.622610
40	1	0	5.154016	4.056242	-0.432093
41	1	0	7.291004	3.140060	-0.081010
42	1	0	8.582204	1.603894	1.276177
43	1	0	9.143720	-0.716400	1.947647
44	1	0	7.633418	-2.601804	1.282822
45	1	0	-0.591191	1.033458	0.286401
46	1	0	-2.048650	0.279784	1.587817
47	1	0	-4.384467	-0.358710	1.954538
48	1	0	-3.975872	-3.569180	-0.879367
49	1	0	-1.610723	-2.881031	-1.298133
50	1	0	-7.851796	0.046078	2.279849
51	1	0	-8.248079	2.438309	1.633708
52	1	0	-6.255320	1.717380	-2.116745
53	1	0	4.813220	-3.655503	-1.519789
54	1	0	5.225567	-2.026545	-2.127725
55	1	0	6.526613	-3.167231	-1.659992

Visualization of calculated geometry of (**4c*Z***):

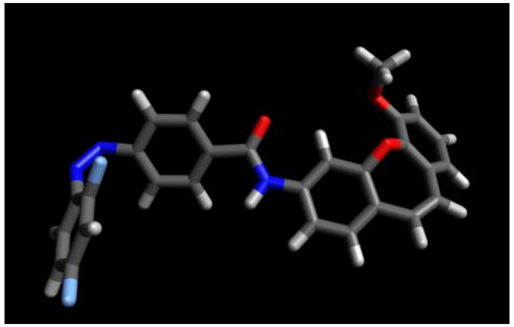

- The calculated coordinates of (**4d*E***) (the part of calculated log file) -

Standard orientation:

Center Number	Atomic Number	Atomic Type	Coordinates (Angstroms) X	Y	Z
1	6	0	-2.181533	-1.348633	-0.574970
2	6	0	-2.508206	-2.709137	-0.439627
3	6	0	-3.835318	-3.113146	-0.433014
4	6	0	-4.893186	-2.192917	-0.551523
5	6	0	-4.534313	-0.841002	-0.706770
6	6	0	-3.213495	-0.407371	-0.710148
7	6	0	-6.279916	-2.650615	-0.509721
8	6	0	-7.364437	-1.946135	-0.117581
9	6	0	-7.393701	-0.569607	0.381388
10	6	0	-6.429234	0.378488	0.013604
11	8	0	-5.508074	0.108956	-0.991035
12	6	0	-8.400572	-0.179032	1.287673
13	6	0	-8.414303	1.103474	1.819323
14	6	0	-7.432647	2.035293	1.466267
15	6	0	-6.428511	1.677160	0.559062
16	7	0	-0.815821	-1.006107	-0.561190
17	6	0	-0.232793	0.221182	-0.787406
18	8	0	-0.865833	1.231254	-1.087489
19	6	0	1.263390	0.251347	-0.654754
20	6	0	1.954072	1.233484	-1.377849
21	6	0	3.338115	1.326971	-1.289802
22	6	0	4.055328	0.450302	-0.462879
23	6	0	3.367439	-0.522327	0.283210
24	6	0	1.985979	-0.617263	0.184310
25	7	0	5.461704	0.642190	-0.448012
26	7	0	6.076355	-0.172692	0.298606
27	6	0	7.471024	-0.038793	0.367330
28	6	0	8.130533	-0.988384	1.178855
29	6	0	9.503674	-1.006543	1.367696
30	6	0	10.277259	-0.037868	0.727796
31	6	0	9.680840	0.928367	-0.084884
32	6	0	8.304052	0.919697	-0.256217
33	9	0	7.767124	1.864293	-1.039955
34	9	0	7.386605	-1.919807	1.795285
35	8	0	-5.434924	2.503749	0.130232
36	6	0	-5.389542	3.822636	0.663040
37	1	0	-1.717194	-3.449405	-0.342928

38	1	0	-4.068045	-4.169633	-0.325639
39	1	0	-2.999712	0.642964	-0.838977
40	1	0	-6.427547	-3.702251	-0.750705
41	1	0	-8.314461	-2.475217	-0.068455
42	1	0	-9.159964	-0.901443	1.574586
43	1	0	-9.193517	1.393742	2.518591
44	1	0	-7.463143	3.032957	1.888622
45	1	0	-0.184032	-1.790556	-0.462075
46	1	0	1.389123	1.914854	-2.005011
47	1	0	3.888583	2.074763	-1.852743
48	1	0	3.928369	-1.182449	0.935090
49	1	0	1.468635	-1.352522	0.795625
50	1	0	9.943375	-1.766811	2.004148
51	1	0	11.354518	-0.033945	0.862806
52	1	0	10.265396	1.690802	-0.589165
53	1	0	-4.522455	4.298489	0.202049
54	1	0	-6.293943	4.390095	0.409249
55	1	0	-5.262573	3.812023	1.753030

Visualization of calculated geometry of (**4d*E***) :

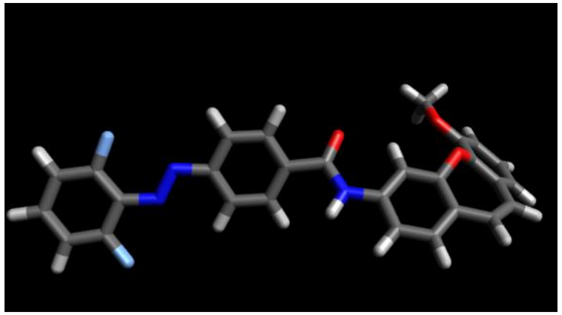

- The calculated coordinates of (**4d*Z***) (the part of calculated log file) -

Standard orientation:

Center Number	Atomic Number	Atomic Type	Coordinates (Angstroms) X	Y	Z
1	6	0	1.492913	1.031530	-0.144554
2	6	0	1.642529	2.387549	0.193058
3	6	0	2.893492	2.985253	0.149676
4	6	0	4.048770	2.270928	-0.217236
5	6	0	3.865847	0.919783	-0.564764
6	6	0	2.624177	0.295134	-0.528368
7	6	0	5.350487	2.933233	-0.229459
8	6	0	6.564808	2.361724	-0.075617
9	6	0	6.856254	0.947359	0.164316

10	6	0	5.996427	-0.084679	-0.257737
11	8	0	4.916879	0.177207	-1.089335
12	6	0	8.021166	0.590268	0.867679
13	6	0	8.311494	-0.740703	1.156198
14	6	0	7.442053	-1.750287	0.744000
15	6	0	6.280399	-1.425810	0.036204
16	7	0	0.196986	0.484846	-0.079268
17	6	0	-0.220624	-0.786384	-0.406533
18	8	0	0.523706	-1.649270	-0.866996
19	6	0	-1.679901	-1.062518	-0.177665
20	6	0	-2.457406	-0.399333	0.783784
21	6	0	-3.801156	-0.723239	0.958361
22	6	0	-4.378616	-1.731091	0.173152
23	6	0	-3.594621	-2.436442	-0.750881
24	6	0	-2.263630	-2.090560	-0.934556
25	7	0	-5.701087	-2.217767	0.405963
26	6	0	-6.696372	-0.081804	0.221482
27	6	0	-7.356844	0.788355	1.100408
28	6	0	-7.565473	2.128371	0.814626
29	6	0	-7.143525	2.621497	-0.422584
30	6	0	-6.517430	1.784839	-1.347841
31	6	0	-6.306516	0.454304	-1.014282
32	7	0	-6.706522	-1.483571	0.523242
33	9	0	-5.733422	-0.365591	-1.916876
34	9	0	-7.758635	0.290193	2.284708
35	8	0	5.448191	-2.412385	-0.441160
36	6	0	4.618405	-3.024775	0.550592
37	1	0	0.774147	2.973368	0.485689
38	1	0	2.988666	4.035757	0.412631
39	1	0	2.537865	-0.738703	-0.827176
40	1	0	5.314826	4.019419	-0.296253
41	1	0	7.426997	3.024377	-0.028394
42	1	0	8.697669	1.377229	1.191145
43	1	0	9.219593	-0.994564	1.695586
44	1	0	7.662535	-2.796064	0.938940
45	1	0	-0.527274	1.144054	0.174851
46	1	0	-2.017806	0.350288	1.436777
47	1	0	-4.383759	-0.225620	1.726610
48	1	0	-4.046235	-3.241806	-1.322386
49	1	0	-1.648044	-2.622794	-1.651811
50	1	0	-8.055148	2.759935	1.548286
51	1	0	-7.306788	3.666074	-0.668358
52	1	0	-6.198441	2.142799	-2.321150
53	1	0	4.059957	-3.812262	0.039773
54	1	0	5.216012	-3.467612	1.357408

55	1	0	3.915255	-2.299283	0.978950

Visualization of calculated geometry of (**4d*Z***) :

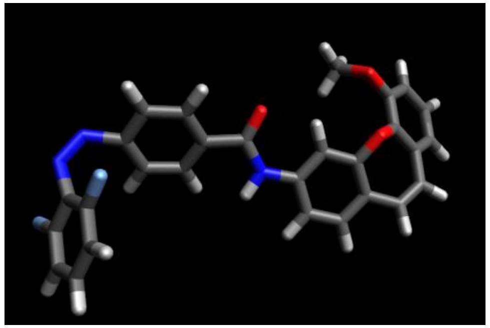

- The calculated coordinates of (**4e*E***) (the part of calculated log file) -

Standard orientation:

Center Number	Atomic Number	Atomic Type	Coordinates (Angstroms) X	Y	Z
1	6	0	-1.399821	-1.562378	-0.248220
2	6	0	-1.702936	-2.843196	0.242808
3	6	0	-3.021339	-3.268014	0.316838
4	6	0	-4.095852	-2.450640	-0.081551
5	6	0	-3.760035	-1.180992	-0.587798
6	6	0	-2.447364	-0.728251	-0.667130
7	6	0	-5.467419	-2.935060	0.049312
8	6	0	-6.588798	-2.194309	0.187044
9	6	0	-6.694794	-0.738343	0.229912
10	6	0	-5.747537	0.129656	-0.355144
11	8	0	-4.722946	-0.355767	-1.160166
12	6	0	-7.780000	-0.131863	0.882801
13	6	0	-7.923569	1.251110	0.983925
14	6	0	-6.953806	2.081378	0.407707
15	6	0	-5.863693	1.507601	-0.265352
16	8	0	-6.979597	3.442270	0.430299
17	7	0	-0.043319	-1.187170	-0.291844
18	6	0	0.515488	-0.024132	-0.772272
19	8	0	-0.135039	0.875893	-1.301133
20	6	0	2.006322	0.079545	-0.622222
21	6	0	-8.069747	4.085095	1.084325
22	6	0	2.685786	0.913086	-1.521236
23	6	0	4.064399	1.065874	-1.432971

24	6	0	4.788133	0.400631	-0.432860
25	6	0	4.110822	-0.418828	0.486468
26	6	0	2.734678	-0.574910	0.388526
27	7	0	6.187784	0.633068	-0.434542
28	7	0	6.822765	0.020138	0.467546
29	6	0	8.220864	0.246919	0.476783
30	6	0	8.931641	-0.438683	1.472572
31	6	0	10.313825	-0.289852	1.575509
32	6	0	10.990116	0.545265	0.683865
33	6	0	10.281625	1.231903	-0.312113
34	6	0	8.903747	1.087924	-0.421066
35	1	0	-0.902207	-3.505667	0.563706
36	1	0	-3.236836	-4.262044	0.700712
37	1	0	-2.243458	0.248679	-1.078864
38	1	0	-5.569455	-4.016331	0.123964
39	1	0	-7.520705	-2.728898	0.363711
40	1	0	-8.535922	-0.770737	1.333305
41	1	0	-8.782686	1.661995	1.500971
42	1	0	-5.125874	2.145195	-0.740454
43	1	0	0.603591	-1.907966	0.001479
44	1	0	-7.887238	5.156177	0.983471
45	1	0	-9.026205	3.831965	0.610314
46	1	0	-8.110209	3.820851	2.148324
47	1	0	2.116036	1.432352	-2.284391
48	1	0	4.605481	1.699123	-2.129792
49	1	0	4.675056	-0.913115	1.269220
50	1	0	2.225792	-1.185023	1.130871
51	1	0	8.379249	-1.080941	2.152376
52	1	0	10.861139	-0.822580	2.348085
53	1	0	12.067554	0.664259	0.761112
54	1	0	10.812619	1.880940	-1.003467
55	1	0	8.339917	1.611121	-1.185540

Visualization of calculated geometry of (**4e*E***) :

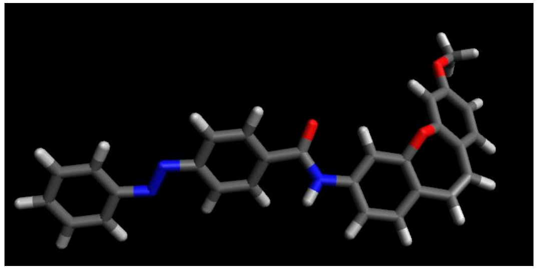

- The calculated coordinates of (**4e*Z***) (the part of calculated log file) -

```
                 Standard orientation:
---------------------------------------------------------------------
Center     Atomic      Atomic              Coordinates (Angstroms)
```

Number	Number	Type	X	Y	Z
1	6	0	-0.745372	1.100202	0.065066
2	6	0	-0.853788	2.425042	-0.388979
3	6	0	-2.078956	3.074782	-0.360116
4	6	0	-3.248688	2.448327	0.109466
5	6	0	-3.106155	1.126665	0.572270
6	6	0	-1.890558	0.450996	0.550399
7	6	0	-4.518890	3.169032	0.090196
8	6	0	-5.760910	2.640907	0.030981
9	6	0	-6.126280	1.227969	-0.023042
10	6	0	-5.305786	0.191344	0.471977
11	8	0	-4.156077	0.467716	1.203958
12	6	0	-7.346937	0.839734	-0.598290
13	6	0	-7.739453	-0.493319	-0.708551
14	6	0	-6.890573	-1.496654	-0.224250
15	6	0	-5.669207	-1.142015	0.370184
16	8	0	-7.158447	-2.830803	-0.265891
17	7	0	0.526702	0.498474	0.013961
18	6	0	0.903926	-0.761352	0.421398
19	8	0	0.135701	-1.562438	0.951582
20	6	0	2.348136	-1.103292	0.194051
21	6	0	-8.391188	-3.252644	-0.842042
22	6	0	3.137582	-0.533024	-0.817017
23	6	0	4.469316	-0.904415	-0.976086
24	6	0	5.029274	-1.868771	-0.124925
25	6	0	4.231592	-2.485096	0.850348
26	6	0	2.911600	-2.088394	1.019372
27	7	0	6.329477	-2.428581	-0.352161
28	6	0	7.452181	-0.340721	-0.289488
29	6	0	8.228374	0.391988	-1.198516
30	6	0	8.444866	1.751902	-0.987904
31	6	0	7.943697	2.371095	0.160527
32	6	0	7.216726	1.626019	1.093520
33	6	0	6.958863	0.274763	0.871483
34	7	0	7.368171	-1.752726	-0.523406

35	1	0	0.025275	2.945418	-0.762601
36	1	0	-2.143332	4.099688	-0.716834
37	1	0	-1.831780	-0.556519	0.933701
38	1	0	-4.432916	4.253046	0.038447
39	1	0	-6.593865	3.335561	-0.064763
40	1	0	-8.009388	1.613690	-0.978692
41	1	0	-8.693471	-0.732799	-1.163182
42	1	0	-5.021587	-1.912056	0.775465
43	1	0	1.268347	1.107801	-0.305400
44	1	0	-8.393878	-4.341743	-0.774415
45	1	0	-9.249152	-2.850674	-0.288938
46	1	0	-8.464224	-2.952455	-1.894695
47	1	0	2.714839	0.181959	-1.518564
48	1	0	5.064133	-0.467533	-1.771500
49	1	0	4.661586	-3.266406	1.470556
50	1	0	2.288962	-2.549655	1.778587
51	1	0	8.641364	-0.114321	-2.066476
52	1	0	9.023842	2.323164	-1.708236
53	1	0	8.133071	3.426274	0.336876
54	1	0	6.848229	2.098729	1.999859
55	1	0	6.403710	-0.303557	1.602667

Visualization of calculated geometry of (**4e*Z***) :

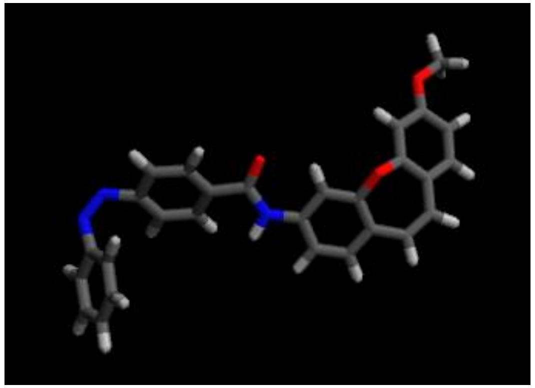

• The calculated coordinates of (**4f*E***) (the part of calculated log file) -

Standard orientation:

Center Number	Atomic Number	Atomic Type	Coordinates (Angstroms) X	Y	Z
1	6	0	1.821267	-1.577878	0.248300
2	6	0	2.131184	-2.850997	-0.258309
3	6	0	3.452445	-3.263989	-0.346851
4	6	0	4.523238	-2.441632	0.051283

5	6	0	4.180887	-1.179888	0.572707
6	6	0	2.865129	-0.739212	0.667322
7	6	0	5.897724	-2.913288	-0.094911
8	6	0	7.011664	-2.161810	-0.234750
9	6	0	7.104782	-0.704653	-0.265107
10	6	0	6.155046	0.149696	0.335743
11	8	0	5.141581	-0.351969	1.144927
12	6	0	8.178949	-0.082878	-0.921858
13	6	0	8.309381	1.302143	-1.012086
14	6	0	7.337337	2.118658	-0.420294
15	6	0	6.258277	1.529354	0.257157
16	8	0	7.350776	3.479877	-0.431370
17	7	0	0.462067	-1.214531	0.306564
18	6	0	-0.102439	-0.061500	0.804191
19	8	0	0.544695	0.837637	1.338697
20	6	0	-1.595007	0.032227	0.665670
21	6	0	8.428072	4.138076	-1.091407
22	6	0	-2.274795	0.849546	1.579270
23	6	0	-3.654989	0.993111	1.501837
24	6	0	-4.380298	0.335297	0.497798
25	6	0	-3.702918	-0.467951	-0.435803
26	6	0	-2.325121	-0.615194	-0.348304
27	7	0	-5.780891	0.557561	0.511124
28	7	0	-6.417223	-0.043438	-0.398656
29	6	0	-7.814104	0.172917	-0.395203
30	6	0	-8.528856	-0.492792	-1.402769
31	6	0	-9.910860	-0.355806	-1.500911
32	6	0	-10.558477	0.455855	-0.576026
33	6	0	-9.876229	1.131108	0.437752
34	6	0	-8.498779	0.987785	0.526984
35	9	0	-11.896133	0.598385	-0.658541
36	1	0	1.333493	-3.516902	-0.579668
37	1	0	3.673135	-4.252301	-0.742366
38	1	0	2.656417	0.231663	1.090810
39	1	0	6.008176	-3.992930	-0.180400
40	1	0	7.946642	-2.686747	-0.423754
41	1	0	8.936496	-0.711070	-1.384486
42	1	0	9.160183	1.725190	-1.533029
43	1	0	5.518882	2.156165	0.744009
44	1	0	-0.181193	-1.937430	0.010365
45	1	0	8.236315	5.206559	-0.980614
46	1	0	9.391692	3.890744	-0.628974
47	1	0	8.460280	3.882196	-2.157726
48	1	0	-1.703962	1.363704	2.345070
49	1	0	-4.196128	1.613558	2.210024

50	1	0	-4.268086	-0.956860	-1.221255
51	1	0	-1.816603	-1.212502	-1.101226
52	1	0	-7.980008	-1.115628	-2.102589
53	1	0	-10.483246	-0.861090	-2.272020
54	1	0	-10.431273	1.752317	1.133896
55	1	0	-7.935161	1.496555	1.300982

Visualization of calculated geometry of (**4f*E***) :

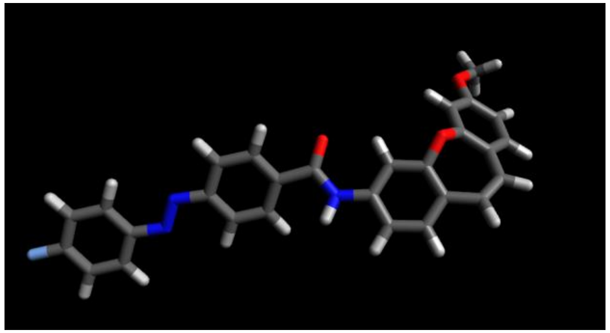

- The calculated coordinates of (**4f*Z***) (the part of calculated log file) -

Standard orientation:

Center Number	Atomic Number	Atomic Type	Coordinates (Angstroms) X	Y	Z
1	6	0	0.995386	1.038660	-0.034195
2	6	0	1.068717	2.355041	0.450824
3	6	0	2.275446	3.038805	0.434916
4	6	0	3.460925	2.455919	-0.050920
5	6	0	3.353017	1.142848	-0.546354
6	6	0	2.156663	0.433461	-0.537651
7	6	0	4.711124	3.210229	-0.016509
8	6	0	5.967062	2.714458	0.027815
9	6	0	6.370105	1.310924	0.047078
10	6	0	5.577115	0.265084	-0.472716
11	8	0	4.418668	0.527940	-1.195798
12	6	0	7.602196	0.941729	0.610350
13	6	0	8.030273	-0.382670	0.688181
14	6	0	7.207648	-1.396318	0.180649
15	6	0	5.976109	-1.060126	-0.403351
16	8	0	7.510206	-2.723603	0.190592
17	7	0	-0.257804	0.398007	0.012520
18	6	0	-0.607144	-0.850025	-0.452120
19	8	0	0.171979	-1.597322	-1.041697
20	6	0	-2.032200	-1.253527	-0.205365
21	6	0	8.753452	-3.127219	0.757143

22	6	0	-2.824356	-0.739461	0.833211
23	6	0	-4.135265	-1.171920	1.010667
24	6	0	-4.670153	-2.143735	0.152076
25	6	0	-3.867054	-2.705480	-0.851138
26	6	0	-2.568963	-2.248425	-1.037083
27	7	0	-5.940288	-2.760156	0.399535
28	6	0	-7.162275	-0.728591	0.328158
29	6	0	-8.018067	-0.048151	1.207496
30	6	0	-8.313121	1.296549	1.004912
31	6	0	-7.793469	1.926373	-0.121797
32	6	0	-6.989400	1.260100	-1.042405
33	6	0	-6.663871	-0.072932	-0.809941
34	9	0	-8.094787	3.222158	-0.340950
35	7	0	-7.008812	-2.131980	0.570397
36	1	0	0.176412	2.842456	0.837287
37	1	0	2.312234	4.056763	0.814654
38	1	0	2.125324	-0.564876	-0.947054
39	1	0	4.596187	4.289879	0.062583
40	1	0	6.781245	3.428645	0.139623
41	1	0	8.244786	1.723696	1.007997
42	1	0	8.991610	-0.607245	1.134880
43	1	0	5.348969	-1.837133	-0.827019
44	1	0	-1.010363	0.971375	0.370635
45	1	0	8.784665	-4.213848	0.662440
46	1	0	9.600905	-2.689477	0.215158
47	1	0	8.818244	-2.851554	1.817009
48	1	0	-2.419944	-0.020969	1.541729
49	1	0	-4.732088	-0.777225	1.826612
50	1	0	-4.275501	-3.491740	-1.479571
51	1	0	-1.942338	-2.667902	-1.816887
52	1	0	-8.435196	-0.583102	2.055560
53	1	0	-8.951832	1.848319	1.687052
54	1	0	-6.630806	1.783959	-1.922747
55	1	0	-6.047323	-0.606255	-1.524923

Visualization of calculated geometry of (**4fZ**) :

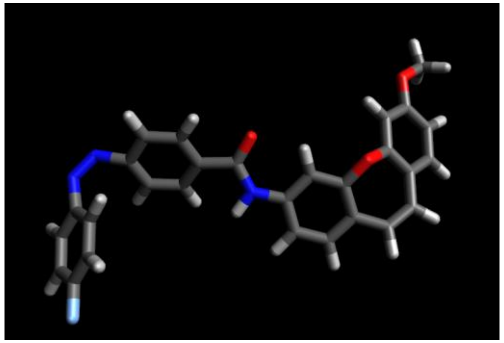

- The calculated coordinates of (**4g*E***) (the part of calculated log file) -

Standard orientation:

Center Number	Atomic Number	Atomic Type	Coordinates (Angstroms) X	Y	Z
1	6	0	-2.088998	-1.588439	-0.297994
2	6	0	-2.404304	-2.875484	0.168516
3	6	0	-3.727385	-3.284846	0.246026
4	6	0	-4.794739	-2.445104	-0.124095
5	6	0	-4.447055	-1.169100	-0.605718
6	6	0	-3.129318	-0.731961	-0.688775
7	6	0	-6.171374	-2.914408	0.009035
8	6	0	-7.281239	-2.162247	0.175343
9	6	0	-7.366294	-0.706416	0.254393
10	6	0	-6.412943	0.161950	-0.320037
11	8	0	-5.405076	-0.318787	-1.148834
12	6	0	-8.434987	-0.100713	0.934664
13	6	0	-8.556319	1.281252	1.072250
14	6	0	-7.580376	2.111341	0.506247
15	6	0	-6.506973	1.538739	-0.194116
16	8	0	-7.584859	3.471406	0.564292
17	7	0	-0.728225	-1.229582	-0.345837
18	6	0	-0.158240	-0.065746	-0.811012
19	8	0	-0.800631	0.850587	-1.321409
20	6	0	1.334537	0.017417	-0.667297
21	6	0	-8.656147	4.113256	1.249683
22	6	0	2.021078	0.852281	-1.559659
23	6	0	3.401916	0.986326	-1.475136
24	6	0	4.120669	0.302261	-0.484009
25	6	0	3.436402	-0.519207	0.428423
26	6	0	2.058221	-0.657599	0.333225
27	7	0	5.523571	0.520137	-0.491764
28	7	0	6.142567	-0.098639	0.420375
29	6	0	7.537810	0.070269	0.473648
30	6	0	8.170533	-0.636129	1.514815
31	6	0	9.543000	-0.586165	1.719786
32	6	0	10.302327	0.195158	0.853849
33	6	0	9.735584	0.915138	-0.193416
34	6	0	8.361641	0.846411	-0.373659
35	9	0	11.633434	0.262725	1.027737
36	9	0	7.840966	1.547797	-1.388552
37	1	0	-1.609314	-3.554993	0.467306

38	1	0	-3.952255	-4.284154	0.610316
39	1	0	-2.916458	0.250734	-1.081858
40	1	0	-6.287181	-3.995701	0.059286
41	1	0	-8.218618	-2.688303	0.348672
42	1	0	-9.195311	-0.739492	1.377831
43	1	0	-9.403275	1.691733	1.609337
44	1	0	-5.764821	2.177074	-0.661405
45	1	0	-0.088185	-1.963435	-0.070338
46	1	0	-8.457599	5.183673	1.175683
47	1	0	-9.622493	3.888429	0.781473
48	1	0	-8.687494	3.820489	2.306499
49	1	0	1.455219	1.387207	-2.314883
50	1	0	3.949043	1.619624	-2.167023
51	1	0	3.996119	-1.029487	1.204095
52	1	0	1.544182	-1.270163	1.069964
53	1	0	7.535577	-1.231373	2.163390
54	1	0	10.023664	-1.132521	2.523952
55	1	0	10.346582	1.517487	-0.856658

Visualization of calculated geometry of (**4g*E***) :

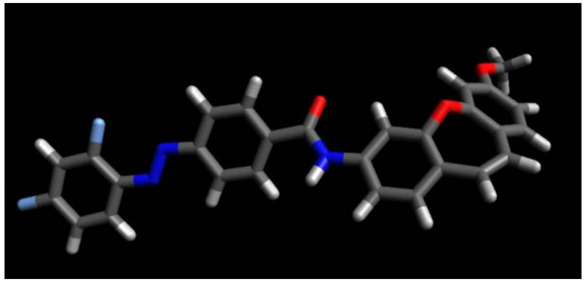

• The calculated coordinates of (**4g*Z***) (the part of calculated log file) -

Standard orientation:

Center Number	Atomic Number	Atomic Type	Coordinates (Angstroms) X	Y	Z
1	6	0	-1.172407	1.047038	0.015728
2	6	0	-1.251608	2.373178	-0.440692
3	6	0	-2.459615	3.053744	-0.401276
4	6	0	-3.640198	2.458273	0.081297
5	6	0	-3.525882	1.135670	0.549301
6	6	0	-2.328356	0.428930	0.515714
7	6	0	-4.892183	3.210208	0.070131
8	6	0	-6.147517	2.712794	0.026849

9	6	0	-6.549151	1.309319	-0.012025
10	6	0	-5.750373	0.255461	0.482274
11	8	0	-4.583989	0.505962	1.196725
12	6	0	-7.787309	0.948695	-0.567579
13	6	0	-8.216513	-0.374300	-0.661199
14	6	0	-7.388584	-1.395676	-0.178378
15	6	0	-6.150437	-1.068499	0.396706
16	8	0	-7.691936	-2.722512	-0.204579
17	7	0	0.082305	0.410887	-0.052152
18	6	0	0.429810	-0.858240	0.351318
19	8	0	-0.350970	-1.634492	0.899732
20	6	0	1.857147	-1.249187	0.092047
21	6	0	-8.942652	-3.116888	-0.761178
22	6	0	2.654979	-0.680894	-0.912409
23	6	0	3.967884	-1.103915	-1.104068
24	6	0	4.494804	-2.120376	-0.294666
25	6	0	3.685687	-2.734452	0.672248
26	6	0	2.387569	-2.287715	0.873862
27	7	0	5.770309	-2.712752	-0.545548
28	6	0	6.941063	-0.654606	-0.465318
29	6	0	7.676394	0.098462	-1.393502
30	6	0	7.936787	1.448821	-1.185475
31	6	0	7.505360	2.029809	0.004014
32	6	0	6.825675	1.310283	0.980616
33	6	0	6.553845	-0.026444	0.727470
34	9	0	7.767764	3.329816	0.231190
35	7	0	6.827724	-2.063154	-0.707762
36	9	0	5.926800	-0.743091	1.679107
37	1	0	-0.363377	2.870199	-0.824259
38	1	0	-2.501548	4.079197	-0.759719
39	1	0	-2.291232	-0.578141	0.902747
40	1	0	-4.779991	4.291259	0.008241
41	1	0	-6.963681	3.427432	-0.066026
42	1	0	-8.434337	1.736740	-0.945548
43	1	0	-9.182776	-0.591843	-1.100721
44	1	0	-5.518665	-1.851921	0.801266
45	1	0	0.835047	0.999978	-0.383184
46	1	0	-8.973477	-4.204781	-0.682282
47	1	0	-9.782711	-2.686731	-0.201883
48	1	0	-9.020761	-2.825358	-1.815878
49	1	0	2.254763	0.072544	-1.586051
50	1	0	4.564605	-0.673016	-1.901042
51	1	0	4.092936	-3.550133	1.262252
52	1	0	1.755172	-2.746537	1.626234
53	1	0	8.025370	-0.396064	-2.295206

54	1	0	8.479847	2.042162	-1.913180
55	1	0	6.521221	1.770993	1.913909

Visualization of calculated geometry of (**4g*Z***) :

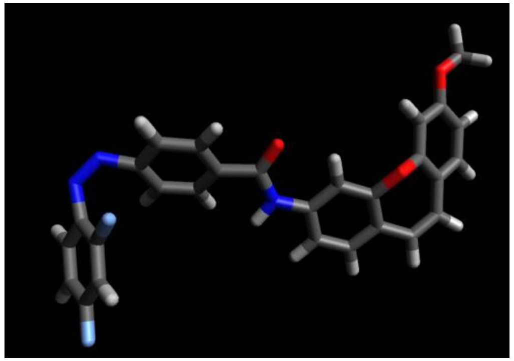

- The calculated coordinates of (**4h*E***) (the part of calculated log file) -

Standard orientation:

Center Number	Atomic Number	Atomic Type	Coordinates (Angstroms) X	Y	Z
1	6	0	-1.936176	-1.533954	-0.413642
2	6	0	-2.233276	-2.851716	-0.027994
3	6	0	-3.550632	-3.282950	0.025170
4	6	0	-4.629846	-2.436498	-0.291549
5	6	0	-4.300094	-1.128698	-0.693880
6	6	0	-2.988587	-0.669185	-0.749951
7	6	0	-5.999861	-2.931506	-0.186699
8	6	0	-7.119765	-2.205925	0.024641
9	6	0	-7.225234	-0.758843	0.191226
10	6	0	-6.284083	0.155844	-0.328999
11	8	0	-5.269727	-0.259939	-1.184517
12	6	0	-8.302629	-0.210207	0.905333
13	6	0	-8.443463	1.159162	1.125459
14	6	0	-7.478868	2.035399	0.611931
15	6	0	-6.397318	1.521105	-0.120429
16	8	0	-7.502537	3.389289	0.751917
17	7	0	-0.580181	-1.154941	-0.441989
18	6	0	-0.028508	0.048578	-0.819104
19	8	0	-0.686392	0.993727	-1.251206
20	6	0	1.464724	0.140629	-0.682029
21	6	0	-8.582332	3.973259	1.474711
22	6	0	2.129463	1.057112	-1.508300
23	6	0	3.509019	1.204658	-1.426175
24	6	0	4.247746	0.450271	-0.503216
25	6	0	3.585756	-0.454628	0.344599
26	6	0	2.208729	-0.604642	0.251795

27	7	0	5.646671	0.689220	-0.505984
28	7	0	6.283115	-0.007608	0.335862
29	6	0	7.671993	0.183049	0.390351
30	6	0	8.361021	-0.638815	1.309416
31	6	0	9.732884	-0.583854	1.500806
32	6	0	10.474384	0.330192	0.751741
33	6	0	9.847781	1.170732	-0.170401
34	6	0	8.473266	1.091086	-0.341321
35	9	0	7.907216	1.916183	-1.232071
36	9	0	7.648209	-1.518123	2.030375
37	1	0	-1.428729	-3.537616	0.227460
38	1	0	-3.761427	-4.305775	0.327257
39	1	0	-2.789559	0.338922	-1.080887
40	1	0	-6.101066	-4.015317	-0.201748
41	1	0	-8.049623	-2.754426	0.165832
42	1	0	-9.054196	-0.885059	1.308137
43	1	0	-9.296652	1.524674	1.684747
44	1	0	-5.664010	2.196736	-0.547518
45	1	0	0.070480	-1.899046	-0.225193
46	1	0	-8.399502	5.048971	1.464800
47	1	0	-9.545775	3.762682	0.994108
48	1	0	-8.608097	3.617353	2.512107
49	1	0	1.547721	1.644863	-2.210249
50	1	0	4.039487	1.902024	-2.067562
51	1	0	4.162422	-1.018853	1.068791
52	1	0	1.711868	-1.283881	0.940182
53	1	0	10.196408	-1.246937	2.223219
54	1	0	11.550001	0.389660	0.886785
55	1	0	10.406852	1.889256	-0.760710

Visualization of calculated geometry of (**4h*E***) :

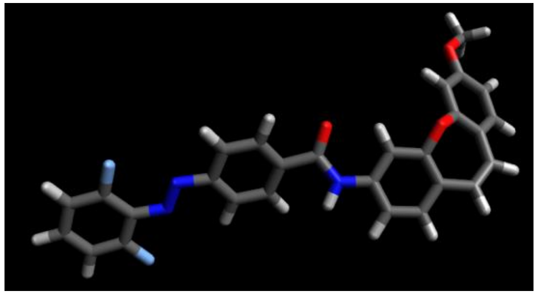

- The calculated coordinates of (**4h*Z***) (the part of calculated log file) -

Standard orientation:

Center Number	Atomic Number	Atomic Type	Coordinates (Angstroms) X	Y	Z
1	6	0	1.222293	1.126777	-0.057057
2	6	0	1.338397	2.455355	0.383813
3	6	0	2.568953	3.094873	0.354749

4	6	0	3.736107	2.454892	-0.102858
5	6	0	3.585278	1.130527	-0.555299
6	6	0	2.364637	0.464108	-0.530431
7	6	0	5.011246	3.166618	-0.084653
8	6	0	6.249504	2.630424	-0.019574
9	6	0	6.606328	1.215697	0.041865
10	6	0	5.780282	0.180685	-0.447385
11	8	0	4.630508	0.457169	-1.178948
12	6	0	7.826514	0.824222	0.615990
13	6	0	8.212974	-0.510102	0.732135
14	6	0	7.358603	-1.511600	0.253816
15	6	0	6.137517	-1.153851	-0.339338
16	8	0	7.620282	-2.846732	0.300819
17	7	0	-0.053344	0.532104	0.001144
18	6	0	-0.443121	-0.715523	-0.429049
19	8	0	0.315210	-1.512004	-0.979687
20	6	0	-1.887084	-1.059264	-0.192488
21	6	0	8.852874	-3.271656	0.875095
22	6	0	-2.682994	-0.464698	0.798344
23	6	0	-4.011014	-0.847770	0.968993
24	6	0	-4.553784	-1.849585	0.152562
25	6	0	-3.749782	-2.490019	-0.800806
26	6	0	-2.435816	-2.082449	-0.981783
27	7	0	-5.853594	-2.396033	0.380576
28	6	0	-6.927700	-0.298260	0.229896
29	6	0	-7.608315	0.537393	1.126286
30	6	0	-7.867614	1.872357	0.858475
31	6	0	-7.477661	2.394200	-0.377480
32	6	0	-6.832007	1.591199	-1.318995
33	6	0	-6.570543	0.265314	-1.003274
34	7	0	-6.886705	-1.704384	0.510910
35	9	0	-5.980199	-0.524210	-1.921667
36	9	0	-7.978061	0.011599	2.309237
37	1	0	0.461433	2.986637	0.746774
38	1	0	2.639593	4.123051	0.700638
39	1	0	2.300170	-0.545736	-0.906440
40	1	0	4.932716	4.251392	-0.037977
41	1	0	7.086599	3.320226	0.074899
42	1	0	8.493795	1.596836	0.990580
43	1	0	9.167180	-0.751772	1.185167
44	1	0	5.485996	-1.922624	-0.740721
45	1	0	-0.788347	1.145859	0.327149
46	1	0	8.851224	-4.360925	0.810505
47	1	0	9.710959	-2.874497	0.318774
48	1	0	8.929643	-2.968840	1.926712

49	1	0	-2.270801	0.276452	1.478262
50	1	0	-4.607145	-0.401384	1.758014
51	1	0	-4.172418	-3.293286	-1.396848
52	1	0	-1.805391	-2.560855	-1.723436
53	1	0	-8.370377	2.478019	1.605017
54	1	0	-7.681469	3.434833	-0.609356
55	1	0	-6.535954	1.971664	-2.291004

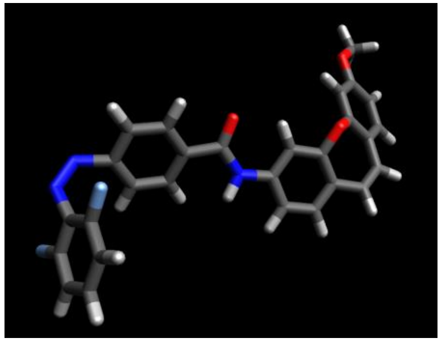

Visualization of calculated geometry of (**4h*Z***) :

- The calculated coordinates of (**5a*E***) (the part of calculated log file) -

Standard orientation:

Center Number	Atomic Number	Atomic Type	Coordinates (Angstroms) X	Y	Z
1	6	0	-1.639195	-1.939536	-0.387790
2	6	0	-2.406608	-3.085438	-0.659453
3	6	0	-3.740965	-2.965269	-1.019415
4	6	0	-4.374222	-1.712784	-1.119315
5	6	0	-3.574899	-0.583420	-0.862458
6	6	0	-2.237759	-0.674703	-0.492595
7	6	0	-5.788976	-1.625980	-1.472968
8	6	0	-6.656297	-0.641243	-1.150417
9	6	0	-6.382307	0.553919	-0.349640
10	6	0	-5.104162	1.123848	-0.270884
11	8	0	-4.075207	0.691494	-1.098301
12	6	0	-7.422137	1.144614	0.397050
13	6	0	-7.174430	2.244663	1.207068
14	6	0	-5.890357	2.791756	1.298559
15	6	0	-4.842140	2.231374	0.559058
16	7	0	-0.293997	-2.134368	-0.019148
17	6	0	0.680048	-1.191136	0.221873

18	8	0	0.502789	0.018342	0.097188
19	6	0	2.013873	-1.745186	0.644727
20	6	0	3.140429	-0.970720	0.364036
21	6	0	4.414609	-1.418157	0.739126
22	6	0	4.553829	-2.638067	1.415959
23	6	0	3.428873	-3.403431	1.716465
24	6	0	2.162412	-2.962809	1.333279
25	7	0	5.624332	-0.714879	0.491825
26	7	0	5.483107	0.369855	-0.135376
27	6	0	6.684074	1.080994	-0.382994
28	6	0	6.531645	2.287292	-1.081263
29	6	0	7.645953	3.069780	-1.380658
30	6	0	8.916531	2.648970	-0.982953
31	6	0	9.071125	1.443325	-0.284644
32	6	0	7.964664	0.658325	0.017296
33	8	0	-3.559622	2.688735	0.555777
34	6	0	-3.246425	3.799325	1.388590
35	1	0	-1.952088	-4.071175	-0.589829
36	1	0	-4.318617	-3.863253	-1.224013
37	1	0	-1.673258	0.227904	-0.313090
38	1	0	-6.191187	-2.497299	-1.987564
39	1	0	-7.699717	-0.782631	-1.426330
40	1	0	-8.420087	0.718472	0.339042
41	1	0	-7.982845	2.691120	1.779410
42	1	0	-5.718228	3.653883	1.932508
43	1	0	0.007576	-3.100384	-0.017214
44	1	0	3.030512	-0.021173	-0.145322
45	1	0	5.550288	-2.961749	1.701896
46	1	0	3.535279	-4.339467	2.256732
47	1	0	1.293080	-3.555413	1.606598
48	1	0	5.531485	2.589586	-1.378041
49	1	0	7.523579	4.003720	-1.922005
50	1	0	9.787730	3.256018	-1.214278
51	1	0	10.062084	1.119880	0.022990
52	1	0	8.066206	-0.276937	0.556857
53	1	0	-2.180869	3.985702	1.244881
54	1	0	-3.814293	4.692524	1.098612
55	1	0	-3.437520	3.576006	2.445913

Visualization of calculated geometry of (**5a*E***):

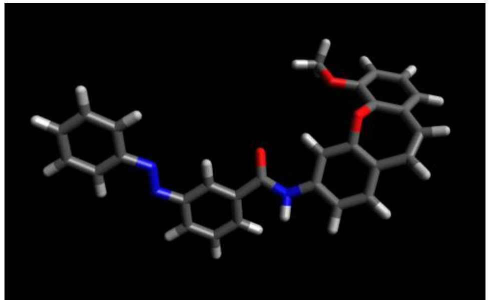

• The calculated coordinates of (**5a*Z***) (the part of calculated log file) -

Standard orientation:

Center Number	Atomic Number	Atomic Type	Coordinates (Angstroms) X	Y	Z
1	6	0	-1.482415	-1.894340	0.090300
2	6	0	-2.114775	-3.046928	0.587593
3	6	0	-3.498589	-3.143181	0.583064
4	6	0	-4.314593	-2.106075	0.094920
5	6	0	-3.652499	-0.973112	-0.413587
6	6	0	-2.267823	-0.846288	-0.413469
7	6	0	-5.769473	-2.233948	0.127625
8	6	0	-6.680153	-1.237031	0.177727
9	6	0	-6.416833	0.202249	0.238628
10	6	0	-5.249799	0.771903	-0.288938
11	8	0	-4.372867	0.022001	-1.063003
12	6	0	-7.344712	1.052133	0.874441
13	6	0	-7.091297	2.411842	0.994871
14	6	0	-5.912671	2.967821	0.487024
15	6	0	-4.979551	2.148178	-0.159054
16	7	0	-0.074818	-1.862324	0.128391
17	6	0	0.779462	-0.908867	-0.377521
18	8	0	0.403260	0.086666	-0.991989
19	6	0	2.241939	-1.172147	-0.143100
20	6	0	3.139686	-0.610402	-1.057217
21	6	0	4.516611	-0.775417	-0.890527
22	6	0	5.007450	-1.553222	0.169805
23	6	0	4.112726	-2.122234	1.072634
24	6	0	2.737761	-1.924979	0.931665
25	7	0	5.357874	-0.294667	-1.952685
26	7	0	6.426910	0.328647	-1.770142
27	6	0	6.859550	0.826856	-0.495383
28	6	0	6.031544	1.577029	0.353473
29	6	0	6.570101	2.156235	1.501013
30	6	0	7.919663	1.977688	1.818062
31	6	0	8.744464	1.241317	0.963183
32	6	0	8.224700	0.692040	-0.207050
33	8	0	-3.809978	2.580999	-0.705856
34	6	0	-3.486422	3.961075	-0.579762
35	1	0	-1.517086	-3.869273	0.974375
36	1	0	-3.969480	-4.042381	0.972406

37	1	0	-1.816021	0.044990	-0.822234
38	1	0	-6.142009	-3.254948	0.194786
39	1	0	-7.726169	-1.519505	0.282208
40	1	0	-8.257672	0.625157	1.280678
41	1	0	-7.812482	3.057591	1.488133
42	1	0	-5.735528	4.032463	0.586266
43	1	0	0.355885	-2.702668	0.492256
44	1	0	2.759687	-0.038164	-1.896994
45	1	0	6.074604	-1.715965	0.278765
46	1	0	4.489367	-2.716775	1.899963
47	1	0	2.067018	-2.344409	1.676709
48	1	0	4.985533	1.723765	0.105644
49	1	0	5.931577	2.748383	2.150908
50	1	0	8.330035	2.424995	2.719081
51	1	0	9.797486	1.111893	1.197398
52	1	0	8.858813	0.146808	-0.900553
53	1	0	-2.514715	4.082444	-1.061297
54	1	0	-4.224838	4.595669	-1.086127
55	1	0	-3.411375	4.261839	0.472916

Visualization of calculated geometry of (**5a*Z***):

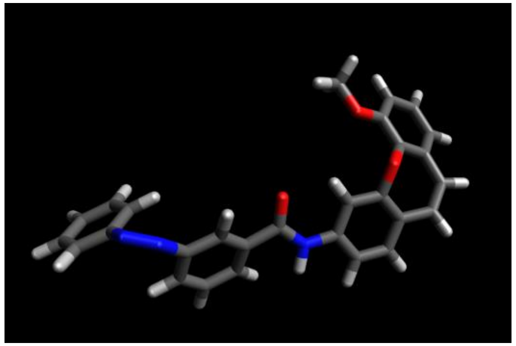

- The calculated coordinates of (**5b*E***) (the part of calculated log file) -

Standard orientation:

Center Number	Atomic Number	Atomic Type	Coordinates (Angstroms) X	Y	Z
1	6	0	-2.060029	-1.956618	-0.434540
2	6	0	-2.878276	-3.059052	-0.736146
3	6	0	-4.203344	-2.868894	-1.100068
4	6	0	-4.776927	-1.586240	-1.174859
5	6	0	-3.927222	-0.501445	-0.889395
6	6	0	-2.598176	-0.663118	-0.514433
7	6	0	-6.184165	-1.425473	-1.532499

8	6	0	-7.006089	-0.408329	-1.192144
9	6	0	-6.680893	0.755065	-0.364098
10	6	0	-5.377883	1.262420	-0.267362
11	8	0	-4.366025	0.800314	-1.099733
12	6	0	-7.695654	1.377241	0.391501
13	6	0	-7.400562	2.445878	1.227292
14	6	0	-6.092684	2.929718	1.336405
15	6	0	-5.068237	2.337452	0.588493
16	7	0	-0.728086	-2.222861	-0.062540
17	6	0	0.285689	-1.332557	0.212682
18	8	0	0.165109	-0.113245	0.119738
19	6	0	1.588598	-1.958183	0.632497
20	6	0	2.751220	-1.224437	0.392216
21	6	0	3.999770	-1.739113	0.767581
22	6	0	4.077344	-2.986560	1.403101
23	6	0	2.916520	-3.711740	1.663587
24	6	0	1.675523	-3.203413	1.281076
25	7	0	5.241556	-1.081420	0.561307
26	7	0	5.157131	0.033774	-0.022070
27	6	0	6.389998	0.697182	-0.226059
28	6	0	6.305185	1.940842	-0.869851
29	6	0	7.452318	2.688229	-1.123716
30	6	0	8.677992	2.168280	-0.723034
31	6	0	8.795664	0.934043	-0.081450
32	6	0	7.646008	0.197283	0.167209
33	9	0	9.797463	2.880634	-0.961244
34	8	0	-3.765613	2.733748	0.600663
35	6	0	-3.404554	3.808744	1.460399
36	1	0	-2.470709	-4.066284	-0.686105
37	1	0	-4.820972	-3.734172	-1.327469
38	1	0	-1.993280	0.207839	-0.311704
39	1	0	-6.624141	-2.265527	-2.067774
40	1	0	-8.053521	-0.494671	-1.475499
41	1	0	-8.712190	0.999860	0.320052
42	1	0	-8.189978	2.916919	1.806417
43	1	0	-5.883516	3.768136	1.990725
44	1	0	-0.471910	-3.201649	-0.083229
45	1	0	2.688523	-0.254306	-0.085340
46	1	0	5.054931	-3.362733	1.689840
47	1	0	2.975080	-4.669366	2.172400
48	1	0	0.778290	-3.766537	1.524560
49	1	0	5.326150	2.306517	-1.164248
50	1	0	7.409357	3.652416	-1.619805
51	1	0	9.777614	0.574397	0.210169
52	1	0	7.698607	-0.765081	0.663882

53	1	0	-2.330841	3.948358	1.324635
54	1	0	-3.928870	4.734025	1.189642
55	1	0	-3.610568	3.569938	2.511516

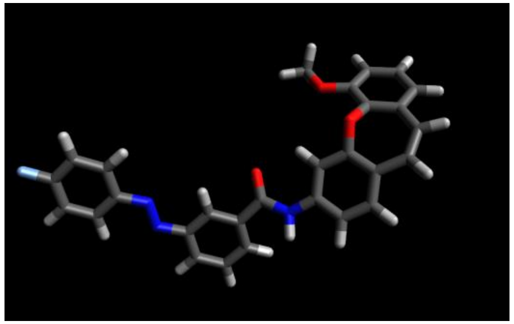

Visualization of calculated geometry of (**5b*E***):

- The calculated coordinates of (**5b*Z***) (the part of calculated log file) -

Standard orientation:

Center Number	Atomic Number	Atomic Type	Coordinates (Angstroms) X	Y	Z
1	6	0	-1.814962	-1.938841	0.198801
2	6	0	-2.472338	-3.018625	0.813061
3	6	0	-3.858048	-3.058016	0.861942
4	6	0	-4.651138	-2.033521	0.313312
5	6	0	-3.965477	-0.976730	-0.313874
6	6	0	-2.577760	-0.907473	-0.369891
7	6	0	-6.107500	-2.097095	0.408565
8	6	0	-6.976084	-1.062269	0.402900
9	6	0	-6.656328	0.364767	0.324149
10	6	0	-5.490723	0.837966	-0.293929
11	8	0	-4.668555	-0.011169	-1.023642
12	6	0	-7.528327	1.304576	0.910713
13	6	0	-7.223236	2.658910	0.895289
14	6	0	-6.046821	3.120200	0.295790
15	6	0	-5.168838	2.209467	-0.303700
16	7	0	-0.406616	-1.958777	0.193696
17	6	0	0.466081	-1.086823	-0.416419
18	8	0	0.108423	-0.138658	-1.111651
19	6	0	1.924288	-1.377505	-0.186445
20	6	0	2.820149	-0.936065	-1.166462
21	6	0	4.194118	-1.130023	-1.007623
22	6	0	4.681412	-1.817643	0.115148
23	6	0	3.787995	-2.267528	1.083872
24	6	0	2.417198	-2.040088	0.947404
25	7	0	5.037105	-0.788649	-2.120429

```
    26          7           0        6.114580   -0.160461   -2.015626
    27          6           0        6.560367    0.480873   -0.813970
    28          6           0        5.740435    1.284305   -0.004555
    29          6           0        6.291151    1.987536    1.062804
    30          6           0        7.653767    1.863077    1.318816
    31          6           0        8.491846    1.088786    0.522624
    32          6           0        7.940345    0.421821   -0.567326
    33          9           0        8.182209    2.532455    2.363415
    34          8           0       -4.007689    2.545272   -0.930654
    35          6           0       -3.640613    3.919850   -0.961468
    36          1           0       -1.892819   -3.828633    1.250222
    37          1           0       -4.348497   -3.900625    1.342913
    38          1           0       -2.108143   -0.073205   -0.868984
    39          1           0       -6.516376   -3.091659    0.580032
    40          1           0       -8.027369   -1.290236    0.569211
    41          1           0       -8.439524    0.951774    1.386189
    42          1           0       -7.901569    3.374335    1.351821
    43          1           0       -5.828543    4.181835    0.288901
    44          1           0        0.004767   -2.778410    0.621580
    45          1           0        2.440645   -0.434196   -2.050327
    46          1           0        5.745369   -2.003960    0.220578
    47          1           0        4.162712   -2.791538    1.958412
    48          1           0        1.748187   -2.363884    1.740064
    49          1           0        4.681955    1.379691   -0.219268
    50          1           0        5.680764    2.626317    1.693246
    51          1           0        9.552544    1.034892    0.745687
    52          1           0        8.569201   -0.163232   -1.231951
    53          1           0       -2.689736    3.960310   -1.495344
    54          1           0       -4.384655    4.523095   -1.497066
    55          1           0       -3.507482    4.323544    0.050283
---------------------------------------------------------------------
```

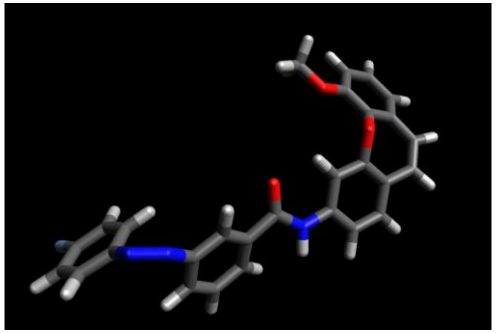

Visualization of calculated geometry of (**5b*Z***):

- The calculated coordinates of (**5c*E***) (the part of calculated log file) -

```
                Standard orientation:
 ---------------------------------------------------------------------
 Center     Atomic     Atomic              Coordinates (Angstroms)
```

Number	Number	Type	X	Y	Z
1	6	0	-2.438257	-1.848761	-0.372983
2	6	0	-3.173319	-3.034142	-0.547009
3	6	0	-4.523678	-2.981040	-0.860619
4	6	0	-5.205129	-1.758967	-1.008481
5	6	0	-4.437925	-0.589943	-0.850198
6	6	0	-3.085507	-0.613530	-0.528666
7	6	0	-6.634852	-1.740891	-1.308454
8	6	0	-7.523711	-0.768888	-1.006300
9	6	0	-7.261359	0.478966	-0.286066
10	6	0	-6.002563	1.095473	-0.293684
11	8	0	-4.993389	0.650847	-1.138889
12	6	0	-8.290833	1.075837	0.469950
13	6	0	-8.050308	2.227999	1.206346
14	6	0	-6.784144	2.822431	1.212780
15	6	0	-5.747248	2.256875	0.461345
16	7	0	-1.074527	-1.975048	-0.044153
17	6	0	-0.125901	-0.986423	0.091329
18	8	0	-0.347590	0.204482	-0.115446
19	6	0	1.238793	-1.462463	0.506625
20	6	0	2.332063	-0.671353	0.135446
21	6	0	3.631819	-1.036313	0.495735
22	6	0	3.848180	-2.200341	1.256128
23	6	0	2.762298	-2.974928	1.644443
24	6	0	1.461576	-2.613683	1.274426
25	7	0	4.654266	-0.160354	0.047679
26	7	0	5.821751	-0.506690	0.380016
27	6	0	6.840142	0.361650	-0.062581
28	6	0	8.157672	0.020591	0.289568
29	6	0	9.250315	0.791699	-0.084719
30	6	0	9.001657	1.934861	-0.833853
31	6	0	7.714762	2.321897	-1.212113
32	6	0	6.643996	1.530858	-0.822553
33	9	0	8.379731	-1.086899	1.013464
34	9	0	10.044894	2.696394	-1.207724
35	8	0	-4.483375	2.756685	0.377878
36	6	0	-4.176736	3.921825	1.135127
37	1	0	-2.681366	-3.998048	-0.437843
38	1	0	-5.075554	-3.908857	-0.988885
39	1	0	-2.548074	0.316992	-0.424831
40	1	0	-7.025218	-2.653666	-1.755938
41	1	0	-8.571466	-0.961049	-1.230219
42	1	0	-9.274284	0.613645	0.478195
43	1	0	-8.850357	2.678729	1.787034

44	1	0	-6.617846	3.724081	1.790832
45	1	0	-0.740312	-2.928563	0.014766
46	1	0	2.174021	0.235079	-0.438785
47	1	0	4.861444	-2.469195	1.533049
48	1	0	2.919476	-3.865314	2.246691
49	1	0	0.629363	-3.220461	1.621735
50	1	0	10.257352	0.508153	0.199447
51	1	0	7.575370	3.224467	-1.797944
52	1	0	5.628939	1.796351	-1.095975
53	1	0	-3.125425	4.135980	0.935920
54	1	0	-4.788022	4.777748	0.821937
55	1	0	-4.316070	3.751213	2.210203

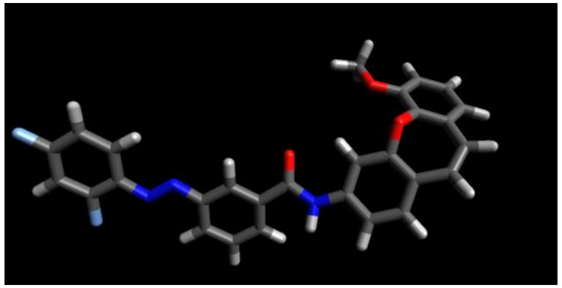

Visualization of calculated geometry of (**5c*E***):

- The calculated coordinates of (**5c*Z***) (the part of calculated log file) -

Standard orientation:

Center Number	Atomic Number	Atomic Type	Coordinates (Angstroms) X	Y	Z
1	6	0	0.786076	-0.343098	-1.215296
2	6	0	0.987384	-1.370603	-2.152842
3	6	0	2.149985	-2.127561	-2.121639
4	6	0	3.160440	-1.900699	-1.168946
5	6	0	2.921237	-0.878204	-0.232194
6	6	0	1.770910	-0.097559	-0.246336
7	6	0	4.384267	-2.698621	-1.184505
8	6	0	5.604864	-2.335487	-0.732598
9	6	0	5.985632	-1.049082	-0.145220
10	6	0	5.066544	-0.235058	0.531005
11	8	0	3.791390	-0.695186	0.835510
12	6	0	7.310838	-0.587203	-0.280342
13	6	0	7.682038	0.653217	0.220815
14	6	0	6.751712	1.469841	0.872150
15	6	0	5.432083	1.030329	1.030179

16	7	0	-0.431604	0.363229	-1.278976
17	6	0	-0.839268	1.440635	-0.525563
18	8	0	-0.127687	2.010683	0.297416
19	6	0	-2.252210	1.892650	-0.772756
20	6	0	-3.276995	1.009591	-1.129075
21	6	0	-4.577757	1.495302	-1.310677
22	6	0	-4.850929	2.861463	-1.187332
23	6	0	-3.835446	3.730095	-0.788507
24	6	0	-2.545337	3.249398	-0.572632
25	7	0	-5.625737	0.643257	-1.796974
26	7	0	-5.935894	-0.424338	-1.230588
27	6	0	-5.406458	-0.784317	0.066678
28	6	0	-4.601573	-1.916354	0.195126
29	6	0	-4.216510	-2.421403	1.427145
30	6	0	-4.693606	-1.770805	2.561542
31	6	0	-5.506091	-0.645100	2.487584
32	6	0	-5.861667	-0.155428	1.230783
33	9	0	-4.127352	-2.488179	-0.933840
34	9	0	-4.342489	-2.249521	3.770925
35	8	0	4.448732	1.729477	1.661372
36	6	0	4.770027	3.018387	2.171666
37	1	0	0.227607	-1.577646	-2.903020
38	1	0	2.288731	-2.917078	-2.855992
39	1	0	1.648323	0.678318	0.494389
40	1	0	4.304046	-3.659413	-1.690540
41	1	0	6.428127	-3.026860	-0.902928
42	1	0	8.036553	-1.212146	-0.793734
43	1	0	8.705447	1.000278	0.108934
44	1	0	7.061853	2.433047	1.260145
45	1	0	-1.043544	0.089180	-2.038195
46	1	0	-3.086506	-0.050273	-1.265511
47	1	0	-5.856663	3.223844	-1.379849
48	1	0	-4.050417	4.786564	-0.657483
49	1	0	-1.745954	3.915038	-0.265044
50	1	0	-3.566168	-3.286095	1.499912
51	1	0	-5.855085	-0.168717	3.397523
52	1	0	-6.510728	0.711050	1.148607
53	1	0	3.847346	3.400933	2.611051
54	1	0	5.546017	2.962881	2.945763
55	1	0	5.101622	3.695597	1.374310

Visualization of calculated geometry of (**5c*Z***) :

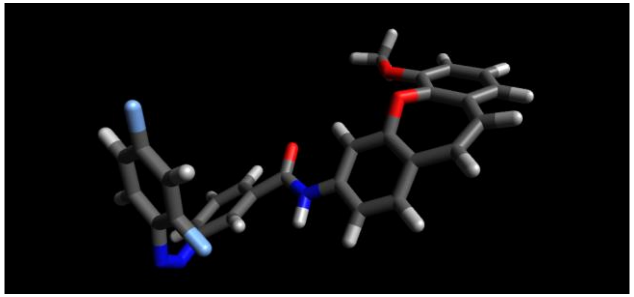

• The calculated coordinates of (**5d*E***) (the part of calculated log file) -

Standard orientation:

Center Number	Atomic Number	Atomic Type	Coordinates (Angstroms) X	Y	Z
1	6	0	1.583502	-0.802477	-0.191246
2	6	0	1.606251	-2.132160	-0.646508
3	6	0	2.772000	-2.879953	-0.567728
4	6	0	3.962486	-2.347164	-0.040032
5	6	0	3.902147	-1.022315	0.430591
6	6	0	2.751232	-0.246124	0.353078
7	6	0	5.180311	-3.153191	-0.001697
8	6	0	6.457301	-2.711146	-0.013881
9	6	0	6.913811	-1.321935	-0.093237
10	6	0	6.139808	-0.253115	0.379237
11	8	0	4.982575	-0.477804	1.114507
12	6	0	8.157954	-1.030969	-0.689297
13	6	0	8.586617	0.282474	-0.825810
14	6	0	7.796312	1.344301	-0.373564
15	6	0	6.562185	1.082689	0.233115
16	7	0	0.362298	-0.107319	-0.285644
17	6	0	0.086249	1.199555	0.047608
18	8	0	0.933422	1.995741	0.445879
19	6	0	-1.349974	1.612196	-0.119562
20	6	0	-2.426492	0.725582	-0.003682
21	6	0	-3.743048	1.183776	-0.137333
22	6	0	-3.990239	2.543081	-0.394076
23	6	0	-2.920523	3.426090	-0.498872
24	6	0	-1.608423	2.969886	-0.352783
25	7	0	-4.743236	0.187335	0.018023
26	7	0	-5.921235	0.625163	-0.115949
27	6	0	-6.961702	-0.306401	0.025702
28	6	0	-6.882536	-1.694831	0.283900
29	6	0	-8.011392	-2.493719	0.396449
30	6	0	-9.275752	-1.919180	0.252676
31	6	0	-9.412452	-0.554617	-0.003145
32	6	0	-8.266011	0.217317	-0.111412
33	9	0	-5.686699	-2.282087	0.426748

34	9	0	-8.398701	1.530020	-0.357896
35	8	0	5.719790	2.031938	0.726416
36	6	0	6.101209	3.396157	0.590715
37	1	0	0.705151	-2.578953	-1.060614
38	1	0	2.770122	-3.905490	-0.928476
39	1	0	2.769009	0.768699	0.721179
40	1	0	5.023984	-4.229890	-0.047198
41	1	0	7.245713	-3.459705	-0.067622
42	1	0	8.772755	-1.850188	-1.052309
43	1	0	9.546263	0.495603	-1.288512
44	1	0	8.150868	2.362402	-0.485479
45	1	0	-0.386436	-0.624116	-0.729298
46	1	0	-2.286525	-0.327009	0.225820
47	1	0	-5.015538	2.880655	-0.497243
48	1	0	-3.105780	4.479197	-0.691671
49	1	0	-0.770316	3.655936	-0.417230
50	1	0	-7.884087	-3.552856	0.594847
51	1	0	-10.160862	-2.541678	0.340946
52	1	0	-10.383083	-0.084612	-0.119056
53	1	0	5.289894	3.975654	1.034250
54	1	0	7.035123	3.608076	1.126716
55	1	0	6.216277	3.678798	-0.463398

Visualization of calculated geometry of (**5d*E***) :

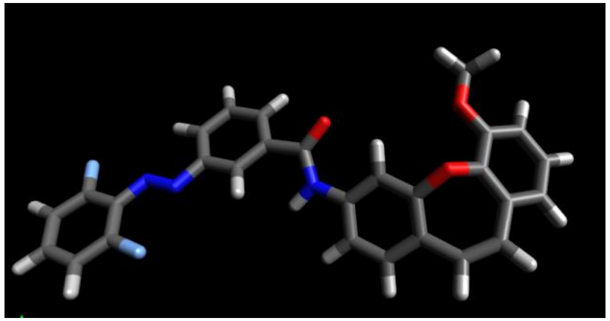

- The calculated coordinates of (**5d*Z***) (the part of calculated log file) -

Standard orientation:

Center Number	Atomic Number	Atomic Type	Coordinates (Angstroms) X	Y	Z
1	6	0	-1.236362	-2.159776	0.176027
2	6	0	-1.821121	-3.110252	1.030498
3	6	0	-3.193825	-3.120736	1.230471
4	6	0	-4.043919	-2.193976	0.599463
5	6	0	-3.431576	-1.268855	-0.266267

6	6	0	-2.057704	-1.228426	-0.476896
7	6	0	-5.482543	-2.219267	0.852737
8	6	0	-6.349682	-1.186677	0.763981
9	6	0	-6.040821	0.202883	0.419689
10	6	0	-4.945647	0.547198	-0.384408
11	8	0	-4.211477	-0.426980	-1.049680
12	6	0	-6.843397	1.242013	0.933186
13	6	0	-6.535817	2.569730	0.668051
14	6	0	-5.425912	2.904959	-0.114362
15	6	0	-4.619097	1.892154	-0.646355
16	7	0	0.161843	-2.213338	0.011249
17	6	0	0.968165	-1.428428	-0.780193
18	8	0	0.559216	-0.487560	-1.455842
19	6	0	2.429519	-1.788389	-0.754935
20	6	0	3.336815	-0.755323	-1.015366
21	6	0	4.708559	-1.020285	-1.037361
22	6	0	5.179664	-2.327767	-0.854679
23	6	0	4.277441	-3.351910	-0.584640
24	6	0	2.906527	-3.088426	-0.534905
25	7	0	5.665809	-0.029331	-1.423958
26	7	0	5.706147	1.118531	-0.930740
27	6	0	4.941761	1.499907	0.223694
28	6	0	4.273029	2.730843	0.206294
29	6	0	3.667321	3.271755	1.329760
30	6	0	3.768457	2.583105	2.541140
31	6	0	4.457119	1.371405	2.616630
32	6	0	5.031544	0.853654	1.464245
33	9	0	4.189407	3.377223	-0.972236
34	9	0	5.726412	-0.298783	1.536908
35	8	0	-3.527520	2.098896	-1.433474
36	6	0	-3.145829	3.443023	-1.702815
37	1	0	-1.196270	-3.844489	1.534001
38	1	0	-3.627682	-3.862360	1.896512
39	1	0	-1.643958	-0.490159	-1.147351
40	1	0	-5.869274	-3.165662	1.227656
41	1	0	-7.377600	-1.368262	1.072613

42	1	0	-7.700710	0.989239	1.551202
43	1	0	-7.159902	3.362985	1.070157
44	1	0	-5.205031	3.947204	-0.313610
45	1	0	0.631534	-2.885193	0.604666
46	1	0	2.953888	0.236412	-1.226881
47	1	0	6.246281	-2.521362	-0.918376
48	1	0	4.637973	-4.365371	-0.435557
49	1	0	2.217685	-3.913627	-0.375361
50	1	0	3.136334	4.214188	1.247283
51	1	0	3.308551	2.995619	3.433595
52	1	0	4.561839	0.827965	3.549872
53	1	0	-2.248525	3.377711	-2.320299
54	1	0	-3.928163	3.980911	-2.253374
55	1	0	-2.913712	3.987606	-0.778789

Visualization of calculated geometry of (**5dZ**) :

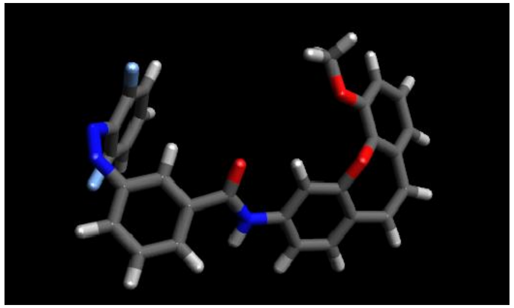

- The calculated coordinates of (**5e*E***) (the part of calculated log file) -

Standard orientation:

Center Number	Atomic Number	Atomic Type	Coordinates (Angstroms) X	Y	Z
1	6	0	1.417253	-2.376617	-0.165443
2	6	0	2.156665	-3.387892	-0.800760
3	6	0	3.542024	-3.323528	-0.833195
4	6	0	4.257298	-2.262979	-0.245966
5	6	0	3.490641	-1.276694	0.402429
6	6	0	2.100786	-1.312354	0.441535
7	6	0	5.714463	-2.227505	-0.338541
8	6	0	6.513435	-1.139370	-0.286701
9	6	0	6.111356	0.254430	-0.118274
10	6	0	4.908142	0.648893	0.506184
11	8	0	4.093468	-0.273003	1.154041

12	6	0	6.937926	1.285740	-0.592498
13	6	0	6.599223	2.633945	-0.487472
14	6	0	5.388396	2.987265	0.121461
15	6	0	4.545233	1.981486	0.619513
16	7	0	0.014838	-2.506788	-0.151564
17	6	0	-0.926821	-1.635098	0.346987
18	8	0	-0.651325	-0.542475	0.838250
19	6	0	-2.352233	-2.104555	0.239246
20	6	0	-3.340888	-1.122063	0.168052
21	6	0	-4.691349	-1.486500	0.079350
22	6	0	-5.049334	-2.842058	0.084713
23	6	0	-4.065823	-3.824800	0.176785
24	6	0	-2.721434	-3.461771	0.252657
25	7	0	-5.772892	-0.569256	-0.009506
26	7	0	-5.431725	0.644080	-0.047817
27	6	0	-6.504538	1.566852	-0.130969
28	6	0	-6.130002	2.917311	-0.174919
29	6	0	-7.102964	3.912397	-0.257166
30	6	0	-8.453751	3.560983	-0.296028
31	6	0	-8.830349	2.211422	-0.252411
32	6	0	-7.865754	1.214225	-0.170204
33	8	0	4.944841	4.262964	0.292305
34	6	0	5.762192	5.329811	-0.180045
35	1	0	1.642943	-4.226211	-1.265849
36	1	0	4.097431	-4.113986	-1.331733
37	1	0	1.561011	-0.531196	0.955332
38	1	0	6.185984	-3.184631	-0.554686
39	1	0	7.576605	-1.292970	-0.464278
40	1	0	7.879135	1.017919	-1.066821
41	1	0	7.277237	3.386177	-0.873058
42	1	0	3.621542	2.250503	1.120499
43	1	0	-0.339750	-3.313417	-0.649552
44	1	0	-3.064757	-0.074793	0.181048
45	1	0	-6.102291	-3.100607	0.022281
46	1	0	-4.344645	-4.874176	0.198458
47	1	0	-1.970024	-4.239884	0.359234
48	1	0	-5.072074	3.161375	-0.143088
49	1	0	-6.808568	4.957624	-0.290817
50	1	0	-9.215177	4.333724	-0.359992
51	1	0	-9.882950	1.942423	-0.282905
52	1	0	-8.139747	0.165488	-0.135393
53	1	0	5.221646	6.247013	0.059223
54	1	0	6.737293	5.339535	0.322583
55	1	0	5.912969	5.268071	-1.264962

Visualization of calculated geometry of ((**5e*E***) :

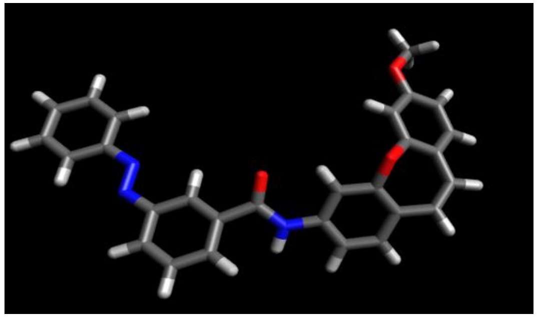

- The calculated coordinates of (**5e*Z***) (the part of calculated log file) -

Standard orientation:

Center Number	Atomic Number	Atomic Type	Coordinates (Angstroms) X	Y	Z
1	6	0	1.179330	-2.076518	-0.158693
2	6	0	1.754735	-3.209391	-0.757565
3	6	0	3.132650	-3.369557	-0.767276
4	6	0	4.001862	-2.421592	-0.195024
5	6	0	3.395525	-1.307265	0.414219
6	6	0	2.017517	-1.119968	0.433156
7	6	0	5.446761	-2.624163	-0.261704
8	6	0	6.409990	-1.677623	-0.222864
9	6	0	6.235278	-0.233213	-0.097588
10	6	0	5.102632	0.367390	0.493386
11	8	0	4.140975	-0.392078	1.150140
12	6	0	7.223565	0.637953	-0.583423
13	6	0	7.104425	2.025470	-0.520753
14	6	0	5.956962	2.586407	0.054183
15	6	0	4.956150	1.743657	0.563336
16	7	0	-0.225223	-1.970607	-0.184540
17	6	0	-1.027096	-0.993649	0.359305
18	8	0	-0.600287	-0.050390	1.022397
19	6	0	-2.500456	-1.162360	0.109120
20	6	0	-3.371277	-0.603104	1.050027
21	6	0	-4.754109	-0.676220	0.867284
22	6	0	-5.280674	-1.361606	-0.238791
23	6	0	-4.413247	-1.931541	-1.167223
24	6	0	-3.030206	-1.823807	-1.008545
25	7	0	-5.573294	-0.208412	1.951858
26	7	0	-6.607646	0.478492	1.800861
27	6	0	-7.002067	1.076356	0.557077

28	6	0	-6.126044	1.828651	-0.241028
29	6	0	-6.621476	2.505543	-1.353628
30	6	0	-7.976249	2.422170	-1.687886
31	6	0	-8.848062	1.682840	-0.883796
32	6	0	-8.369952	1.036516	0.254300
33	8	0	5.722099	3.921482	0.180692
34	6	0	6.710625	4.827833	-0.299804
35	1	0	1.119073	-3.964574	-1.214408
36	1	0	3.559770	-4.252925	-1.235322
37	1	0	1.605742	-0.253832	0.928730
38	1	0	5.760491	-3.650427	-0.444842
39	1	0	7.436918	-2.004983	-0.377027
40	1	0	8.116453	0.209257	-1.032368
41	1	0	7.900445	2.647465	-0.912728
42	1	0	4.079671	2.171003	1.038535
43	1	0	-0.701102	-2.763770	-0.595241
44	1	0	-2.966539	-0.102571	1.923456
45	1	0	-6.354541	-1.454420	-0.363260
46	1	0	-4.817232	-2.454661	-2.029221
47	1	0	-2.378167	-2.240751	-1.771421
48	1	0	-5.075669	1.901463	0.020701
49	1	0	-5.945643	3.099282	-1.963146
50	1	0	-8.353247	2.943831	-2.563212
51	1	0	-9.904817	1.628248	-1.130422
52	1	0	-9.039626	0.487206	0.910205
53	1	0	6.320795	5.826924	-0.098492
54	1	0	7.664101	4.695495	0.226223
55	1	0	6.871413	4.709075	-1.378648

Visualization of calculated geometry of (**5e*Z***) :

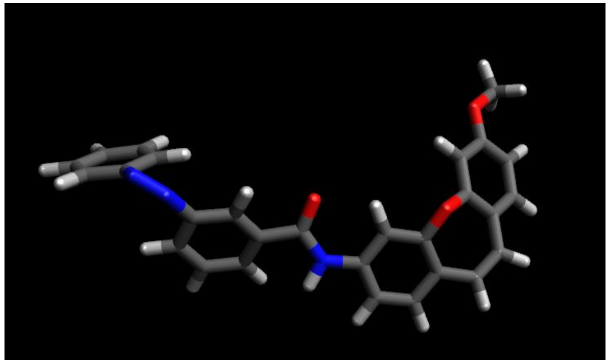

- The calculated coordinates of (**5f*E***) (the part of calculated log file) -

```
                         Standard orientation:
---------------------------------------------------------------------
 Center     Atomic      Atomic             Coordinates (Angstroms)
```

Number	Number	Type	X	Y	Z
1	6	0	-1.738954	-2.179846	-0.330990
2	6	0	-2.492453	-3.340949	-0.569983
3	6	0	-3.813018	-3.242478	-0.983194
4	6	0	-4.449336	-2.001255	-1.172194
5	6	0	-3.663986	-0.855978	-0.942316
6	6	0	-2.340062	-0.926706	-0.521809
7	6	0	-5.851020	-1.956179	-1.580140
8	6	0	-6.737836	-0.961122	-1.360056
9	6	0	-6.504158	0.304112	-0.669303
10	6	0	-5.233817	0.910076	-0.559483
11	8	0	-4.134300	0.416045	-1.252635
12	6	0	-7.575295	0.981776	-0.065075
13	6	0	-7.414905	2.177372	0.633579
14	6	0	-6.136331	2.739412	0.738882
15	6	0	-5.045684	2.095118	0.133829
16	7	0	-0.404662	-2.345182	0.089117
17	6	0	0.549005	-1.382648	0.331995
18	8	0	0.360658	-0.179132	0.166766
19	6	0	1.876935	-1.906155	0.807917
20	6	0	3.002699	-1.131834	0.523824
21	6	0	4.272032	-1.553732	0.942675
22	6	0	4.406229	-2.746278	1.667682
23	6	0	3.281217	-3.510068	1.971959
24	6	0	2.019983	-3.095966	1.544478
25	7	0	5.480748	-0.850366	0.693843
26	7	0	5.344730	0.207700	0.020782
27	6	0	6.542845	0.916710	-0.230320
28	6	0	6.398706	2.095410	-0.977426
29	6	0	7.507312	2.878089	-1.289274
30	6	0	8.754974	2.459135	-0.840957
31	6	0	8.931382	1.291822	-0.095968
32	6	0	7.819696	0.518970	0.209708
33	9	0	9.837788	3.206417	-1.134900
34	8	0	-5.846799	3.901508	1.386184
35	6	0	-6.915682	4.613739	2.002512
36	1	0	-2.039400	-4.320440	-0.434626
37	1	0	-4.380798	-4.151968	-1.163038
38	1	0	-1.776560	-0.017304	-0.376915
39	1	0	-6.226292	-2.868240	-2.041023
40	1	0	-7.769718	-1.138280	-1.658863
41	1	0	-8.570355	0.549982	-0.141950
42	1	0	-8.279327	2.654707	1.079753
43	1	0	-4.058353	2.541434	0.184741

44	1	0	-0.092767	-3.306853	0.135775
45	1	0	2.896019	-0.203160	-0.023296
46	1	0	5.398718	-3.050375	1.986947
47	1	0	3.383411	-4.424307	2.549090
48	1	0	1.149586	-3.686241	1.819524
49	1	0	5.404504	2.382380	-1.306230
50	1	0	7.418312	3.793110	-1.865808
51	1	0	9.928226	1.010653	0.229431
52	1	0	7.918034	-0.393979	0.786367
53	1	0	-6.462649	5.497760	2.454123
54	1	0	-7.666384	4.924705	1.265415
55	1	0	-7.399345	4.012940	2.782658

Visualization of calculated geometry of (**5f*E***) 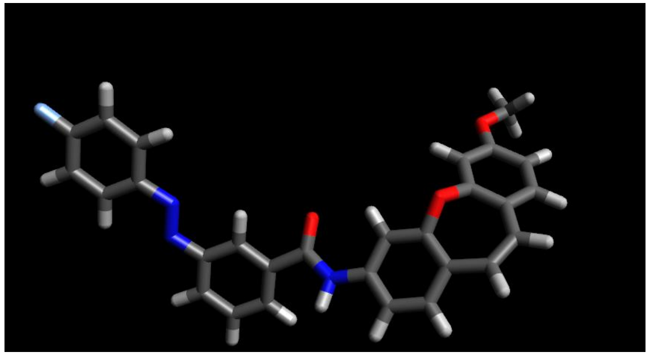 :

- The calculated coordinates of (**5f*Z***) (the part of calculated log file) -

Standard orientation:

Center Number	Atomic Number	Atomic Type	Coordinates (Angstroms) X	Y	Z
1	6	0	1.521284	-2.156806	-0.250756
2	6	0	2.127713	-3.207458	-0.958784
3	6	0	3.510443	-3.296776	-1.025206
4	6	0	4.353119	-2.355627	-0.404500
5	6	0	3.716846	-1.327395	0.316049
6	6	0	2.333043	-1.211202	0.393350
7	6	0	5.802750	-2.476350	-0.536279
8	6	0	6.718319	-1.486405	-0.452637
9	6	0	6.476938	-0.067962	-0.202483
10	6	0	5.341174	0.421103	0.478302
11	8	0	4.445946	-0.440658	1.102092
12	6	0	7.400088	0.891228	-0.649451
13	6	0	7.215401	2.260594	-0.464992
14	6	0	6.067049	2.710435	0.199168
15	6	0	5.130699	1.777183	0.671113
16	7	0	0.113538	-2.114827	-0.228208
17	6	0	-0.710990	-1.222558	0.416935
18	8	0	-0.302759	-0.322767	1.148871
19	6	0	-2.182939	-1.422848	0.179416
20	6	0	-3.053014	-0.961802	1.173399

21	6	0	-4.435684	-1.066070	1.006755
22	6	0	-4.960975	-1.686178	-0.137811
23	6	0	-4.093712	-2.157402	-1.120162
24	6	0	-2.712224	-2.016022	-0.976229
25	7	0	-5.258360	-0.704641	2.128416
26	7	0	-6.287486	0.002197	2.040796
27	6	0	-6.677980	0.710799	0.857064
28	6	0	-5.796132	1.486603	0.086722
29	6	0	-6.285343	2.260703	-0.961618
30	6	0	-7.649381	2.233848	-1.237830
31	6	0	-8.546669	1.488225	-0.479703
32	6	0	-8.055007	0.749259	0.592419
33	9	0	-8.119353	2.972375	-2.263803
34	8	0	5.772626	4.015625	0.449913
35	6	0	6.695371	5.010458	0.016195
36	1	0	1.512720	-3.954142	-1.456001
37	1	0	3.962198	-4.115391	-1.579839
38	1	0	1.894030	-0.412890	0.972730
39	1	0	6.159955	-3.466574	-0.813649
40	1	0	7.753594	-1.745669	-0.668135
41	1	0	8.293392	0.548356	-1.166143
42	1	0	7.962185	2.954130	-0.832938
43	1	0	4.254833	2.117333	1.213070
44	1	0	-0.339955	-2.889864	-0.694801
45	1	0	-2.646663	-0.511811	2.073139
46	1	0	-6.033908	-1.804279	-0.250358
47	1	0	-4.497140	-2.628469	-2.011862
48	1	0	-2.062426	-2.351101	-1.780094
49	1	0	-4.736754	1.504646	0.317256
50	1	0	-5.626145	2.879670	-1.561962
51	1	0	-9.605293	1.510479	-0.717552
52	1	0	-8.729937	0.183323	1.227773
53	1	0	6.265031	5.966798	0.317765
54	1	0	7.674324	4.883912	0.494914
55	1	0	6.818681	4.994008	-1.073795

Visualization of calculated geometry of (**5fZ**) :

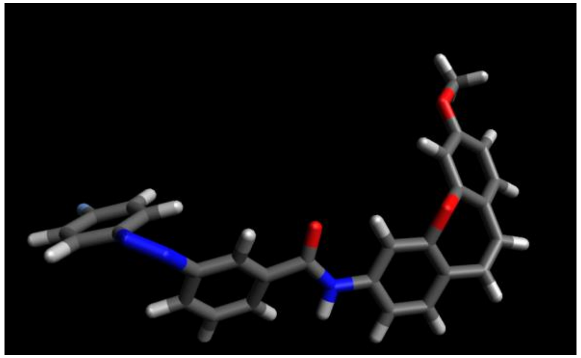

- The calculated coordinates of (**5g*E***) (the part of calculated log file) -

Standard orientation:

Center Number	Atomic Number	Atomic Type	Coordinates (Angstroms) X	Y	Z
1	6	0	-2.167582	-2.070498	-0.255840
2	6	0	-2.862413	-3.290628	-0.296565
3	6	0	-4.211843	-3.314761	-0.617037
4	6	0	-4.935086	-2.141924	-0.904381
5	6	0	-4.207642	-0.937346	-0.875999
6	6	0	-2.856064	-0.884528	-0.551697
7	6	0	-6.362507	-2.220713	-1.203662
8	6	0	-7.290390	-1.253607	-1.033565
9	6	0	-7.084955	0.097132	-0.517672
10	6	0	-5.849748	0.777363	-0.584782
11	8	0	-4.777049	0.258518	-1.301630
12	6	0	-8.147180	0.786545	0.089093
13	6	0	-8.008281	2.064961	0.627494
14	6	0	-6.762498	2.701571	0.559937
15	6	0	-5.683158	2.046315	-0.053505
16	7	0	-0.800762	-2.112306	0.081860
17	6	0	0.102273	-1.076676	0.156531
18	8	0	-0.170098	0.087975	-0.128224
19	6	0	1.484116	-1.464530	0.605396
20	6	0	2.542977	-0.645961	0.195828
21	6	0	3.855922	-0.933393	0.577929
22	6	0	4.119989	-2.044072	1.400475
23	6	0	3.067621	-2.843896	1.827764
24	6	0	1.754228	-2.561324	1.435393
25	7	0	4.841444	-0.038263	0.086556
26	7	0	6.023229	-0.328751	0.421815
27	6	0	7.006248	0.559487	-0.059066
28	6	0	8.338695	0.270649	0.283683
29	6	0	9.400610	1.066563	-0.125234
30	6	0	9.104908	2.181440	-0.899534
31	6	0	7.801218	2.516769	-1.269673
32	6	0	6.761971	1.701737	-0.845698
33	9	0	8.606158	-0.810136	1.032133
34	9	0	10.117676	2.966656	-1.306635
35	8	0	-6.496597	3.947116	1.040652
36	6	0	-7.555031	4.669689	1.662824
37	1	0	-2.341622	-4.220232	-0.078374
38	1	0	-4.733287	-4.268335	-0.642136
39	1	0	-2.341503	0.064529	-0.560586

40	1	0	-6.713497	-3.200781	-1.522053
41	1	0	-8.328442	-1.519506	-1.226506
42	1	0	-9.116620	0.297109	0.145659
43	1	0	-8.864167	2.547363	1.084598
44	1	0	-4.724320	2.546673	-0.136492
45	1	0	-0.427613	-3.043506	0.215036
46	1	0	2.347835	0.220296	-0.426954
47	1	0	5.142873	-2.252230	1.693742
48	1	0	3.260937	-3.692333	2.478110
49	1	0	0.948709	-3.185319	1.813873
50	1	0	10.419997	0.823140	0.151986
51	1	0	7.624865	3.399325	-1.875697
52	1	0	5.735271	1.927444	-1.111328
53	1	0	-7.123914	5.624468	1.968177
54	1	0	-8.381568	4.849581	0.964230
55	1	0	-7.934251	4.141817	2.546610

Visualization of calculated geometry of (**5g*E***) :

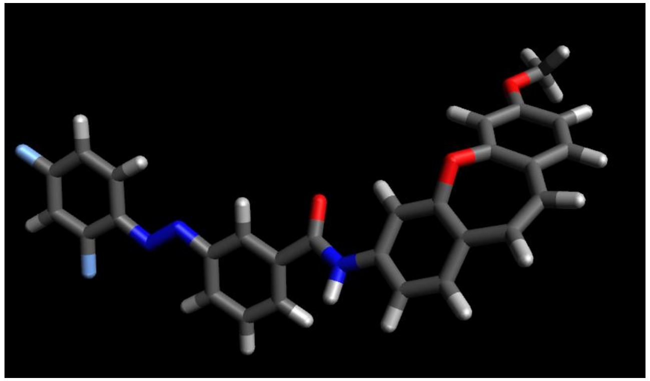

- The calculated coordinates of (**5g*Z***) (the part of calculated log file) -

Standard orientation:

Center Number	Atomic Number	Atomic Type	Coordinates (Angstroms) X	Y	Z
1	6	0	-0.387203	-0.474291	0.019188
2	6	0	-0.293986	-1.738959	0.623821
3	6	0	-1.399890	-2.574907	0.662832
4	6	0	-2.642641	-2.198733	0.118626
5	6	0	-2.700312	-0.932465	-0.493403
6	6	0	-1.607211	-0.072940	-0.542714

7	6	0	-3.783141	-3.106381	0.213730
8	6	0	-5.093681	-2.779019	0.207329
9	6	0	-5.678342	-1.445244	0.097177
10	6	0	-5.022481	-0.354586	-0.513226
11	8	0	-3.825320	-0.522851	-1.200945
12	6	0	-6.957841	-1.194260	0.618624
13	6	0	-7.559440	0.062228	0.571033
14	6	0	-6.870895	1.125955	-0.025976
15	6	0	-5.595863	0.905783	-0.570363
16	7	0	0.776721	0.317295	0.007381
17	6	0	0.961473	1.590559	-0.480451
18	8	0	0.088999	2.231346	-1.062188
19	6	0	2.341287	2.156012	-0.275498
20	6	0	3.239283	1.649620	0.669963
21	6	0	4.510586	2.212274	0.802139
22	6	0	4.881211	3.322452	0.037838
23	6	0	3.987634	3.820098	-0.911379
24	6	0	2.726013	3.246644	-1.067136
25	7	0	5.384967	1.752464	1.847146
26	7	0	5.787282	0.572904	1.890836
27	8	0	-7.348087	2.395555	-0.141712
28	6	0	-8.643428	2.678968	0.379129
29	6	0	5.565585	-0.356000	0.794154
30	6	0	4.583802	-1.344110	0.837267
31	6	0	4.493056	-2.351399	-0.112674
32	6	0	5.446225	-2.367261	-1.124968
33	6	0	6.450305	-1.409344	-1.210547
34	6	0	6.509600	-0.412200	-0.238589
35	9	0	3.630962	-1.283005	1.805872
36	9	0	5.383346	-3.340624	-2.052835
37	1	0	0.647175	-2.061974	1.063549
38	1	0	-1.310918	-3.550208	1.134529
39	1	0	-1.701844	0.881857	-1.037741
40	1	0	-3.528206	-4.149977	0.389561
41	1	0	-5.808796	-3.581997	0.378276
42	1	0	-7.497256	-2.015554	1.084422

43	1	0	-8.548981	0.196397	0.991696
44	1	0	-5.070843	1.717703	-1.061938
45	1	0	1.589113	-0.142174	0.394993
46	1	0	2.959258	0.860349	1.357086
47	1	0	5.858980	3.773715	0.179425
48	1	0	4.275470	4.668193	-1.525815
49	1	0	2.019561	3.638779	-1.790682
50	1	0	-8.817695	3.738538	0.184624
51	1	0	-9.415619	2.083796	-0.123626
52	1	0	-8.688670	2.495366	1.459791
53	1	0	3.710594	-3.100637	-0.062215
54	1	0	7.174580	-1.453716	-2.016896
55	1	0	7.299579	0.332205	-0.270703

Visualization of calculated geometry of (**5g*Z***) :

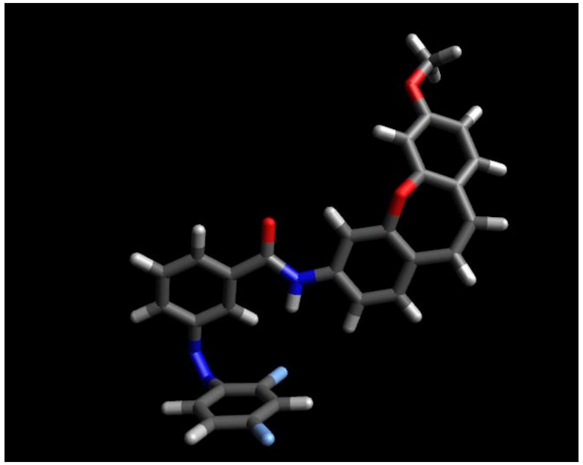

- The calculated coordinates of (**5h*E***) (the part of calculated log file) -

Standard orientation:

Center Number	Atomic Number	Atomic Type	Coordinates (Angstroms) X	Y	Z
1	6	0	-1.995284	-2.076149	-0.211396
2	6	0	-2.660410	-3.313355	-0.221754
3	6	0	-4.011557	-3.376555	-0.529523
4	6	0	-4.765726	-2.227557	-0.833183
5	6	0	-4.067785	-1.005302	-0.834854
6	6	0	-2.715169	-0.913301	-0.523969
7	6	0	-6.193215	-2.346951	-1.118210
8	6	0	-7.143121	-1.399554	-0.959275
9	6	0	-6.966691	-0.033938	-0.472601
10	6	0	-5.749127	0.674708	-0.563869
11	8	0	-4.669609	0.167760	-1.278910
12	6	0	-8.040793	0.641747	0.128673

13	6	0	-7.929241	1.934075	0.639492
14	6	0	-6.700057	2.599351	0.548638
15	6	0	-5.609720	1.958084	-0.060106
16	7	0	-0.624738	-2.077928	0.113636
17	6	0	0.252573	-1.018733	0.160922
18	8	0	-0.052261	0.133498	-0.140848
19	6	0	1.648321	-1.363631	0.601288
20	6	0	2.682176	-0.527916	0.163284
21	6	0	4.005822	-0.775316	0.536060
22	6	0	4.306286	-1.862160	1.377685
23	6	0	3.278808	-2.678991	1.832663
24	6	0	1.954588	-2.436700	1.449552
25	7	0	4.961428	0.136293	0.011414
26	7	0	6.150684	-0.125817	0.349287
27	6	0	7.147958	0.739159	-0.128837
28	6	0	8.470543	0.391965	0.223595
29	6	0	9.579626	1.132160	-0.155952
30	6	0	9.383705	2.281286	-0.922265
31	6	0	8.098382	2.676323	-1.298088
32	6	0	7.008143	1.913410	-0.904738
33	9	0	8.659838	-0.713246	0.961435
34	9	0	5.790263	2.326677	-1.280621
35	8	0	-6.461306	3.860812	1.001411
36	6	0	-7.532678	4.570150	1.616590
37	1	0	-2.115071	-4.225467	0.009898
38	1	0	-4.509797	-4.342791	-0.531362
39	1	0	-2.224156	0.047667	-0.556116
40	1	0	-6.522728	-3.341506	-1.413826
41	1	0	-8.175888	-1.694448	-1.137877
42	1	0	-8.997467	0.130080	0.203157
43	1	0	-8.793112	2.404951	1.093560
44	1	0	-4.664101	2.479823	-0.161175
45	1	0	-0.226809	-2.997013	0.258738
46	1	0	2.459951	0.320445	-0.474704
47	1	0	5.337401	-2.038940	1.662779
48	1	0	3.500019	-3.509308	2.497387
49	1	0	1.168754	-3.073085	1.848541
50	1	0	10.567652	0.804726	0.149045
51	1	0	10.238719	2.875589	-1.229662
52	1	0	7.925181	3.567880	-1.891777
53	1	0	-7.122867	5.541187	1.899158
54	1	0	-8.368186	4.715507	0.920605
55	1	0	-7.892790	4.051584	2.513776

Visualization of calculated geometry of (**5h*E***) :

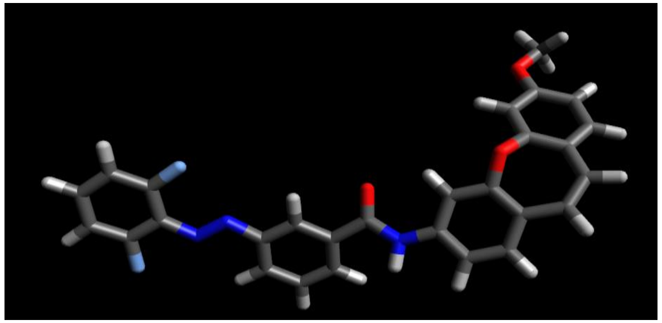

- The calculated coordinates of (**5h*Z***) (the part of calculated log file) -

Standard orientation:

Center Number	Atomic Number	Atomic Type	Coordinates (Angstroms) X	Y	Z
1	6	0	0.579362	-0.629970	-1.112025
2	6	0	0.755061	-1.793219	-1.878277
3	6	0	1.917674	-2.540140	-1.752903
4	6	0	2.953401	-2.174199	-0.872845
5	6	0	2.739618	-1.014280	-0.103950
6	6	0	1.588707	-0.242050	-0.218243
7	6	0	4.168404	-2.981633	-0.802249
8	6	0	5.401158	-2.577192	-0.425408
9	6	0	5.808546	-1.242308	0.004232
10	6	0	4.921771	-0.300494	0.569681
11	8	0	3.619010	-0.648521	0.909768
12	6	0	7.144402	-0.835104	-0.141721
13	6	0	7.590444	0.435112	0.220091
14	6	0	6.677640	1.350700	0.759193
15	6	0	5.337562	0.970359	0.933160
16	7	0	-0.639145	0.064352	-1.249236
17	6	0	-1.011818	1.262026	-0.683243
18	8	0	-0.262372	1.963785	-0.008600
19	6	0	-2.435010	1.675070	-0.940632
20	6	0	-3.475850	0.751020	-1.084287
21	6	0	-4.785803	1.207677	-1.276040
22	6	0	-5.054528	2.576433	-1.379619
23	6	0	-4.020278	3.493153	-1.194777
24	6	0	-2.719619	3.047641	-0.965894
25	7	0	-5.862545	0.297769	-1.531358
26	7	0	-6.127987	-0.658671	-0.775645
27	6	0	-5.485862	-0.816177	0.511060
28	6	0	-4.677276	-1.923322	0.770422
29	6	0	-4.201489	-2.224203	2.037375

30	6	0	-4.577441	-1.401599	3.102601
31	6	0	-5.397811	-0.292436	2.891145
32	6	0	-5.839222	-0.019096	1.603086
33	9	0	-4.299576	-2.673758	-0.289171
34	9	0	-6.634951	1.045537	1.378847
35	8	0	6.985297	2.615764	1.156939
36	6	0	8.334371	3.054451	1.025646
37	1	0	-0.022112	-2.113591	-2.568397
38	1	0	2.038301	-3.436725	-2.355695
39	1	0	1.473557	0.637991	0.396668
40	1	0	4.068041	-3.999165	-1.175783
41	1	0	6.212095	-3.297482	-0.519959
42	1	0	7.857674	-1.540556	-0.561289
43	1	0	8.633985	0.692887	0.083927
44	1	0	4.633106	1.663465	1.380219
45	1	0	-1.282943	-0.334507	-1.921191
46	1	0	-3.289566	-0.317125	-1.048710
47	1	0	-6.068841	2.908058	-1.581864
48	1	0	-4.229286	4.557858	-1.240561
49	1	0	-1.906411	3.751060	-0.822339
50	1	0	-3.550791	-3.081544	2.174028
51	1	0	-4.222459	-1.623720	4.103870
52	1	0	-5.703640	0.357500	3.704399
53	1	0	8.350879	4.080123	1.397571
54	1	0	9.015353	2.437801	1.625362
55	1	0	8.659374	3.041963	-0.022021

Visualization of calculated geometry of (**5h*Z***) :

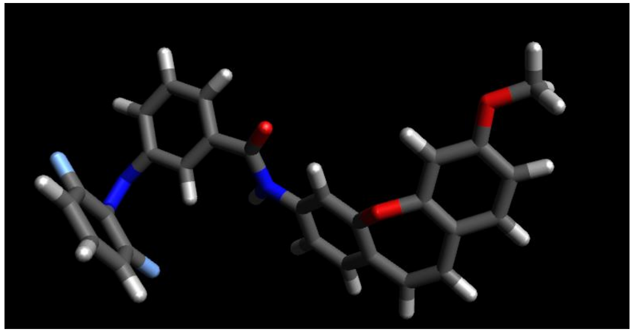

- The calculated coordinates of (**5h*E-syn***) (the part of calculated log file) -

Standard orientation:

Center Number	Atomic Number	Atomic Type	Coordinates (Angstroms) X	Y	Z
1	6	0	3.839931	0.051783	-0.840245

2	6	0	3.901615	0.518215	-2.160312
3	6	0	3.220316	1.675564	-2.520531
4	6	0	2.437270	2.398172	-1.601662
5	6	0	2.434283	1.933010	-0.272492
6	6	0	3.125541	0.791062	0.111543
7	6	0	1.651317	3.547215	-2.045681
8	6	0	0.498749	3.997122	-1.501213
9	6	0	-0.202157	3.462642	-0.336383
10	6	0	0.446951	2.769212	0.706895
11	8	0	1.835424	2.673191	0.741215
12	6	0	-1.592681	3.617398	-0.213501
13	6	0	-2.315932	3.096794	0.858073
14	6	0	-1.637677	2.404005	1.870292
15	6	0	-0.245650	2.247846	1.786970
16	7	0	4.542490	-1.129500	-0.495035
17	6	0	4.251857	-2.109784	0.426041
18	8	0	5.132649	-2.882655	0.801709
19	6	0	2.841702	-2.259147	0.920989
20	6	0	1.725781	-2.080535	0.095954
21	6	0	0.435023	-2.276864	0.599199
22	6	0	0.251954	-2.671405	1.935821
23	6	0	1.363053	-2.878582	2.746114
24	6	0	2.652737	-2.684990	2.242373
25	7	0	-0.611262	-2.051498	-0.332957
26	7	0	-1.761533	-2.004838	0.185465
27	6	0	-2.825863	-1.811607	-0.716324
28	6	0	-4.024704	-1.309777	-0.172976
29	6	0	-5.172405	-1.102261	-0.921884
30	6	0	-5.151659	-1.430486	-2.278187
31	6	0	-3.998086	-1.952177	-2.865809
32	6	0	-2.860128	-2.130704	-2.090779
33	9	0	-4.042521	-0.993293	1.135517
34	9	0	-1.778384	-2.664464	-2.677501
35	8	0	-2.222883	1.867107	2.974308
36	6	0	-3.645899	1.866631	3.051320
37	1	0	4.470512	-0.036587	-2.901234

38	1	0	3.265144	2.019144	-3.550984
39	1	0	3.111494	0.500819	1.155123
40	1	0	1.989461	4.015112	-2.968463
41	1	0	-0.018769	4.800540	-2.023284
42	1	0	-2.125862	4.155396	-0.993752
43	1	0	-3.389495	3.239283	0.896625
44	1	0	0.287715	1.728714	2.575658
45	1	0	5.478561	-1.229622	-0.883276
46	1	0	1.828339	-1.784576	-0.942082
47	1	0	-0.755788	-2.815270	2.309744
48	1	0	1.229998	-3.197780	3.776138
49	1	0	3.522550	-2.861984	2.867009
50	1	0	-6.056263	-0.696487	-0.441428
51	1	0	-6.041533	-1.281224	-2.881914
52	1	0	-3.965222	-2.227545	-3.914839
53	1	0	-3.890603	1.372614	3.993245
54	1	0	-4.049961	2.886492	3.063597
55	1	0	-4.082968	1.302373	2.220165

Visualization of calculated geometry of (**5h*E*-*syn***):

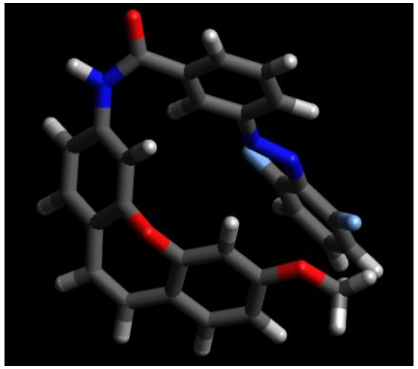

1.2 Molecular Orbitals-

1) Molecular orbitals for the **4a*E* model system,** HOMO on the left, LUMO on the right.

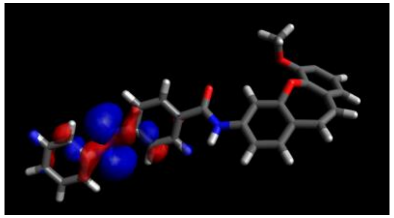

HOMO (-6.430 eV)

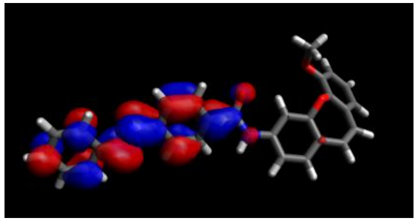

LUMO (-2.616 eV)

2) Molecular orbitals for the **4a*Z* model system,** HOMO on the left, LUMO on the right.

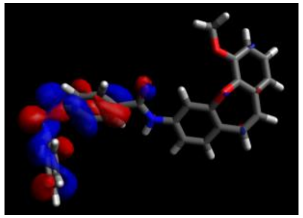

HOMO (-6.100 eV)

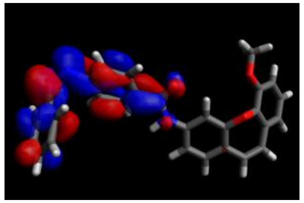

LUMO (-2.399 eV)

3) Molecular orbitals for the **4b*E* model system,** HOMO on the left, LUMO on the right.

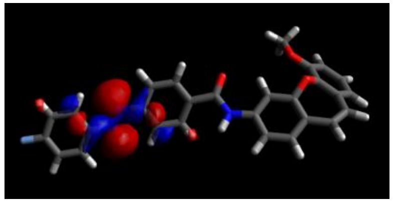

HOMO (-6.478 eV)

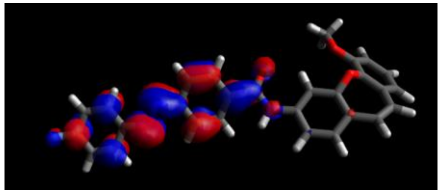

LUMO (-2.627 eV)

4) Molecular orbitals for the **4b*Z* model system,** HOMO on the left, LUMO on the right.

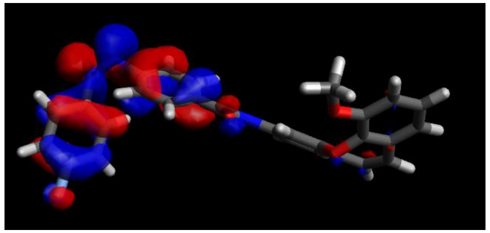

HOMO (-6.107 eV)

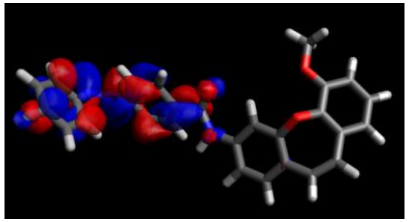

LUMO (-2.415 eV)

5) Molecular orbitals for the **4c*E* model system,** HOMO on the left, LUMO on the right.

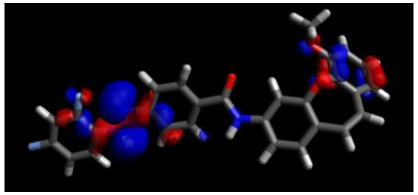

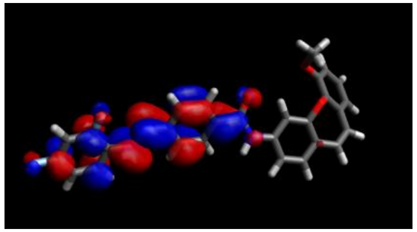

HOMO (-6. 410 eV)

LUMO (-2.641 eV)

6) Molecular orbitals for the **4c*Z* model system,** HOMO on the left, LUMO on the right.

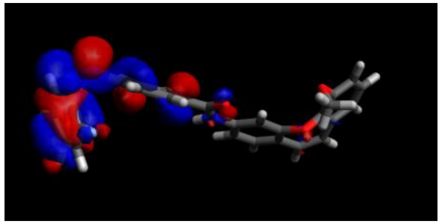

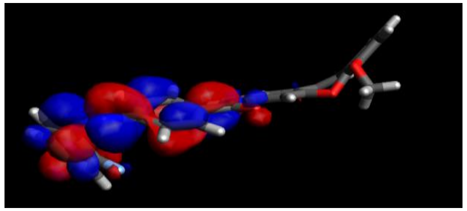

HOMO (-6. 228 eV)

LUMO (-2.517 eV)

7) Molecular orbitals for the **4d*E* model system,** HOMO on the left, LUMO on the right.

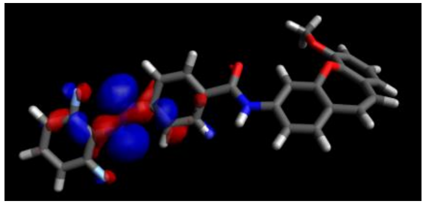

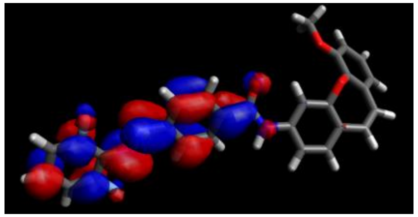

HOMO (-6.413 eV)

LUMO (-2.712 eV)

8) Molecular orbitals for the **4d*Z* model system,** HOMO on the left, LUMO on the right.

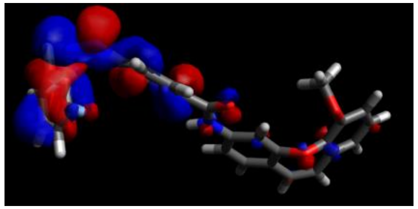

HOMO (-6.334 eV)

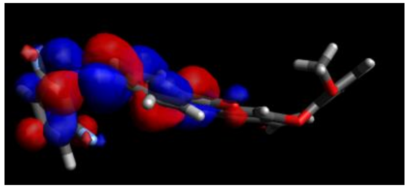

LUMO (-2.587 eV)

9) Molecular orbitals for the **4e*E* model system,** HOMO on the left, LUMO on the right.

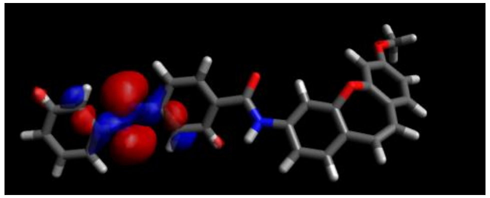

HOMO (-6.434 eV)

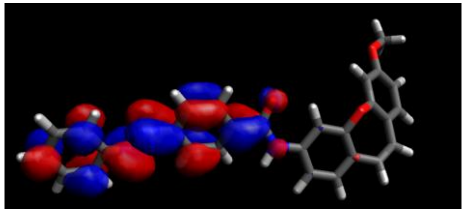

LUMO (-2.618 eV)

10) Molecular orbitals for the **4e*Z* model system,** HOMO on the left, LUMO on the right.

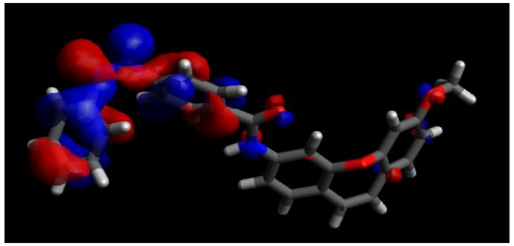

HOMO (-6.081 eV)

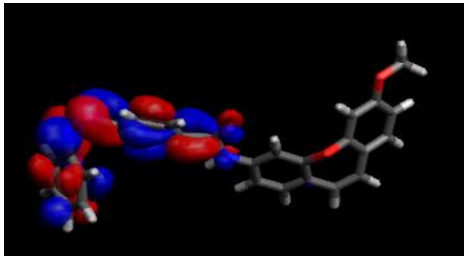

LUMO(-2.415 eV)

11) Molecular orbitals for the **4f*E* model system,** HOMO on the left, LUMO on the right.

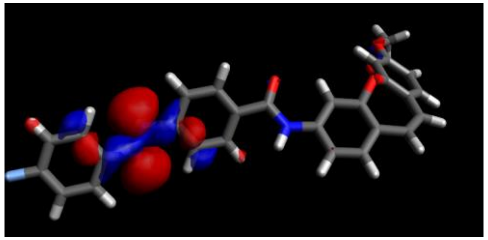

HOMO (-6.480 eV)

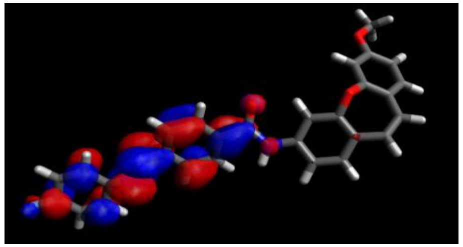

LUMO (-2.629 eV)

12) Molecular orbitals for the **4f*Z* model system,** HOMO on the left, LUMO on the right.

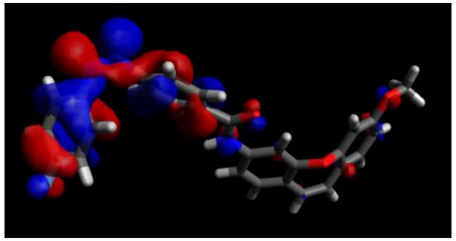

HOMO (-6.096 eV)

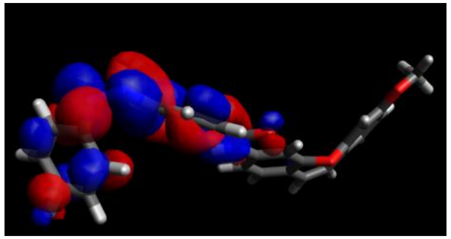

LUMO (-2.433 eV)

13) Molecular orbitals for the **4g*E* model system,** HOMO on the left, LUMO on the right.

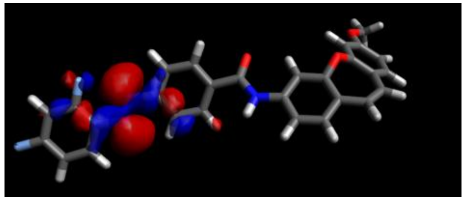

HOMO (-6.409 eV)

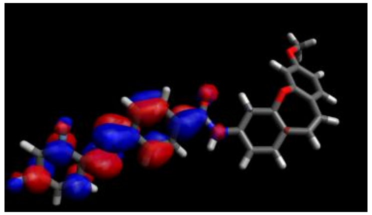

LUMO (-2.639 eV)

14) Molecular orbitals for the **4g*Z* model system,** HOMO on the left, LUMO on the right.

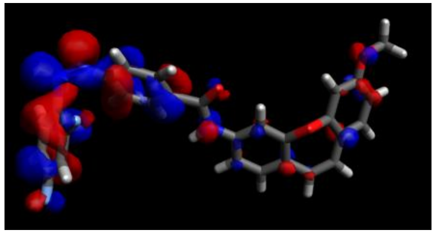

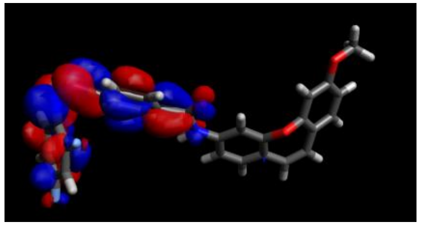

HOMO (-6.220 eV) LUMO (-2.525 eV)

15) Molecular orbitals for the **4h*E* model system,** HOMO on the left, LUMO on the right.

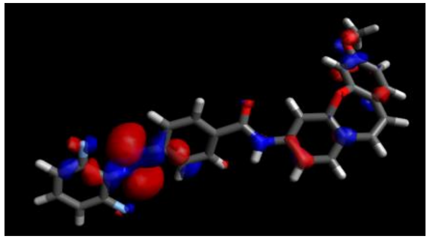

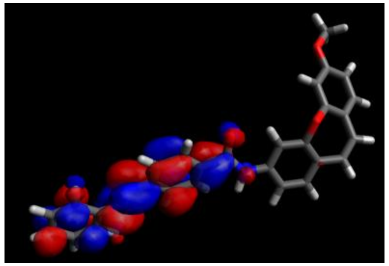

HOMO (-6.422 eV) LUMO (-2.717 eV)

16) Molecular orbitals for the **4h*Z* model system,** HOMO on the left, LUMO on the right.

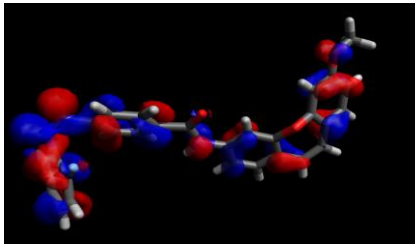

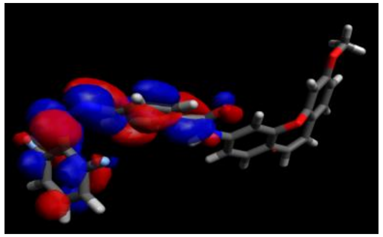

HOMO (-6.311 eV) LUMO (-2.577 eV)

17) Molecular orbitals for the **5a*E* model system,** HOMO on the left, LUMO on the right.

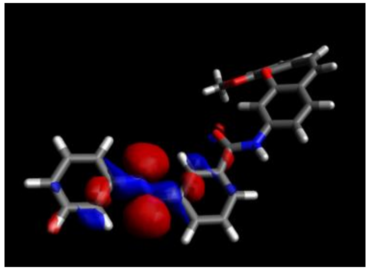

HOMO (-6.403 eV)

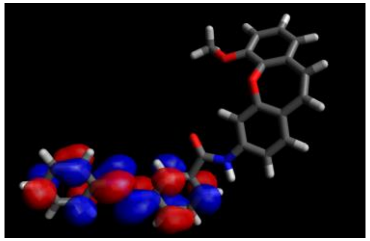

LUMO (-2.465 eV)

18) Molecular orbitals for the **5a*Z* model system,** HOMO on the left, LUMO on the right.

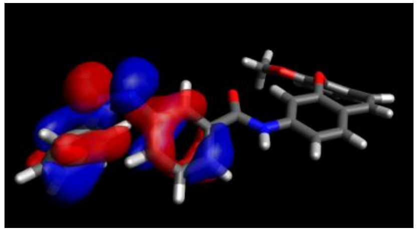

HOMO (-6.075 eV)

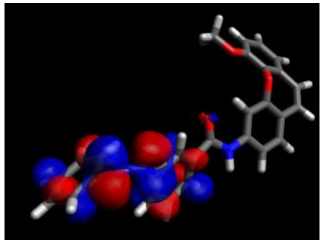

LUMO (-2.280 eV)

19) Molecular orbitals for the **5b*E* model system,** HOMO on the left, LUMO on the right.

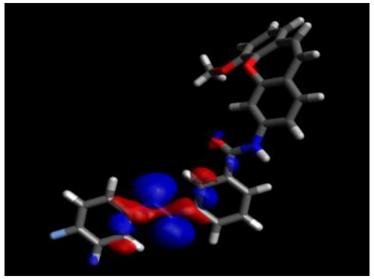

HOMO (-6.452 eV)

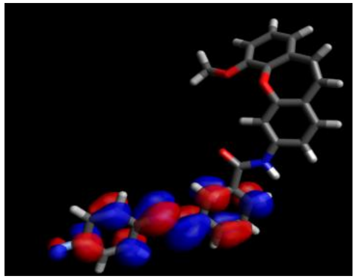

LUMO (-2.479 eV)

20) Molecular orbitals for the **5b*Z* model system,** HOMO on the left, LUMO on the right.

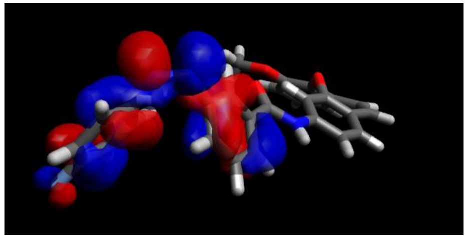

HOMO (-6.088 eV)

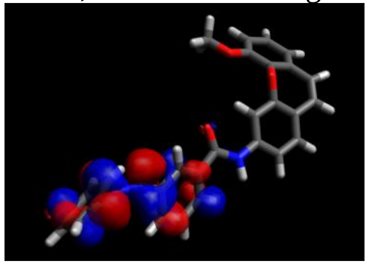

LUMO (-2.315 eV)

21) Molecular orbitals for the **5c*E* model system,** HOMO on the left, LUMO on the right.

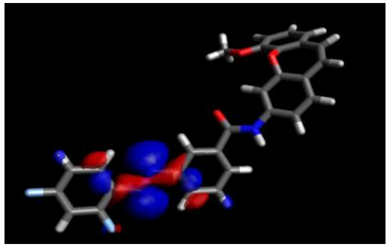

HOMO (-6.529 eV)

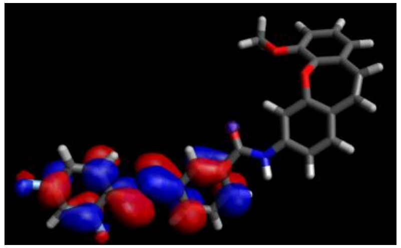

LUMO (-2.592 eV)

22) Molecular orbitals for the **5c*Z* model system,** HOMO on the left, LUMO on the right.

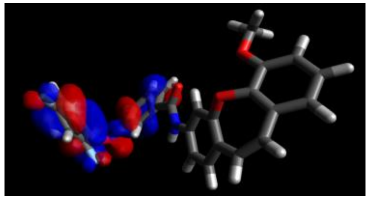

HOMO (-6.314 eV)

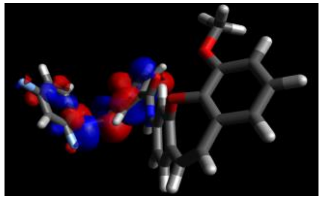

LUMO (-2.230 eV)

23) Molecular orbitals for the **5d*E* model system,** HOMO on the left, LUMO on the right.

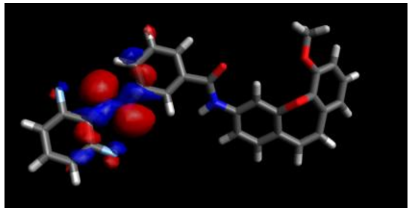

HOMO (-6.418 eV)

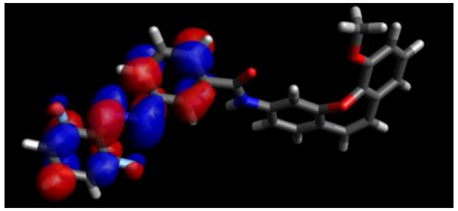

LUMO (-2.601 eV)

24) Molecular orbitals for the **5d*Z* model system,** HOMO on the left, LUMO on the right.

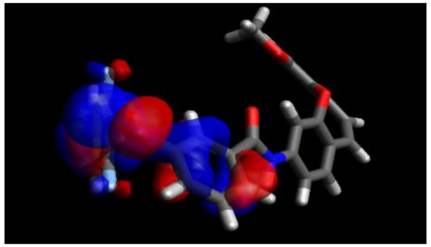

HOMO (-6.328 eV)

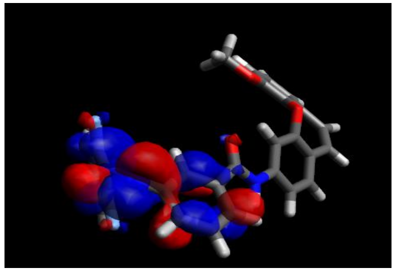

LUMO (-2.448 eV)

25) Molecular orbitals for the **5e*E* model system,** HOMO on the left, LUMO on the right.

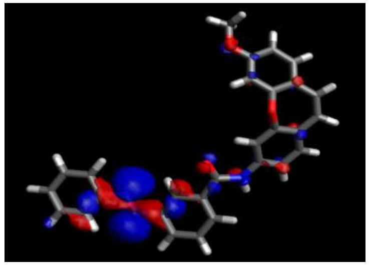

HOMO (-6.408 eV)

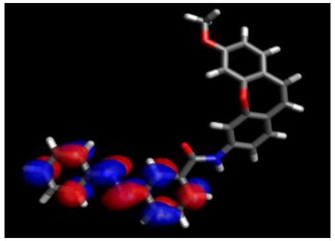

LUMO (-2.466 eV)

26) Molecular orbitals for the **5e*Z* model system,** HOMO on the left, LUMO on the right.

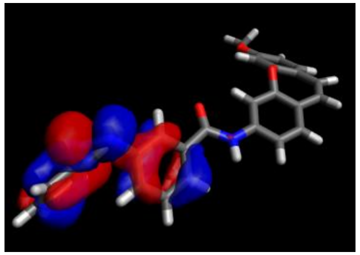

HOMO (-6. 067 eV)

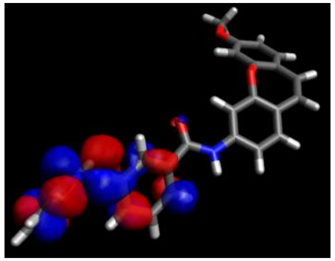

LUMO (-2.287 eV)

27) Molecular orbitals for the **5f*E* model system,** HOMO on the left, LUMO on the right.

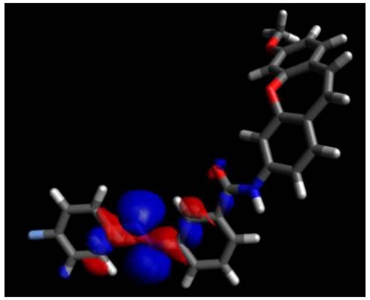

HOMO (-6.453 eV)

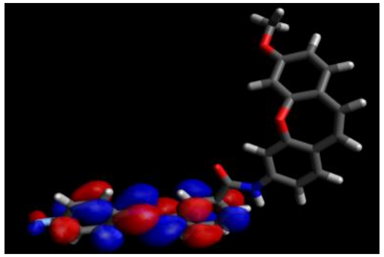

LUMO (-2.48 eV)

28) Molecular orbitals for the **5f*Z* model system,** HOMO on the left, LUMO on the right.

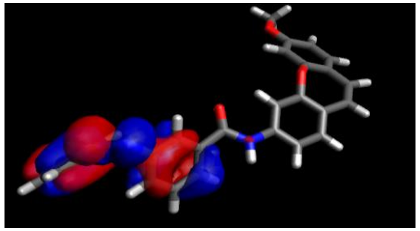

HOMO (-6.091 eV)

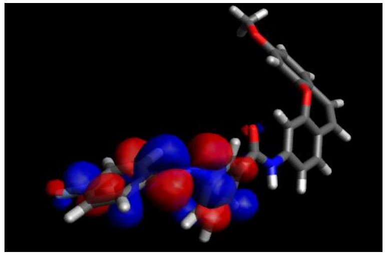

LUMO (-2. 309 eV)

29) Molecular orbitals for the **5g*E* model system,** HOMO on the left, LUMO on the right.

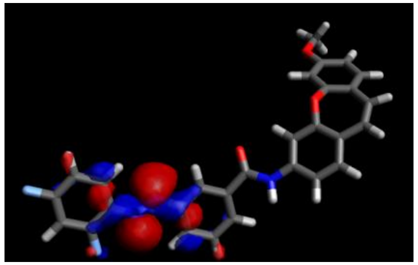

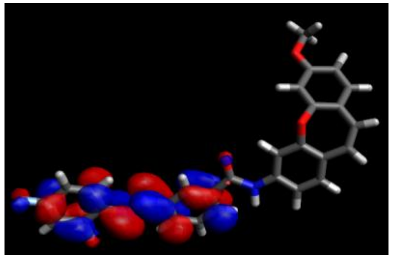

HOMO (-6.532 eV)

LUMO (-2.593 eV)

30) Molecular orbitals for the **5g*Z* model system,** HOMO on the left, LUMO on the right.

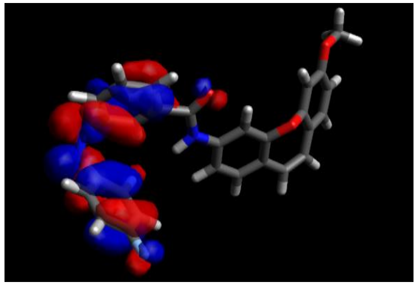

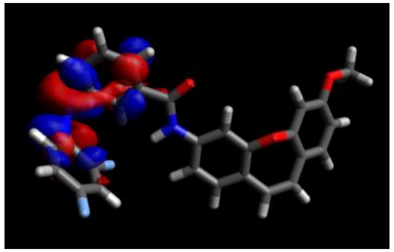

HOMO (-6.434 eV)

LUMO (-2.347 eV)

31) Molecular orbitals for the **5h*E* model system,** HOMO on the left, LUMO on the right.

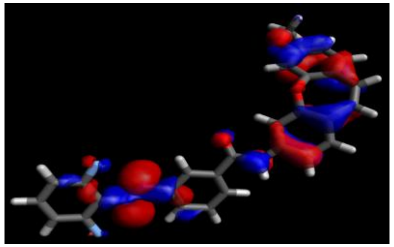

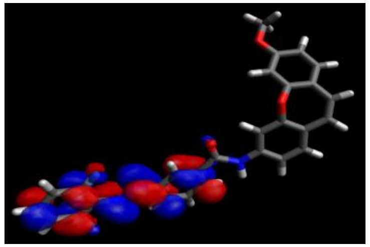

HOMO (-6.401 eV)

LUMO (-2. 595 eV)

32) Molecular orbitals for the **5h*Z* model system,** HOMO on the left, LUMO on the right.

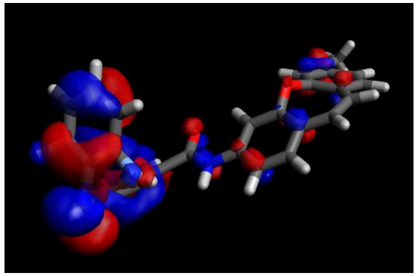

HOMO (-6.442 eV)

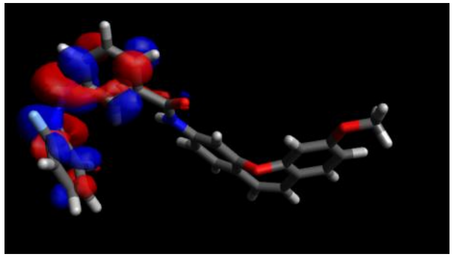

LUMO (-2.416 eV)

Table 1S. Dihedral angels for the C-N=N-C bond [°] and distance[Å] within investigated model systems obtained from optimized geometries.

**Compound**	**Dihedral angle [°]**	**Distance [Å]**
	C-N=N-C	CO----H in dibenzo[*b*,*f*]oxepine
**4a *E***	180	2.223
**4a *Z***	10.4	2.227
**4b *E***	179.9	2.226
**4b *Z***	10.2	2.229
**4c *E***	180	2.217
**4c *Z***	9.8	2.218
**4d *E***	179.7	2.227
**4d *Z***	10.6	2.211
**4e *E***	179.9	2.211
**4e *Z***	11.2	2.211
**4f *E***	179.9	2.211

**4f *Z***	10.7	2.210
**4g *E***	180	2.212
**4g *Z***	9.8	2.209
**4h *E***	179.7	2.209
**4h *Z***	10.6	2.209
**5a *E***	179.8	2.224
**5a *Z***	9.9	2.226
**5b *E***	179.8	2.224
**5b *Z***	9.9	2.231
**5c *E***	180	2.225
**5c *Z***	9.6	2.229
**5d *E***	180	2.225
**5d *Z***	10.4	2.225
**5e *E***	179.8	2.215
**5e *Z***	10.6	2.217
**5f *E***	179.9	2.211
**5f *Z***	9.7	2.206
**5g *E***	179.8	2.214
**5g *Z***	9.3	2.243
**5h *E***	179.7	2.213
**5h *Z***	8.6	2.222

1.3. Molecular docking

Molecular docking of compounds of **4a-4h and 5a-5h** isomers ***E*** and ***Z*** into the 3D X-ray structure of tubulin (PDB code: 1SA0)[3] as carried out using the Auto-Dock Vina software (the Broyden-Fletcher-Goldfarb-Shanno (BFGS) method) [4]. Configurations of protein/dimethoxydibenzo[*b,f*]oxepine complex were created using UCSF Chimera software [5].The graphical user interface ADT was employed to set up the enzyme: all hydrogens were added. For macromolecules, generated pdbqt files were saved. The 3D structures of ligand molecules were built, optimized (B3LYP functional and 6-31* basis set level, for ***E, Z* isomer;** and saved in Mol2 format. The graphical user interface ADT was also employed to set up the ligand and the pdbqt file was saved. Auto-Dock Vina software was employed for all docking calculations. The AutoDockTools program was used to generate the docking input files. In docking a grid box size 21 x 21 x 21 points in x, y, and z directions were built, the maps were center located (39.82, 53.24, -8.21) in the catalytic site of the protein. A grid spacing of 0.375 A (approximately one-fourth of the length of a carbon-carbon covalent bond) was used for the calculation of the energetic map. All computations were performed on an Intel®CoreTM i7-4702MQ 3.2 GHz processor running Ubuntu 18.04 Work-station Linux distribution. PyMOL software (www.pymol.org/) was used to analyze the docking results [6]. The Protein-Ligand Interaction Profiler (PLIP) was used in order to predict protein-docked ligand interactions [7]

Table 2S. The binding position, type of interaction and active residues for *E* and *Z* isomers 4a-4h and 5a-5h.

<table>
<tr><th>Compound</th><th>Binding pose and interactions</th><th>Type of interaction</th><th>Active residues</th></tr>
<tr><td rowspan="7">4aE</td><td rowspan="7">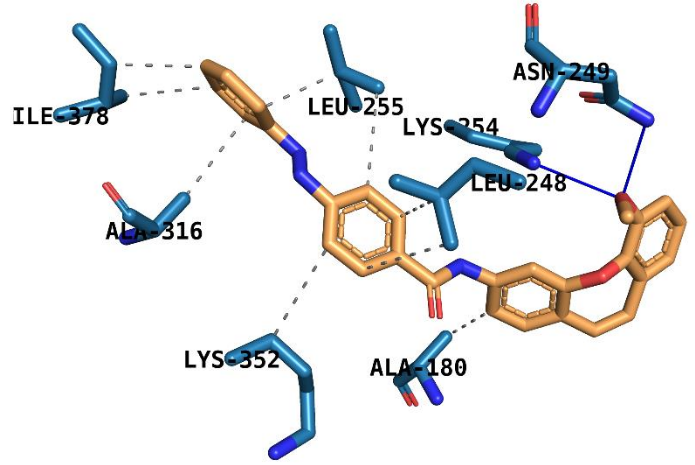
</td><td rowspan="5">Hydrophobic Interactions</td><td>Ala180</td></tr>
<tr><td>Leu248</td></tr>
<tr><td>Ala316</td></tr>
<tr><td>Lys352</td></tr>
<tr><td>Ile378</td></tr>
<tr><td rowspan="2">Hydrogen Bonds</td><td>Asn249</td></tr>
<tr><td>Lys254</td></tr>
<tr><td></td><td></td><td rowspan="2">Hydrophobic Interactions</td><td>Ala180</td></tr>
<tr><td></td><td></td><td>Val238</td></tr>
</table>

**4a*Z***	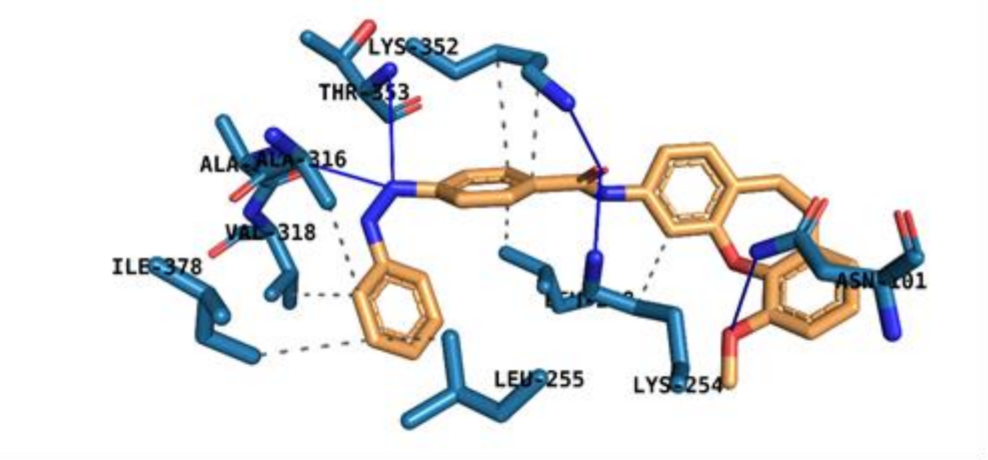		Leu248
			Lys254
			Leu255
			Ala316
			Val318
			Ala354
			Ile378
		Hydrogen Bonds	Asn101
			Lys254
			Ala317
			Lys352
			Thr353
**4b*E***	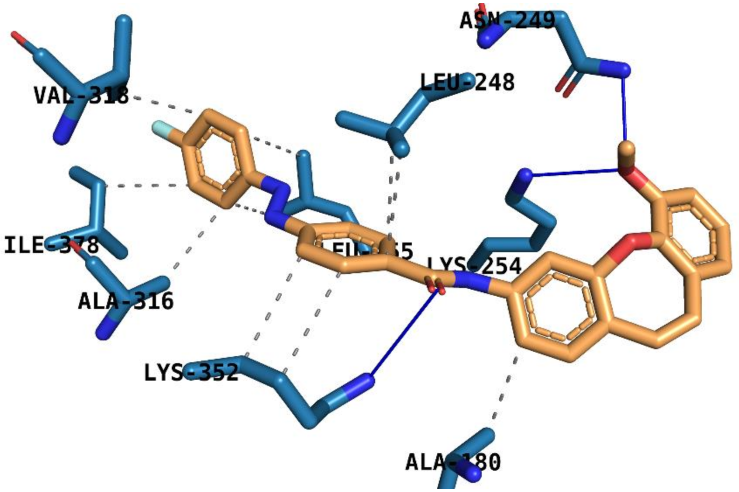	Hydrophobic Interactions	Ala180
			Leu248
			Leu255
			Ala316
			Val318
			Lys352
			Ile378
		Hydrogen Bonds	Asn249
			Lys254
			Lys352
**4b*Z***		Hydrophobic Interactions	Leu248

<table>
<tr><td rowspan="11"></td><td rowspan="11">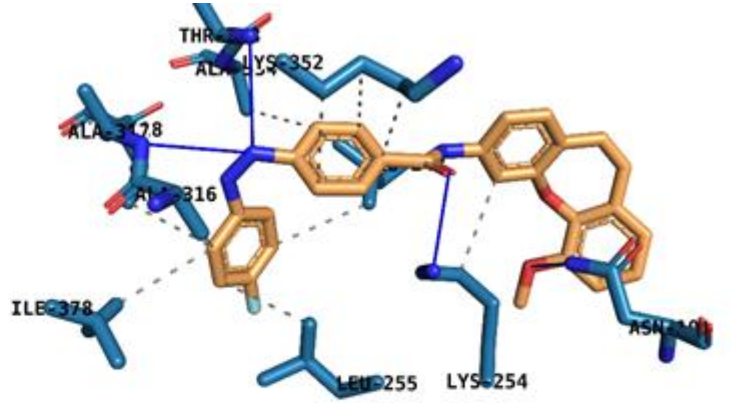
</td><td rowspan="7"></td><td>Lys254</td></tr>
<tr><td>Leu255</td></tr>
<tr><td>Ala316</td></tr>
<tr><td>Val318</td></tr>
<tr><td>Lys352</td></tr>
<tr><td>Ala354</td></tr>
<tr><td>Ile378</td></tr>
<tr><td rowspan="4">Hydrogen Bonds</td><td>Asn101</td></tr>
<tr><td>Lys254</td></tr>
<tr><td>Ala317</td></tr>
<tr><td>Thr353</td></tr>
<tr><td rowspan="7">4cE</td><td rowspan="7">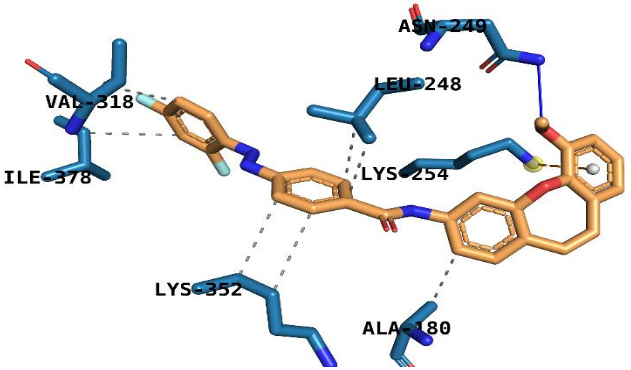
</td><td rowspan="5">Hydrophobic Interactions</td><td>Ala180</td></tr>
<tr><td>Leu248</td></tr>
<tr><td>Val318</td></tr>
<tr><td>Lys352</td></tr>
<tr><td>Ile378</td></tr>
<tr><td>Hydrogen Bonds</td><td>Asn249</td></tr>
<tr><td>π-Cation Interactions</td><td>Lys254</td></tr>
<tr><td rowspan="7">4cZ</td><td rowspan="7">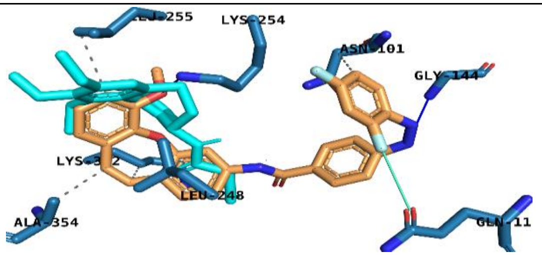
</td><td rowspan="5">Hydrophobic Interactions</td><td>Asn101</td></tr>
<tr><td>Leu248</td></tr>
<tr><td>Leu255</td></tr>
<tr><td>Lys352</td></tr>
<tr><td>Ala354</td></tr>
<tr><td rowspan="2">Hydrogen Bonds</td><td>Gly144</td></tr>
<tr><td>Lys253</td></tr>
</table>

		Halogen Bonds	Gln11
**4d*E***	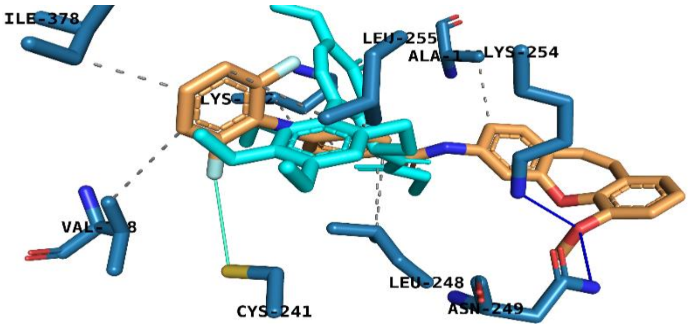	Hydrophobic Interactions	Ala180
			Leu248
			Leu255
			Val318
			Lys352
			Ile378
		Hydrogen Bonds	Asn249
			Lys254
		Halogen Bonds	Cys241
**4d*Z***	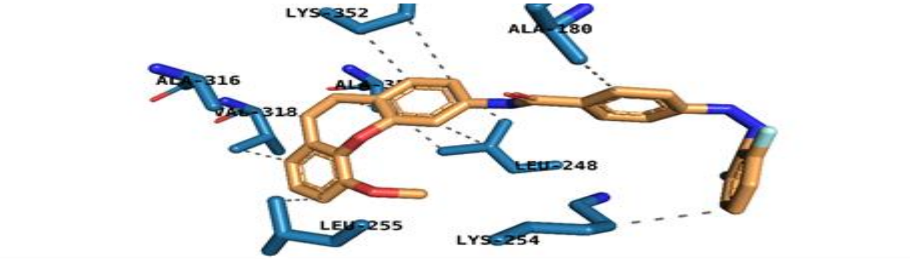	Hydrophobic Interactions	Ala180
			Leu248
			Lys254
			Leu255
			Ala180
			Val318
			Lys352
			Ala354
**4e*E***		Hydrophobic Interactions	Leu248
			Leu255
			Phe268
			Ala316
			Met325
			Val355

	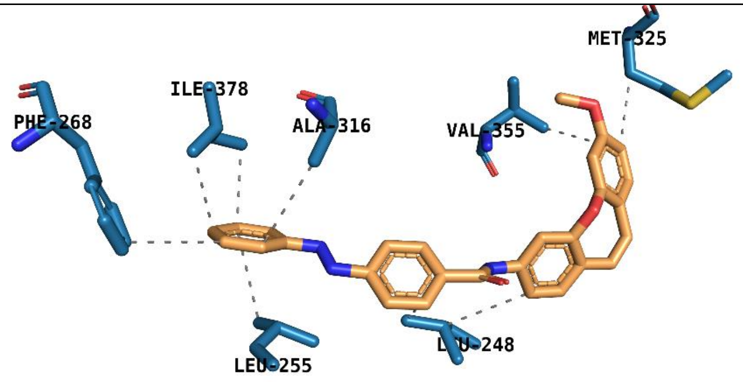		Ile378
**4e*Z***	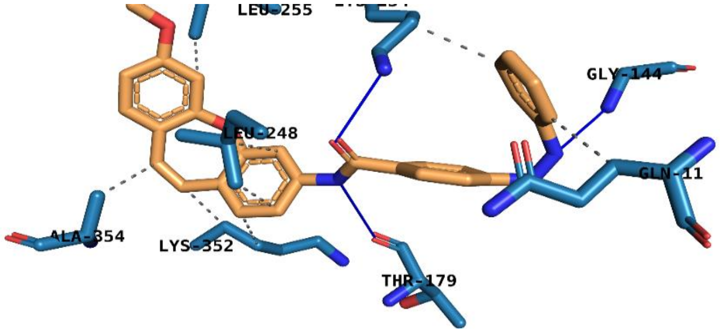	Hydrophobic Interactions	Gln11
			Leu248
			Lys254
			Leu255
			Lys352
			Ala354
		Hydrogen Bonds	Gly144
			Thr179
			Lys254
**4f*E***		Hydrophobic Interactions	Ala180
			Val238
			Leu248
			Ala250
			Ala316
			Val318

	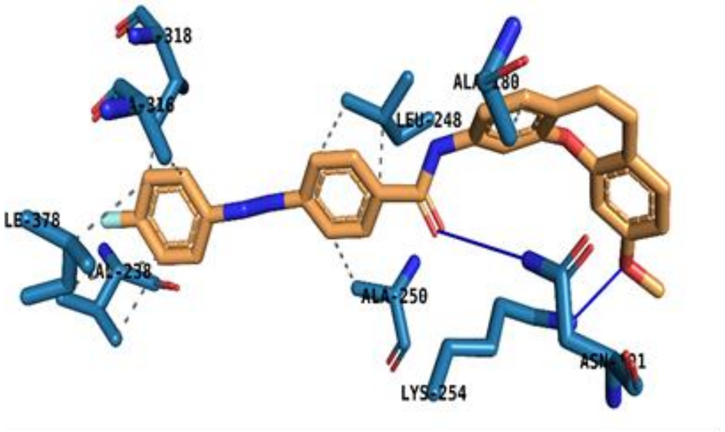		Ile378
		Hydrogen Bonds	Asn101
			Lys254
**4fZ**	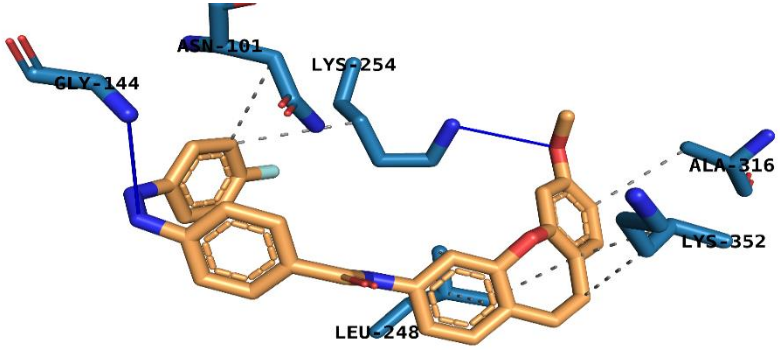	Hydrophobic Interactions	Asn101
			Leu248
			Lys254
			Ala316
			Lys352
		Hydrogen Bonds	Gly144
			Lys254

<table>
<tr><td rowspan="2">4gE</td><td rowspan="2">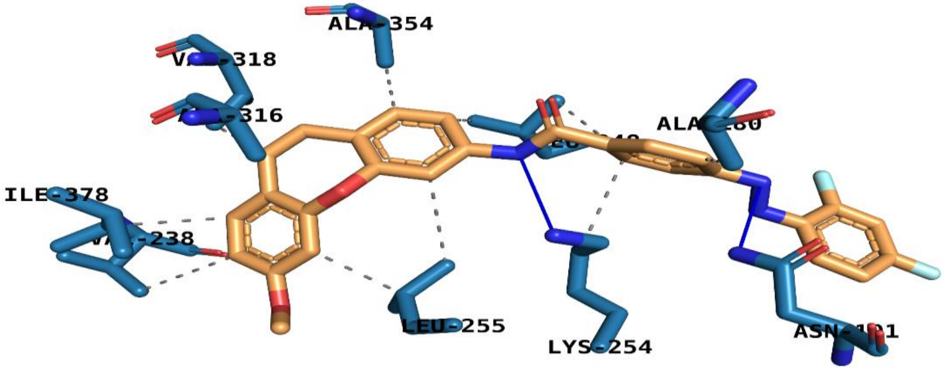
</td><td>Hydrophobic Interactions</td><td>Ala180
Val238
Leu248
Lys254
Leu255
Ala316
Val318
Ala354
Ile378</td></tr>
<tr><td>Hydrogen Bonds</td><td>Asn101
Lys254</td></tr>
<tr><td rowspan="2">4gZ</td><td rowspan="2">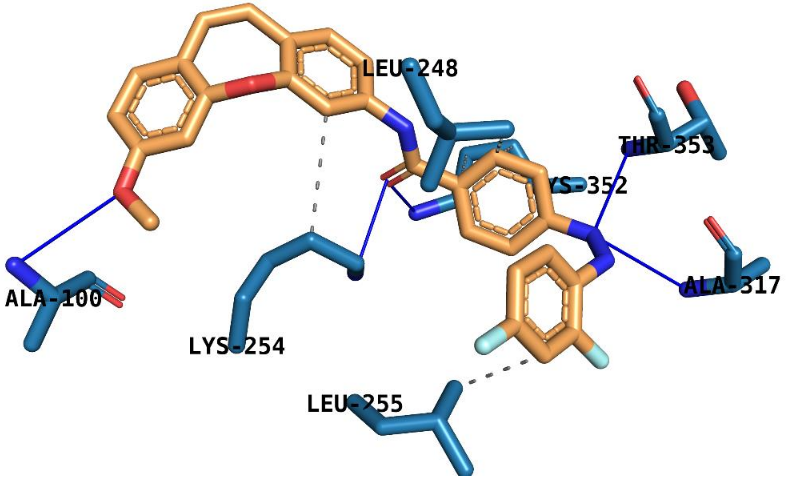
</td><td>Hydrophobic Interactions</td><td>Leu248
Lys254
Leu255
Lys352</td></tr>
<tr><td>Hydrogen Bonds</td><td>Ala100
Lys254
Ala317
Lys352
Thr353</td></tr>
</table>

<table>
<tr><td rowspan="4">4hE</td><td rowspan="4">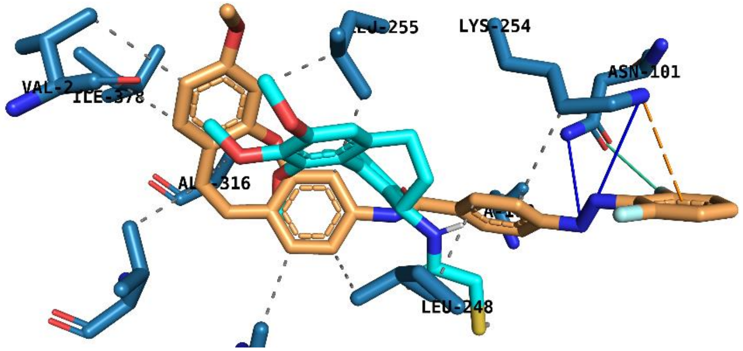
</td><td>Hydrophobic Interactions</td><td>Ala180
Val238
Leu248
Lys254
Leu255
Ala316
Val318
Ala354
Ile378
Asn101</td></tr>
<tr><td>Hydrogen Bonds</td><td>Asn101
Lys254</td></tr>
<tr><td>π-Cation Interactions</td><td>Lys254</td></tr>
<tr><td>Halogen Bonds</td><td>Asn101</td></tr>
<tr><td>4hZ</td><td>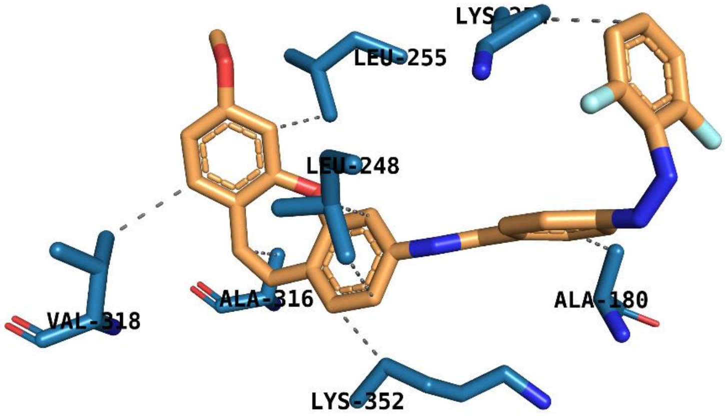
</td><td>Hydrophobic Interactions</td><td>Ala180
Leu248
Lys254
Leu255
Ala316
Val318
Lys352</td></tr>
</table>

Compound	Binding pose and interactions	Type of interaction	Active residues
**5a*E***	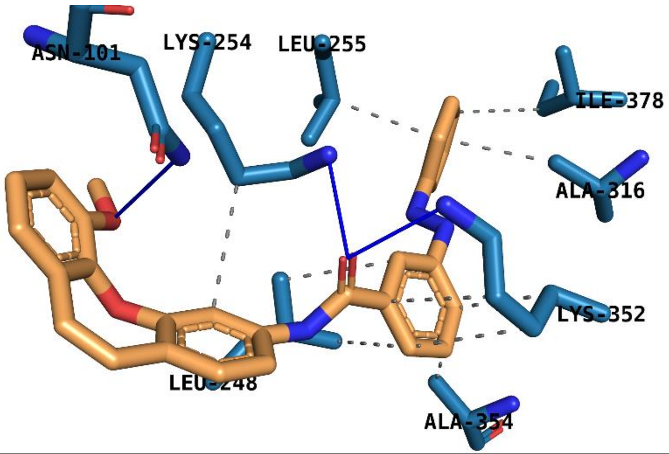	Hydrophobic Interactions	Leu248<br>Lys254<br>Leu255<br>Ala316<br>Lys352<br>Ala354<br>Ile378
		Hydrogen Bonds	Asn101<br>Lys254<br>Lys352
**5a*Z***	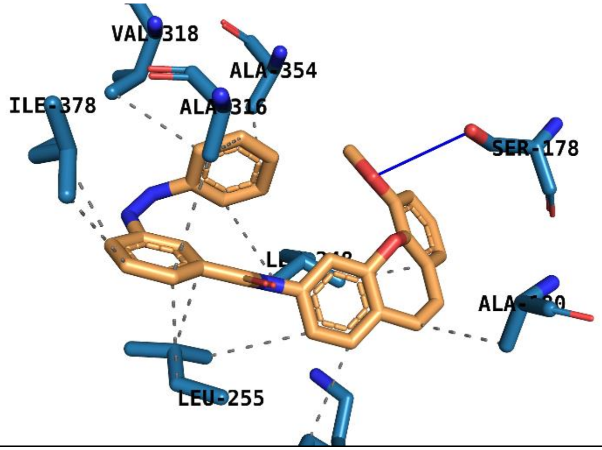	Hydrophobic Interactions	Ala180<br>Leu248<br>Lys254<br>Leu255<br>Ala316<br>Val318<br>Ala354<br>Ile378
		Hydrogen Bonds	Ser178

**5b*E***	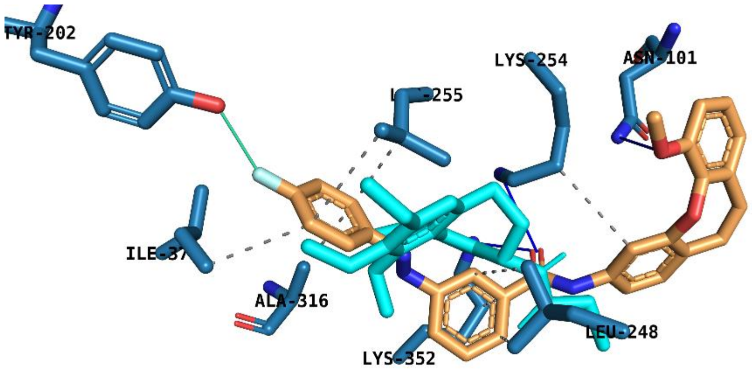	Hydrophobic Interactions	Leu248<br>Lys254<br>Leu255<br>Ala316<br>Lys352<br>Ile378
		Hydrogen Bonds	Asn101<br>Lys254<br>Lys352
		Halogen Bonds	Thr202
**5b*Z***	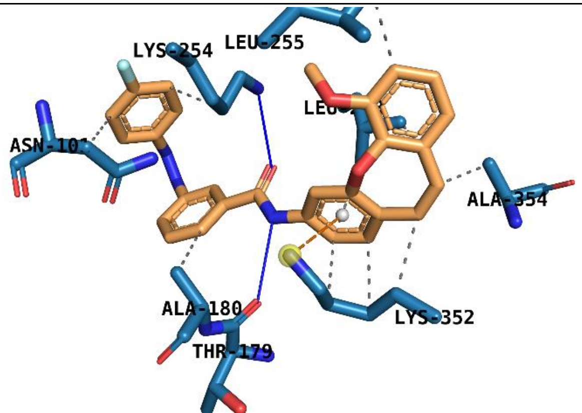	Hydrophobic Interactions	Asn101<br>Ala180<br>Leu248<br>Lys254<br>Leu255<br>Lys352<br>Ala354
		Hydrogen Bonds	Thr179<br>Lys254
		π-Cation Interactions	Lys352
**5c*E***	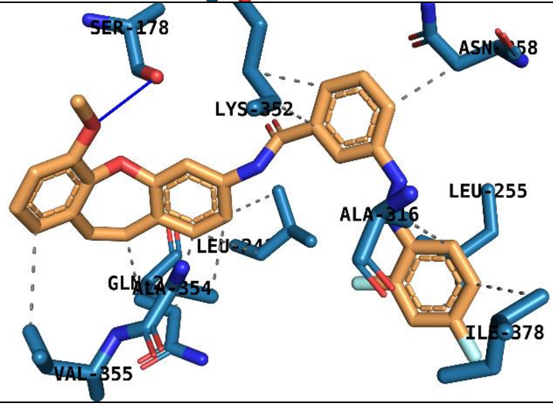	Hydrophobic Interactions	Gln247<br>Leu248<br>Leu255<br>Asn258<br>Ala316<br>Lys352<br>Ala354<br>Val355<br>Ile378
		Hydrogen Bonds	Ser178

**5c*Z***	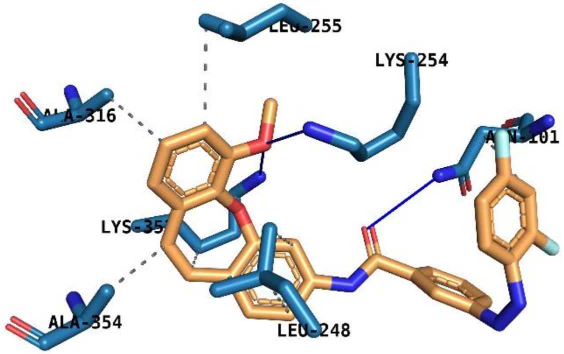	Hydrophobic Interactions	Asn101<br>Leu248<br>Leu255<br>Ala316<br>Lys352<br>Ala354
		Hydrogen Bonds	Asn101<br>Lys254<br>Lys352
**5d*E***	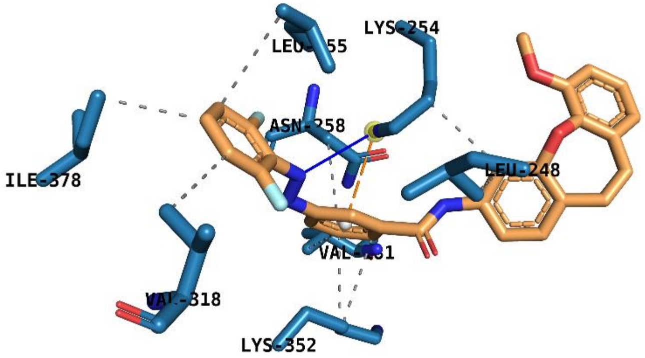	Hydrophobic Interactions	Val181<br>Leu248<br>Lys254<br>Leu255<br>Asn258<br>Val318<br>Lys352<br>Ile378
		Hydrogen Bonds	Lys254
		π-Cation Interactions	Lys254
**5dZ**	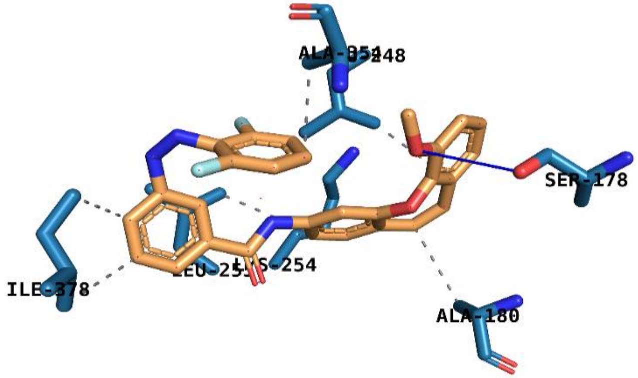	Hydrophobic Interactions	Ala180<br>Leu248<br>Lys254<br>Leu255<br>Ala354<br>Ile378
		Hydrogen Bonds	Ser178

5e*E*	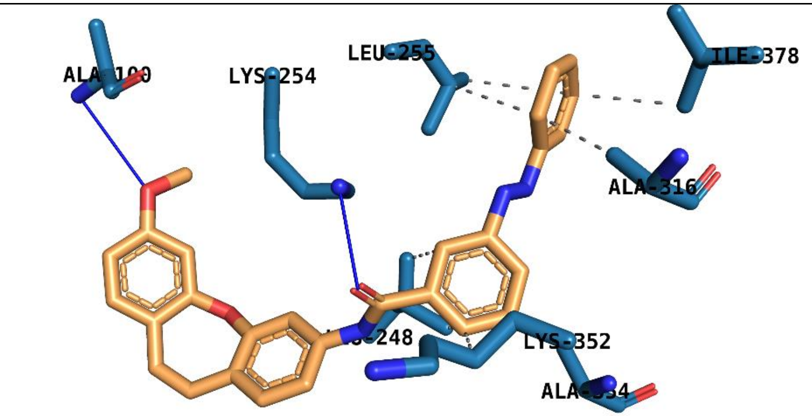	Hydrophobic Interactions	Leu248<br>Leu255<br>Ala316<br>Lys352<br>Ala354<br>Ile378
		Hydrogen Bonds	Ala100<br>Lys254
5e*Z*	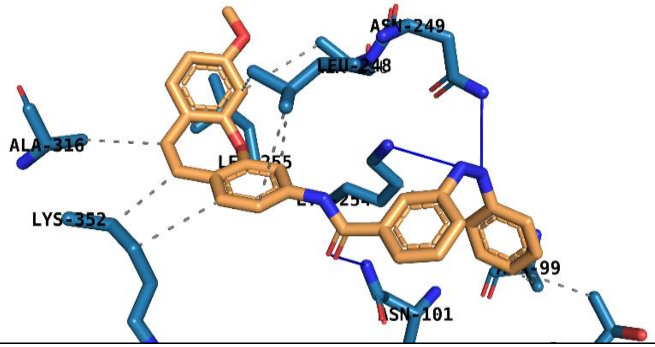	Hydrophobic Interactions	Ala99<br>Thr145<br>Leu248<br>Ala250<br>Lys254<br>Leu255<br>Ala316<br>Lys352
		Hydrogen Bonds	Asn101<br>Asn249<br>Lys254
5f*E*	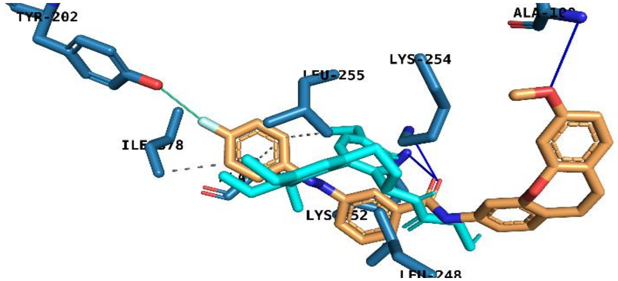	Hydrophobic Interactions	Leu248<br>Leu255<br>Ala316<br>Lys352<br>Ile378
		Hydrogen Bonds	Ala100<br>Lys254<br>Lys352

		Halogen Bonds	Tyr202
**5f*Z***	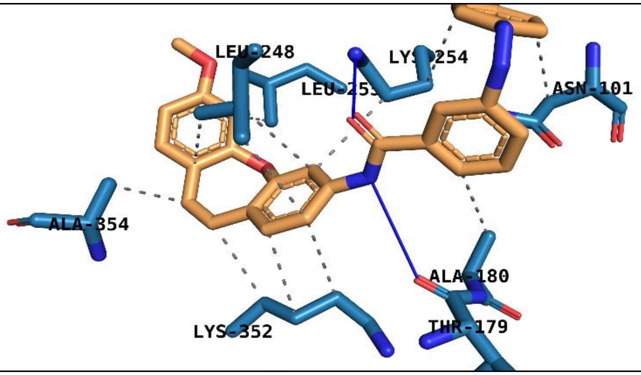	Hydrophobic Interactions	Asn101<br>Ala180<br>Leu248<br>Lys254<br>Leu255<br>Ala354
		Hydrogen Bonds	Thr179<br>Lys254
**5g*E***	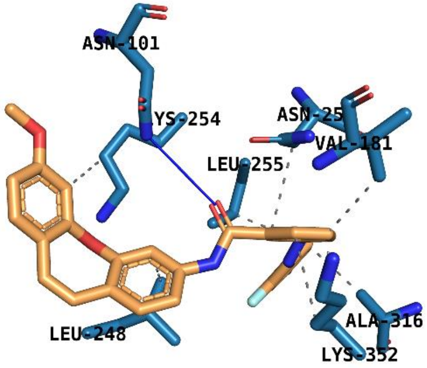	Hydrophobic Interactions	Val181<br>Leu248<br>Lys254<br>Leu255<br>Asn258<br>Ala316<br>Lys353
		Hydrogen Bonds	Asn101
**5g*Z***	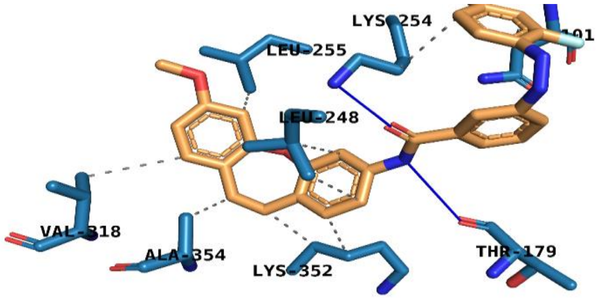	Hydrophobic Interactions	Asn101<br>Leu248<br>Lys254<br>Leu255<br>Val318<br>Lys352<br>Ala354
		Hydrogen Bonds	Thr179<br>Lys254

**5h*E***	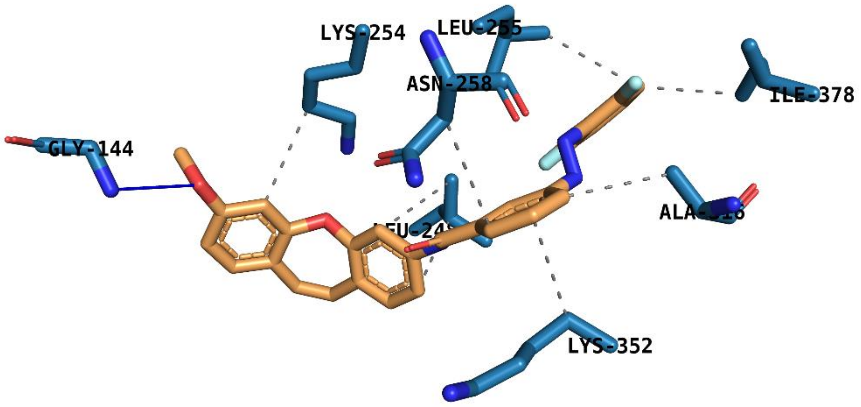	Hydrophobic Interactions	Leu248<br>Lys254<br>Leu255<br>Asn258<br>Ala316<br>Lys352<br>Ile378
		Hydrogen Bonds	Gly144
**5h*Z***	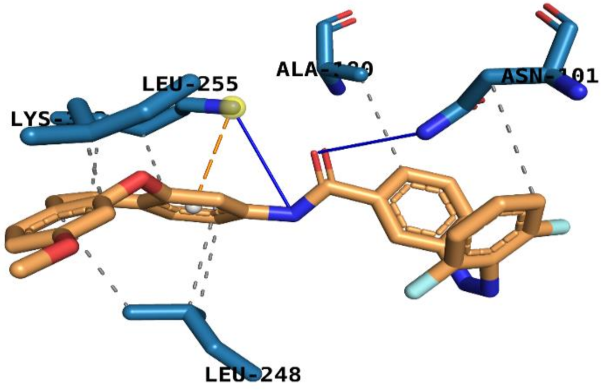	Hydrophobic Interactions	Asn101<br>Ala180<br>Leu248<br>Leu255<br>Lys252
		Hydrogen Bonds	Asn101<br>Lys352
		π-Cation Interactions	Lys352

Table 3S. Estimated binding energy (kJ/mol) to tubulin of (4a-4h) and (5a-5h) isomers *E* and *Z*.

compound	affinity [kJ/mol]	Δaff (*E*-*Z*) [kJ/mol]	compound	affinity [kJ/mol]	Δaff (E-Z) [kJ/mol]
**4a*E***	-45.6	0	**5a*E***	-51.9	-2.5
**4a*Z***	-45.6		**5a*Z***	-49.4	
**4b*E***	-47.3	3.8	**5b*E***	-53.2	-0.4
**4b*Z***	-51.1		**5b*Z***	-52.8	
**4c*E***	-47.3	1.7	**5c*E***	-49.0	4.6
**4c*Z***	-49.0		**5c*Z***	-53.6	
**4d*E***	-49.4	-1.7	**5d*E***	-52.8	-2.1
**4d*Z***	-47.7		**5d*Z***	-50.7	
**4e*E***	-41.0	6.7	**5e*E***	-49.8	-0.8
**4e*Z***	-47.7		**5e*Z***	-49.0	
**4f*E***	-43.5	8.4	**5f*E***	-54.0	-1.7
**4f*Z***	-51.9		**5f*Z***	-52.3	
**4g*E***	-46.5	6.3	**5g*E***	-51.1	1.2
**4g*Z***	-52.8		**5g*Z***	-52.3	
**4h*E***	-46.9	1.2	**5h*E***	-49.8	5
**4h*Z***	-48.1		**5h*Z***	-54.8	

2. Experimental section

2.1. Nuclear magnetic resonance (NMR) spectroscopy:

All the spectra were recorded using a Varian VNMRS spectrometer operating at 11.7 T and Varian Mercury VX 9.4 T magnetic field. Measurements were performed for ca. 1.0 M solutions of all the compounds in DMSO-d_6 or $CDCl_3$. The residual signals of DMSO-d6 (2.54 ppm) and $CDCl_3$ (7.26 ppm) in ^{1}H NMR and of the DMSO-d_6 signal (40.45 ppm) and of $CDCl_3$ (77.0 ppm) in ^{13}C NMR spectra were used as the chemical shift references. Spin multiplicities are described as s (singlet), d(doublet), t (triplet), q (quartet), m (multiplet), dd (double doublet). Coupling constants are reported in Hertz. All the proton spectra were recorded using the standard spectrometer software and parameters set: acquisition time 3s, pulse angle 30°. The standard measurement parameter set for ^{13}C NMR spectra was: pulse width 7 μs (the 90° pulse width was 12.5 μs), acquisition time 1 s, spectral width 200 ppm, 1000 scans of 32 K data point were accumulated and after zero-filling to 64 K; and the FID signals were subjected to Fourier transformation after applying a 1 Hz line broadening. The ^{1}H-^{13}Cgs-HSQC and ^{1}H-^{13}Cgs-HMBC spectra were also recorded using the standard Varian software.

Measurement of NOE effect- ^{13}C NMR spectra with ^{1}H WALTZ decoupling. The use during acquisition decoupling - is the continuous wave (cw) irradiation of one of the proton *ortho* of dibenzo [*b, f*] oxepine (**4h*E***) in the ring closer to the carbonyl group at a single decoupling frequency and observation effect on ^{13}C spectrum. And next measurement of the spectrum without irradiation of proton (d1=8s, at=1 s) and observation of ^{13}C spectrum.

In order to determine the ***E* / *Z*** isomer ratio of **5h**, ^{1}H NMR spectra were recorded after the NMR tube was irradiated with light with a wavelength λ = 525 for 10 min and 60 min.

2.2. Mass spectrometry (MS): Mass spectra were recorded on spectrometer QTOF Premier firm Waters and spectrometer LTQ Orbitrap Velos.

2.3. Photoisomerization studies by UV-VIS spectroscopy: EnSpire® multimode plate reader (PerkinElmer, Turku, Finland) with the software EnSpire Workstation version 4.10.3005.1440 (PerkinElmer) in absorbance mode was used for UV-VIS spectroscopic measurements. All experiments were done in at least triplicate. Stock solutions of selected compounds were made in DMSO at appropriate concentrations and stirred at 150°C overnight to ensure complete relaxation of *E* – isomer. Next 100 μL of stock solution was transferred to 96 – a well plate as quickly as possible and absorbance was read. Afterward, samples were illuminated with light at λ = 525 nm by a self-made LED setup consisting of three high-power 1W LED (ASMT-AG00-NST00) in series. The working setup is presented in Figure 1S. Samples were illuminated with increasing periods until no further spectrum changes were observed.

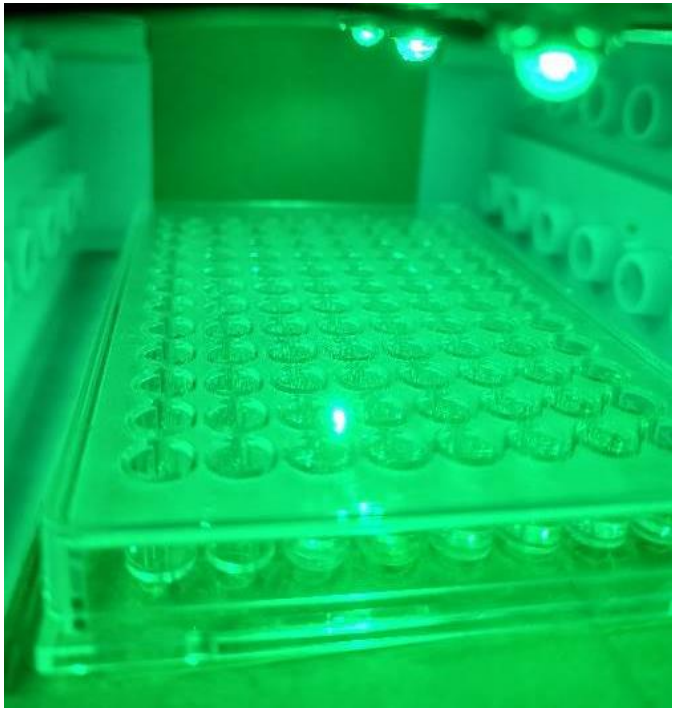

Figure 1S. Self-made 3W green LED setup illuminating samples in 96-well plate.

50μM and 500μM solutions in DMSO have been used for photoswitching studies of azobenzene by $UV - vis$ spectroscopy [1]

[1] D. B. Konrad, ,G. Savasci, L. Allmendinger, D. Trauner, C. Ochsenfeld, A. M. AliJ., *Am. Chem. Soc.* 2020, **142**, 6538 − 6547

3. NMR spectra of obtained compounds, yield, time of reaction, melting point, and high resolution mass date.

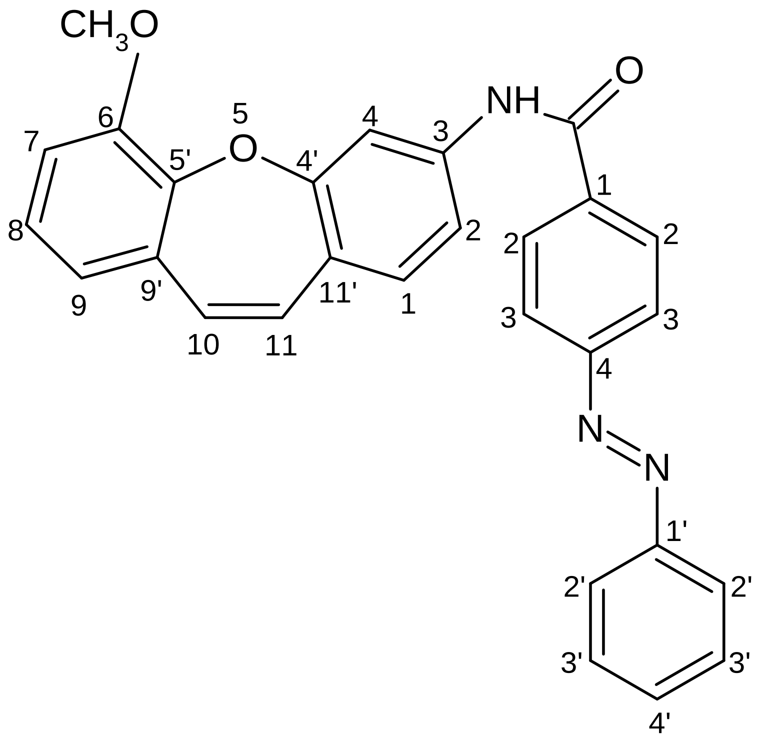

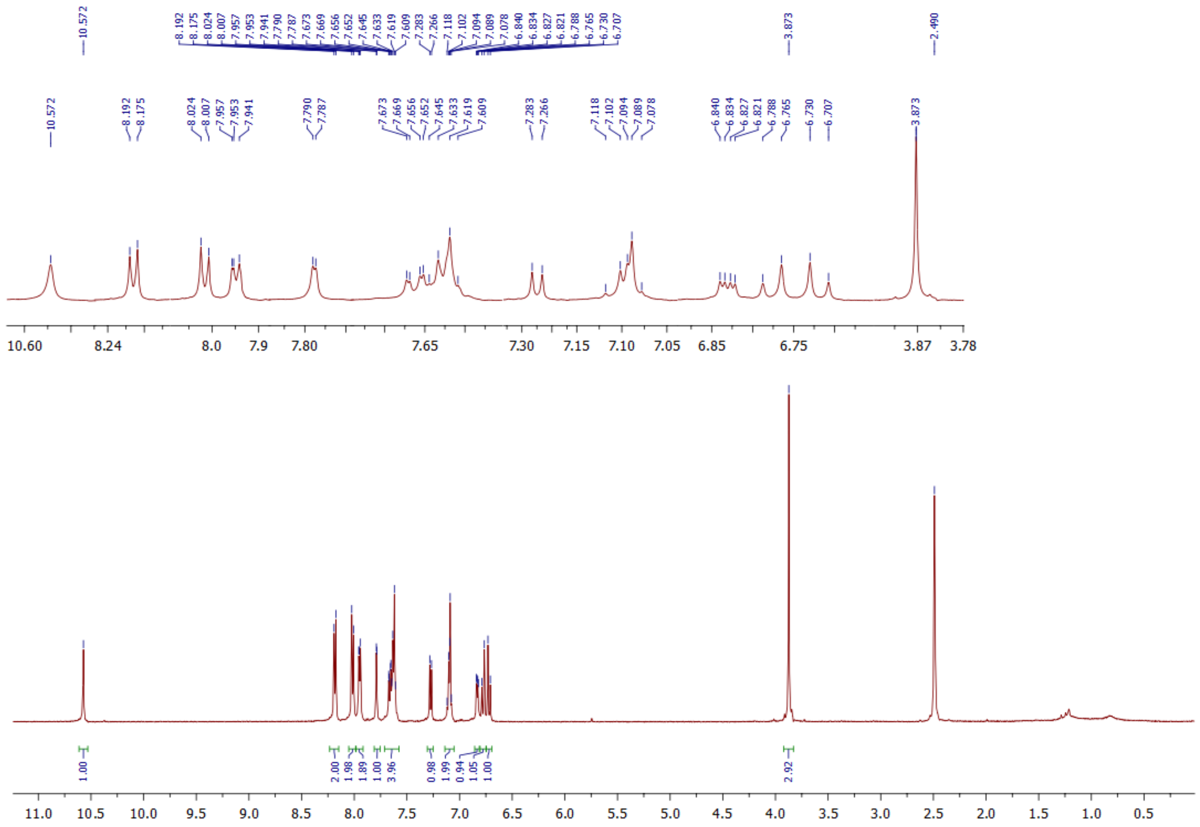

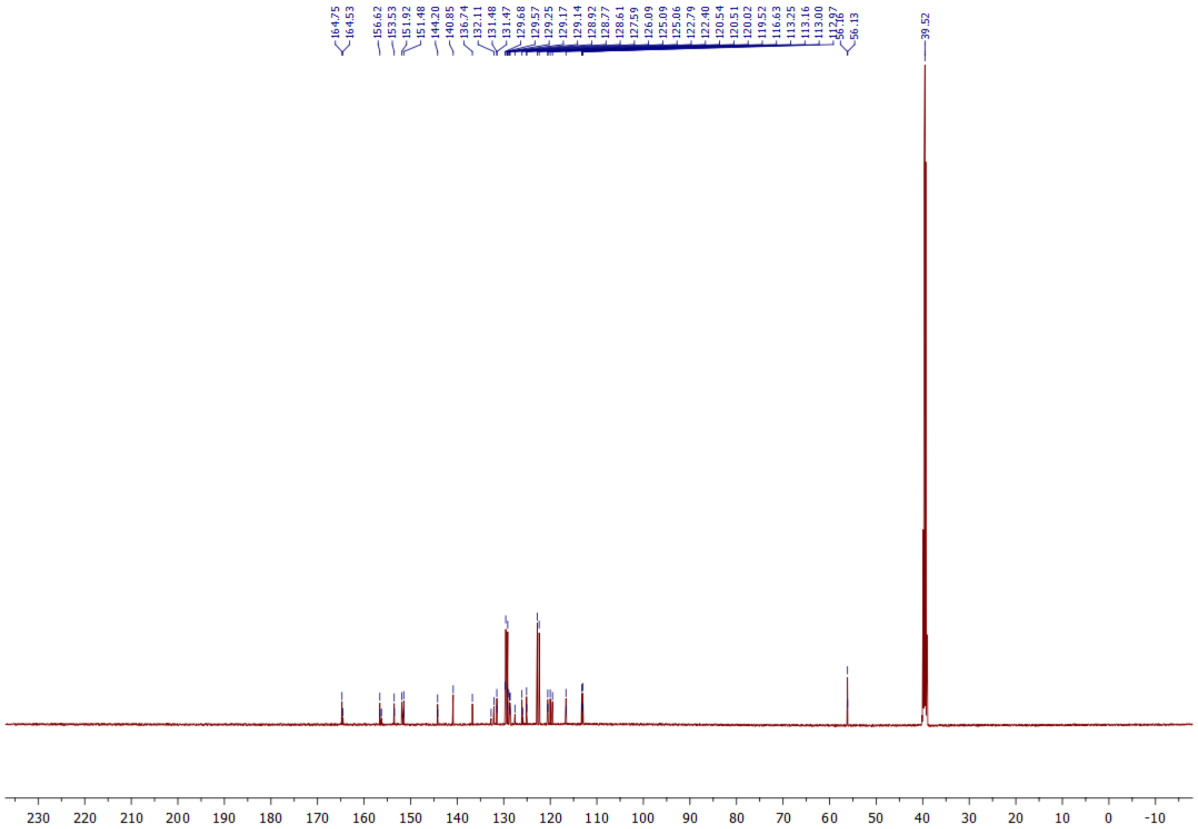
164.75
164.53
156.62
153.53
151.92
151.48
144.20
140.85
136.74
132.11
131.48
131.47
129.68
129.57
129.25
129.17
129.14
128.92
128.77
128.61
127.59
126.09
125.09
125.06
122.79
122.40
120.54
120.51
120.02
119.52
116.63
113.25
113.16
113.00
112.97
56.16
56.13
39.52
230
220
210
200
190
180
170
160
150
140
130
120
110
100
90
80
70
60
50
40
30
20
10
0
-10

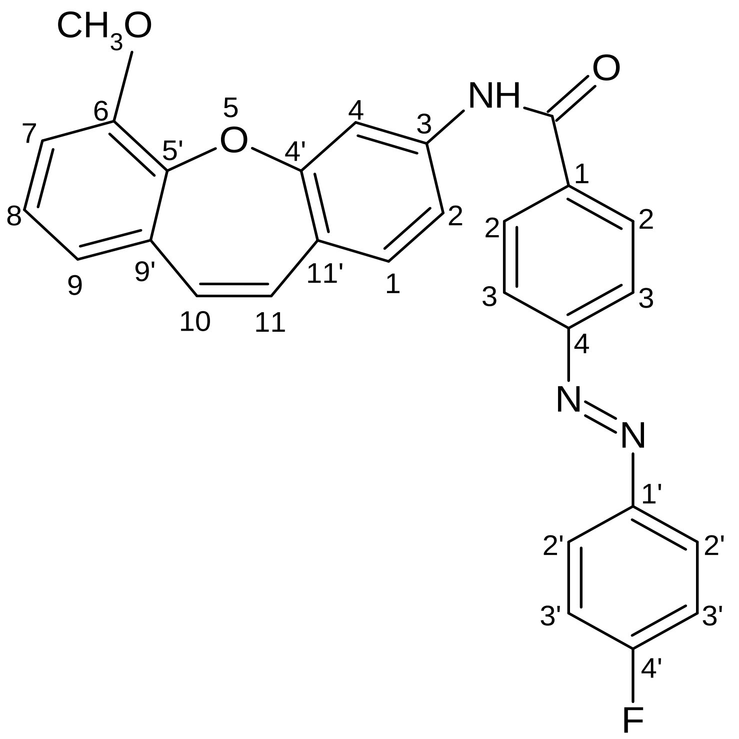
CH3O
O
NH
O
N
N
F
5
6
7
8
9
9'
10
11
11'
5'
4'
4
3
2
1
1
2
2
3
3
4
1'
2'
2'
3'
3'
4'

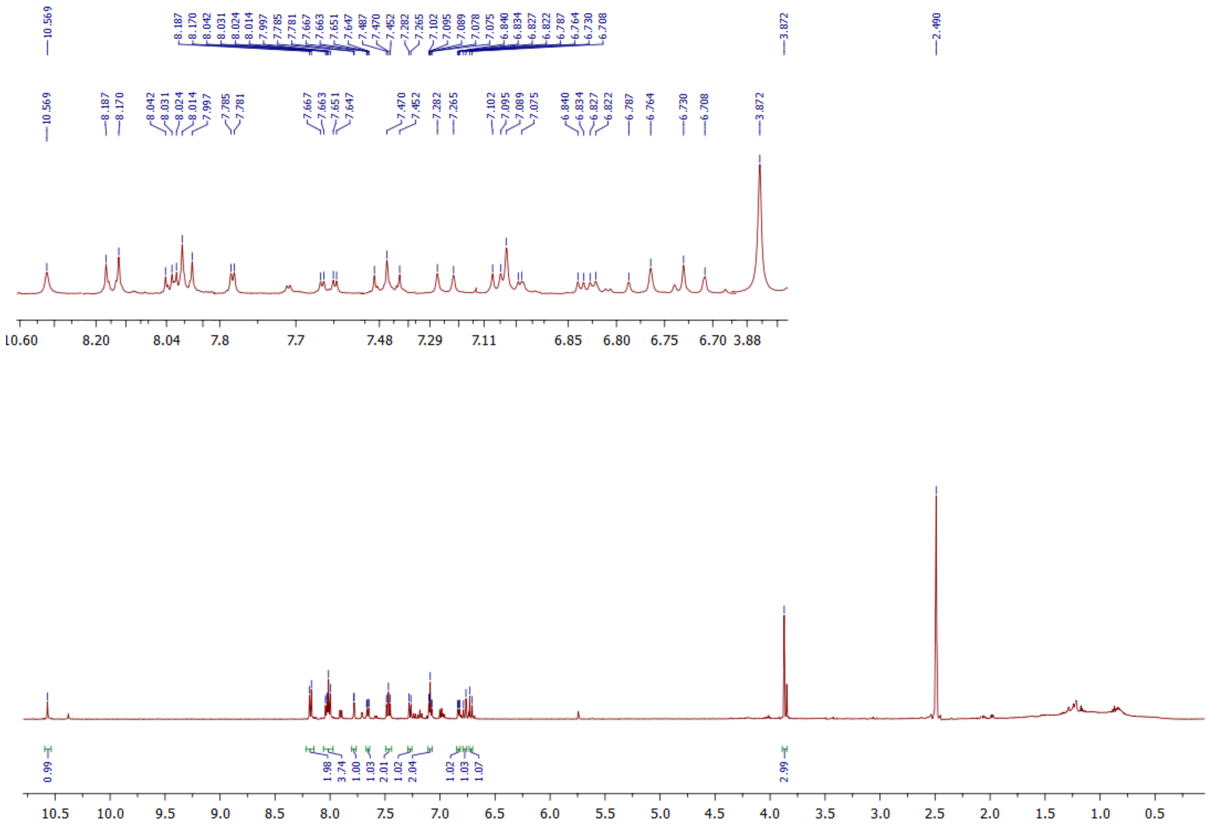

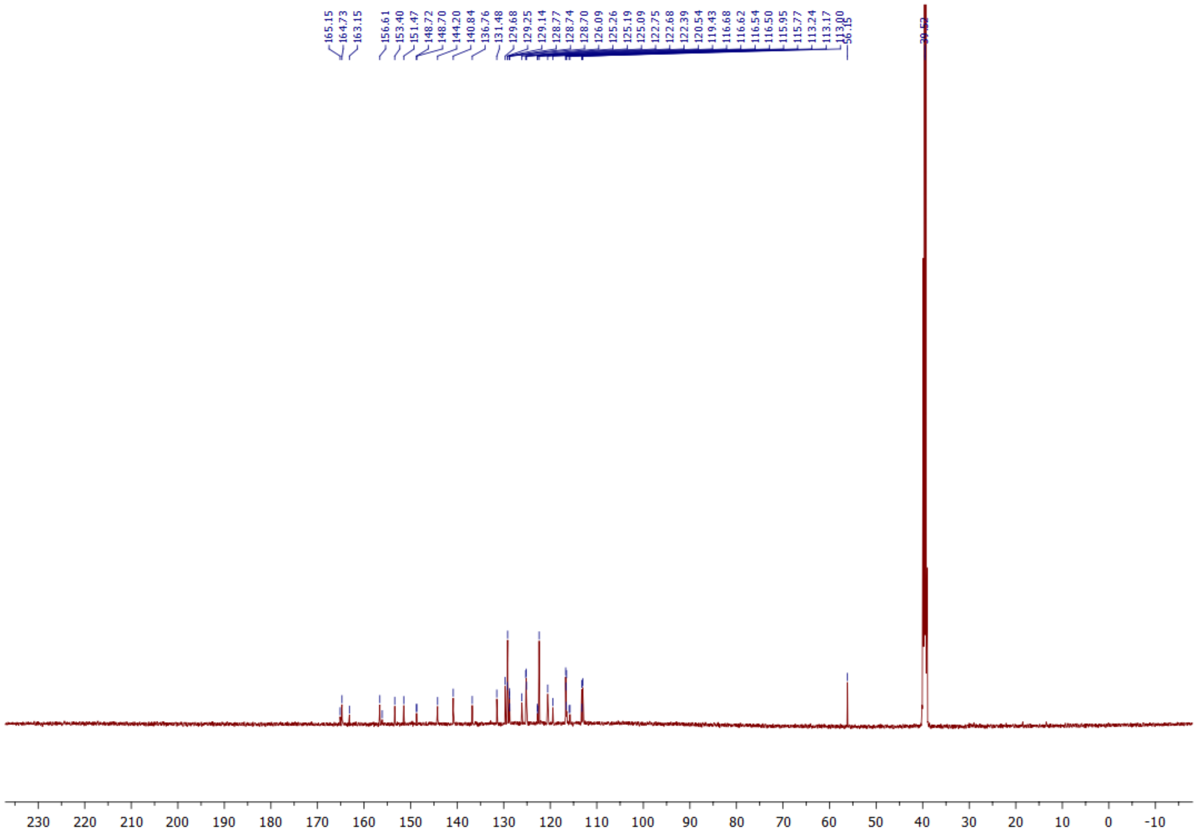
165.15
164.73
163.15
156.61
153.40
151.47
148.72
148.70
144.20
140.84
136.76
131.48
129.68
129.25
129.14
128.77
128.74
128.70
126.09
125.26
125.19
125.09
122.75
122.68
122.39
120.54
119.43
116.68
116.62
116.54
116.50
115.95
115.77
113.24
113.17
113.00
56.15
39.52
230 220 210 200 190 180 170 160 150 140 130 120 110 100 90 80 70 60 50 40 30 20 10 0 -10

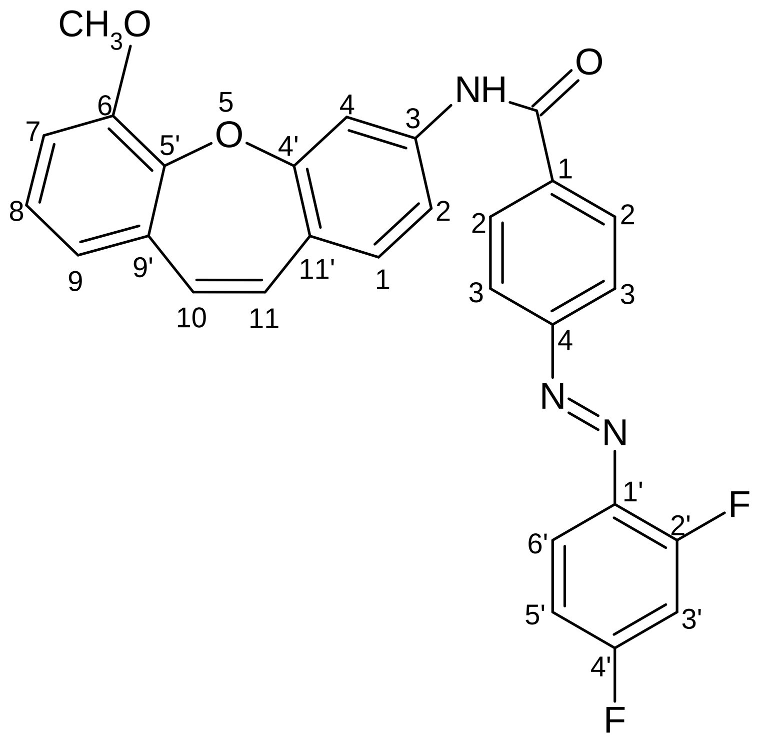
CH3O
O
NH
O
N
N
F
F

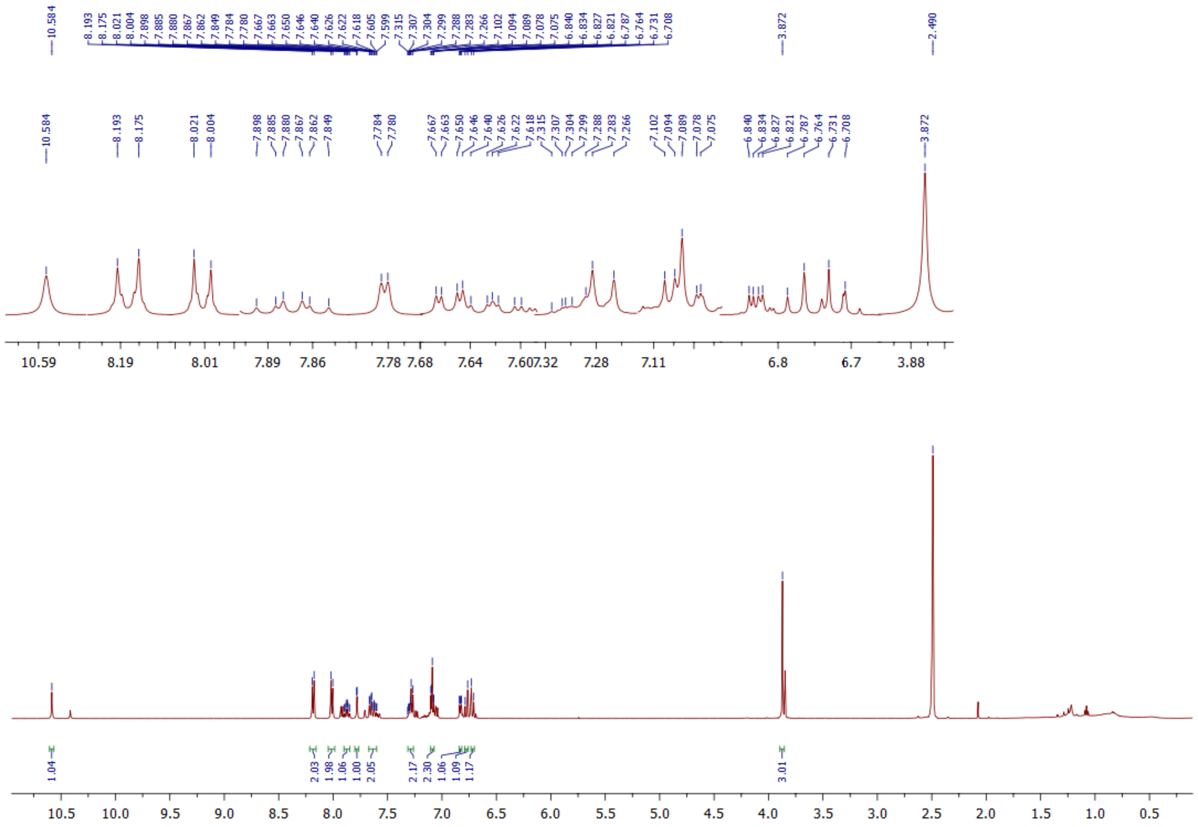

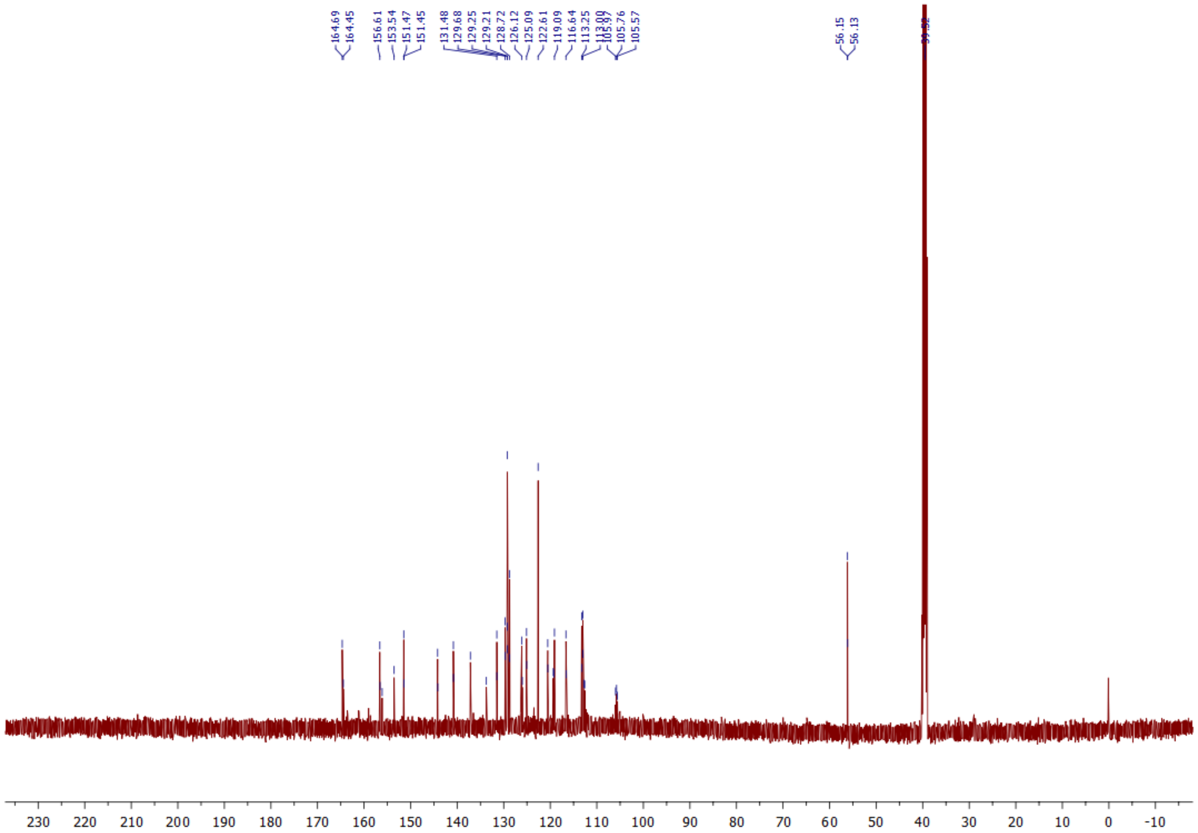
164.69
164.45
156.61
153.54
151.47
151.45
131.48
129.68
129.25
129.21
128.72
126.12
125.09
122.61
119.09
116.64
113.25
113.09
105.97
105.76
105.57
56.15
56.13
230
220
210
200
190
180
170
160
150
140
130
120
110
100
90
80
70
60
50
40
30
20
10
0
-10

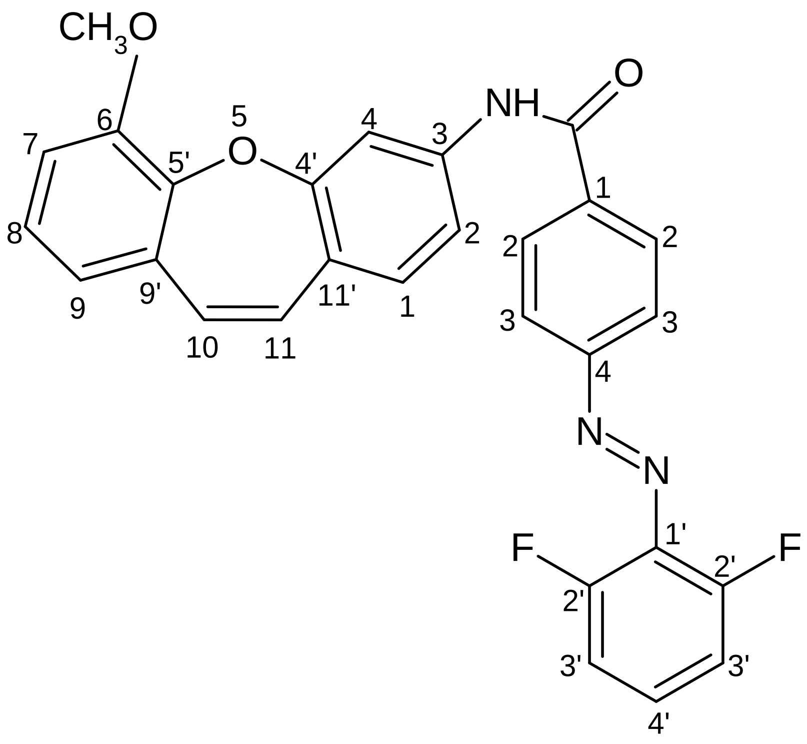
CH3O
5
O
6
7
5'
4'
4
3
NH
O
1
2
2
8
9
9'
10
11
11'
1
3
3
4
N
N
1'
F
F
2'
2'
3'
3'
4'

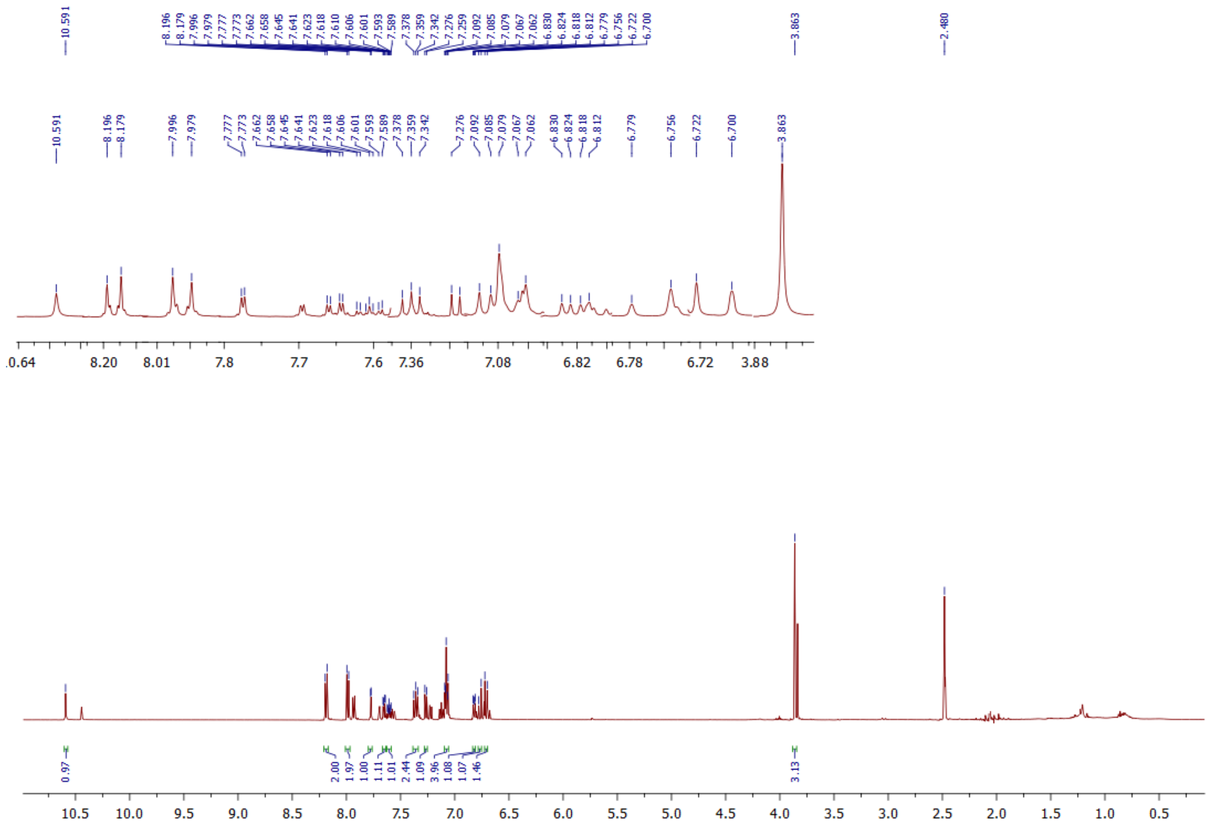

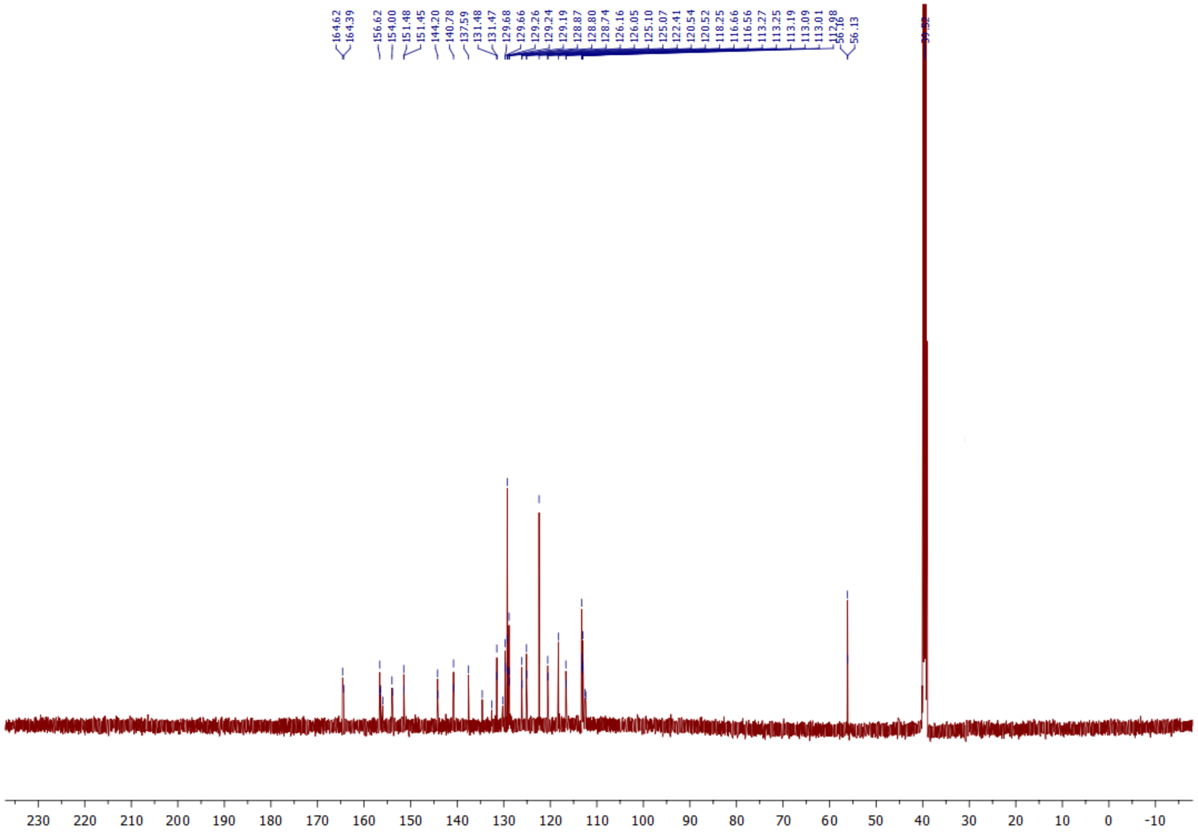
164.62
164.39
156.62
154.00
151.48
151.45
144.20
140.78
137.59
131.48
131.47
129.68
129.66
129.26
129.24
129.19
128.87
128.80
128.74
126.16
126.05
125.10
125.07
122.41
120.54
120.52
118.25
116.66
116.56
113.27
113.25
113.19
113.09
113.01
112.98
56.16
56.13
39.52
230
220
210
200
190
180
170
160
150
140
130
120
110
100
90
80
70
60
50
40
30
20
10
0
-10

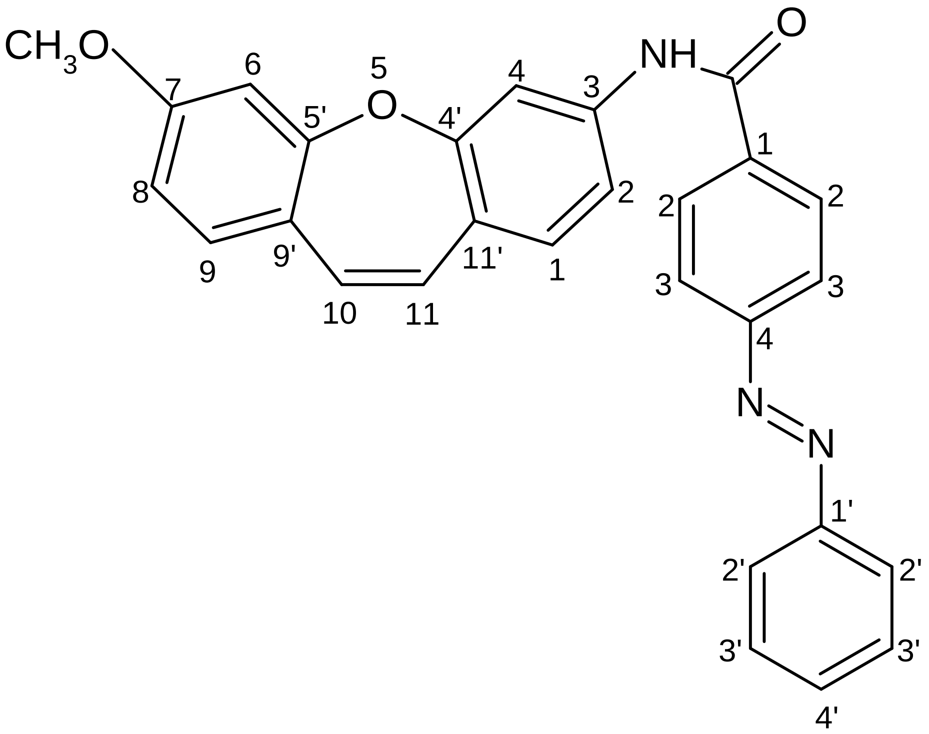
CH3O
7
6
5
O
5'
4'
4
3
NH
O
1
2
8
9'
9
10
11
11'
1
2
2
2
3
3
4
N
N
1'
2'
2'
3'
3'
4'

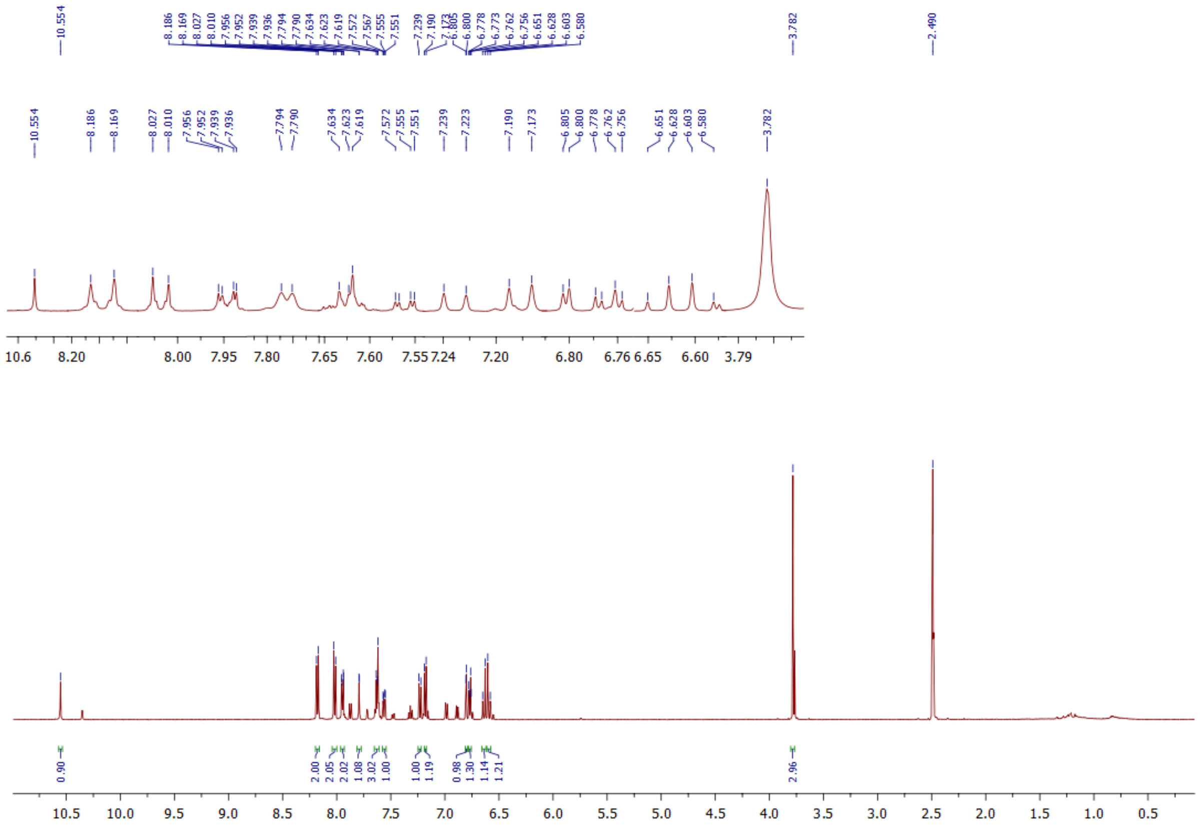

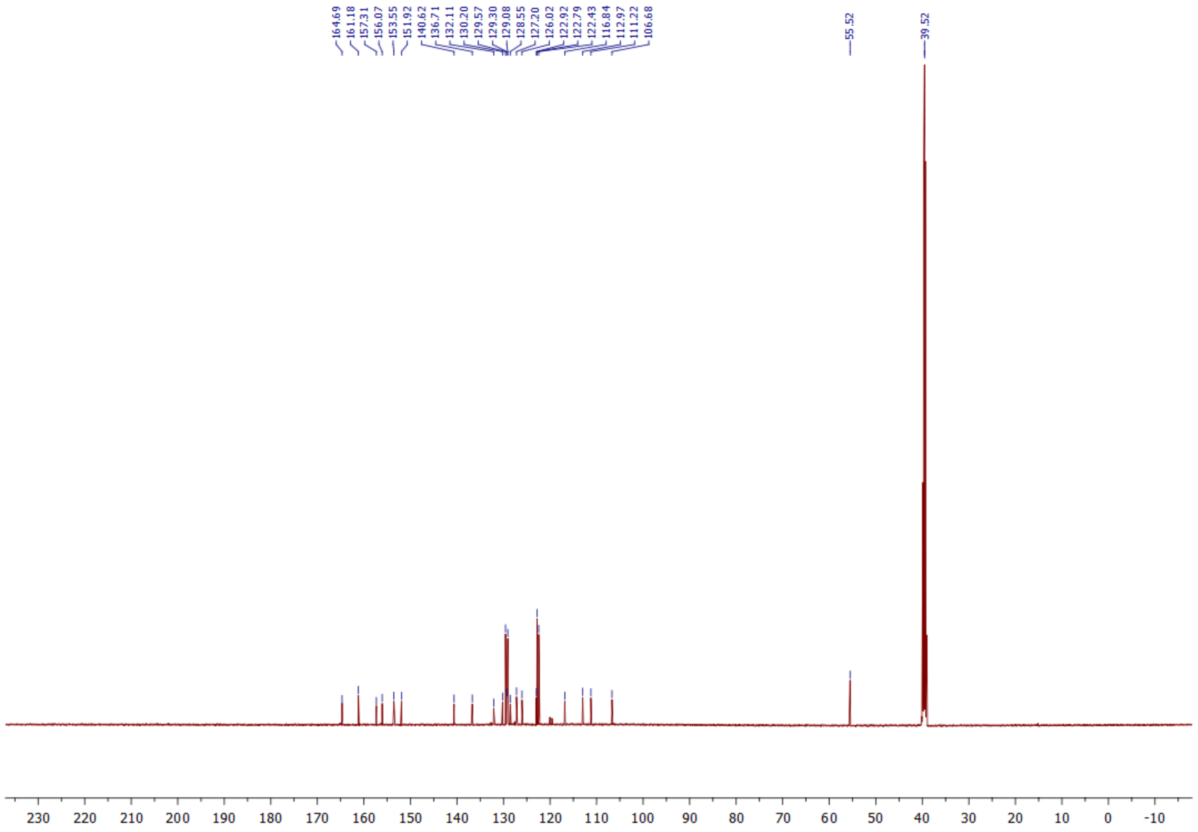
164.69
161.18
157.31
156.07
153.55
151.92
140.62
136.71
132.11
130.20
129.57
129.30
129.08
128.55
127.20
126.02
122.92
122.79
122.43
116.84
112.97
111.22
106.68
55.52
39.52
230 220 210 200 190 180 170 160 150 140 130 120 110 100 90 80 70 60 50 40 30 20 10 0 -10

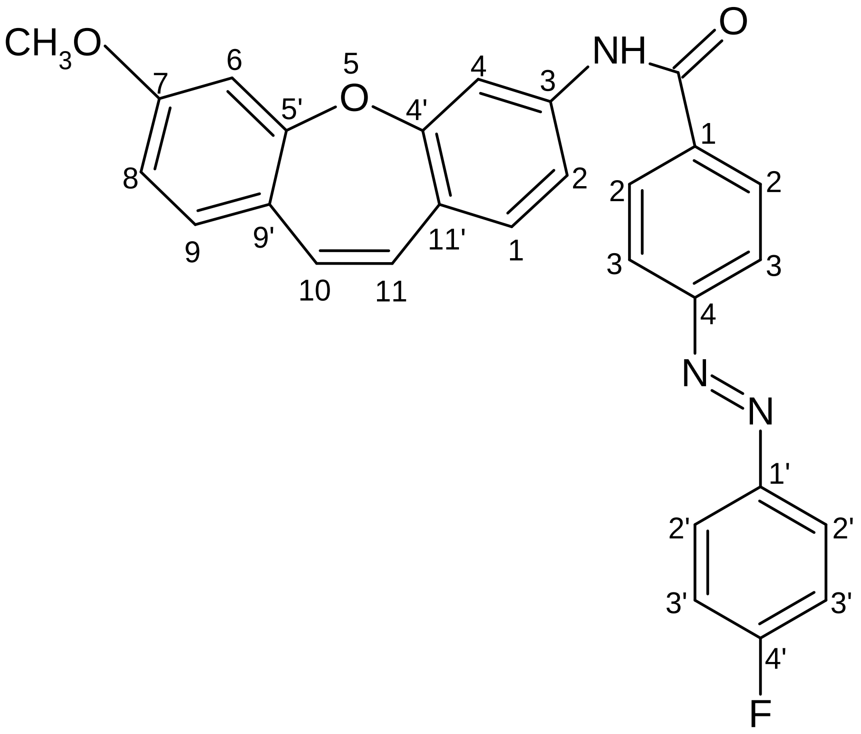

CH3O
7
6
5
O
5'
4'
4
3
NH
O
8
9
9'
10
11
11'
1
2
1
2
2
3
3
4
N
N
1'
2'
2'
3'
3'
4'
F

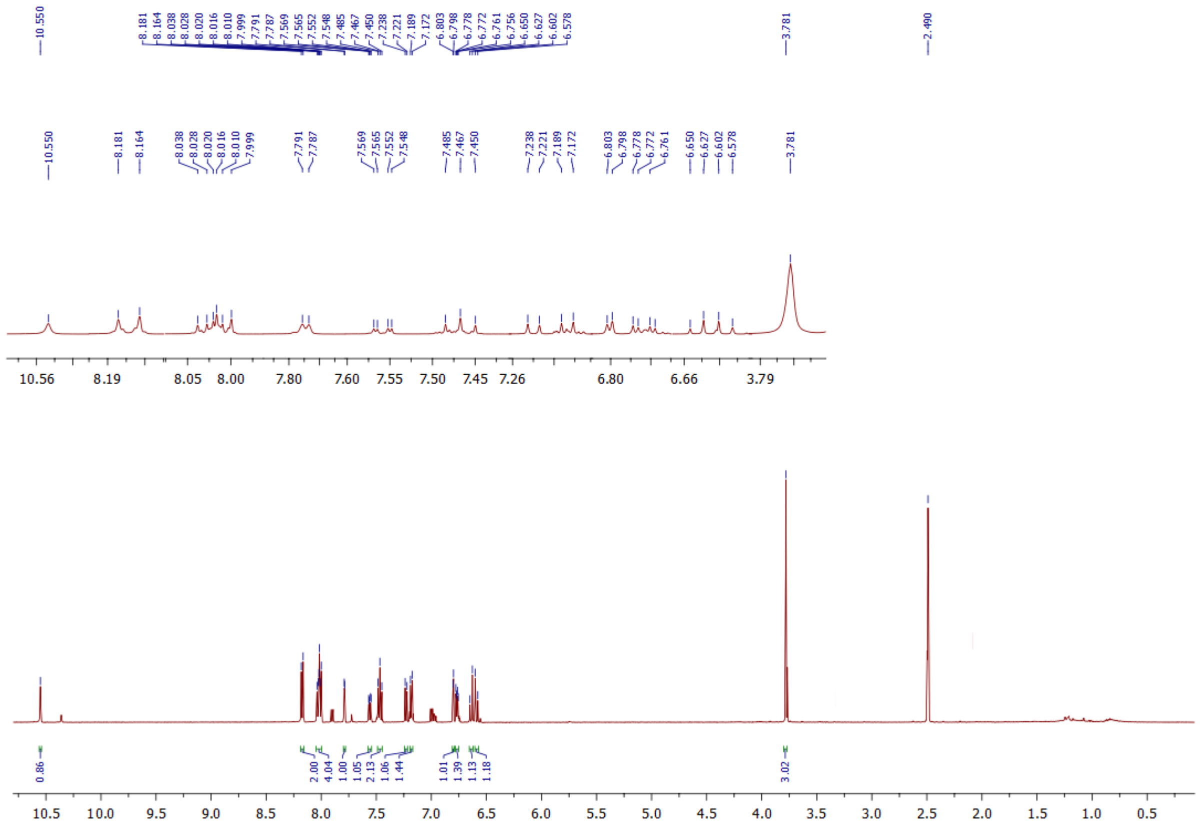

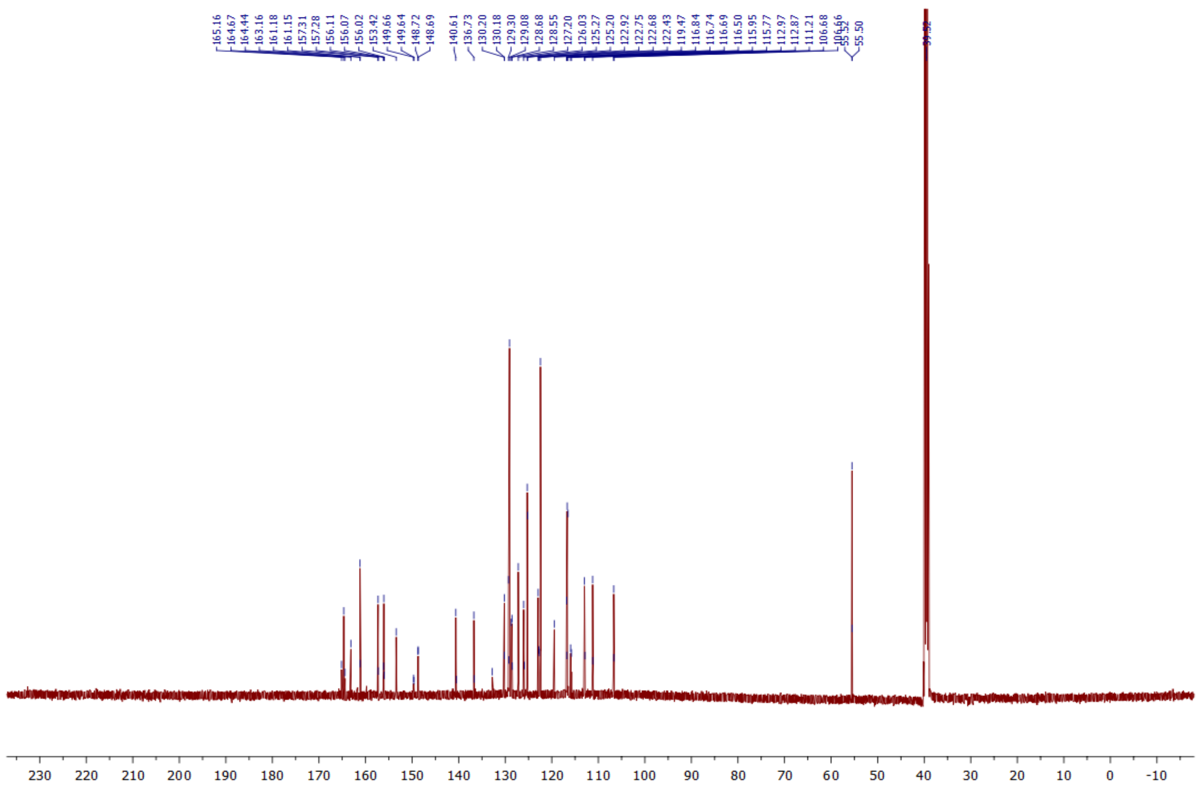

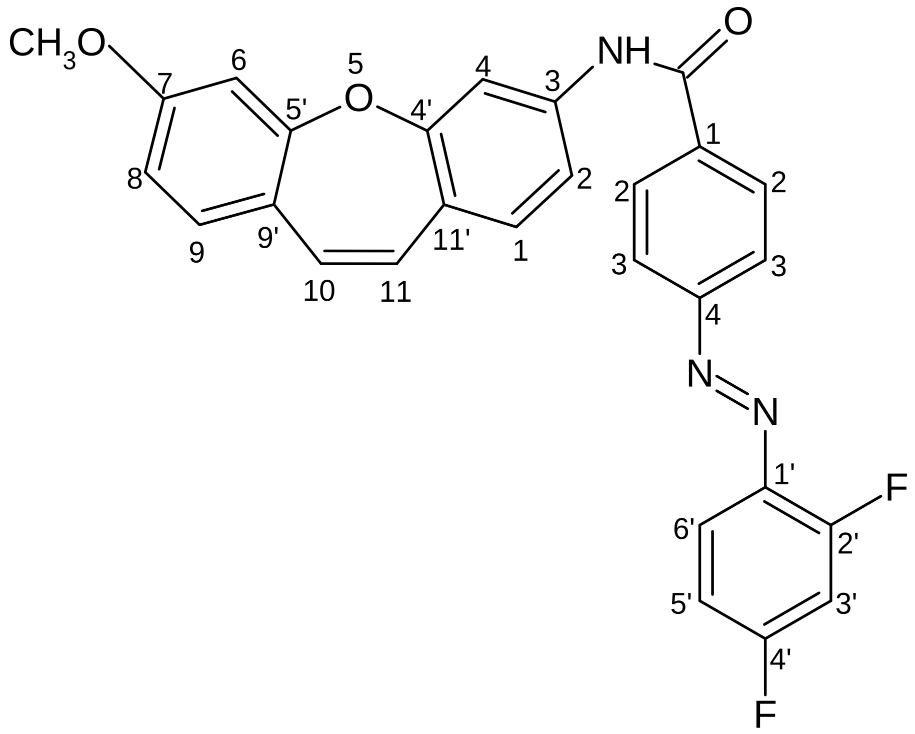
CH3O
7
6
5
O
5'
4'
4
3
NH
O
8
9
9'
10
11
11'
1
2
1
2
2
3
3
4
N
N
1'
F
6'
2'
5'
3'
4'
F

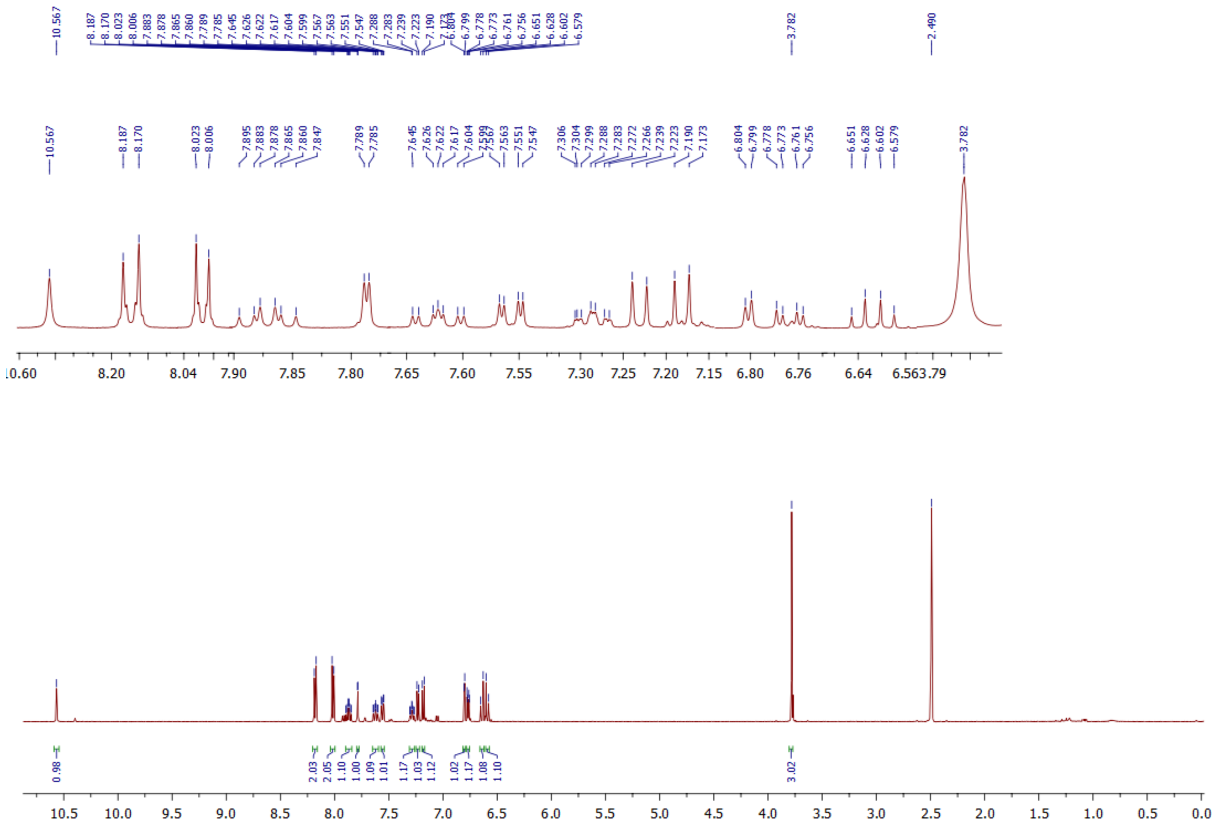

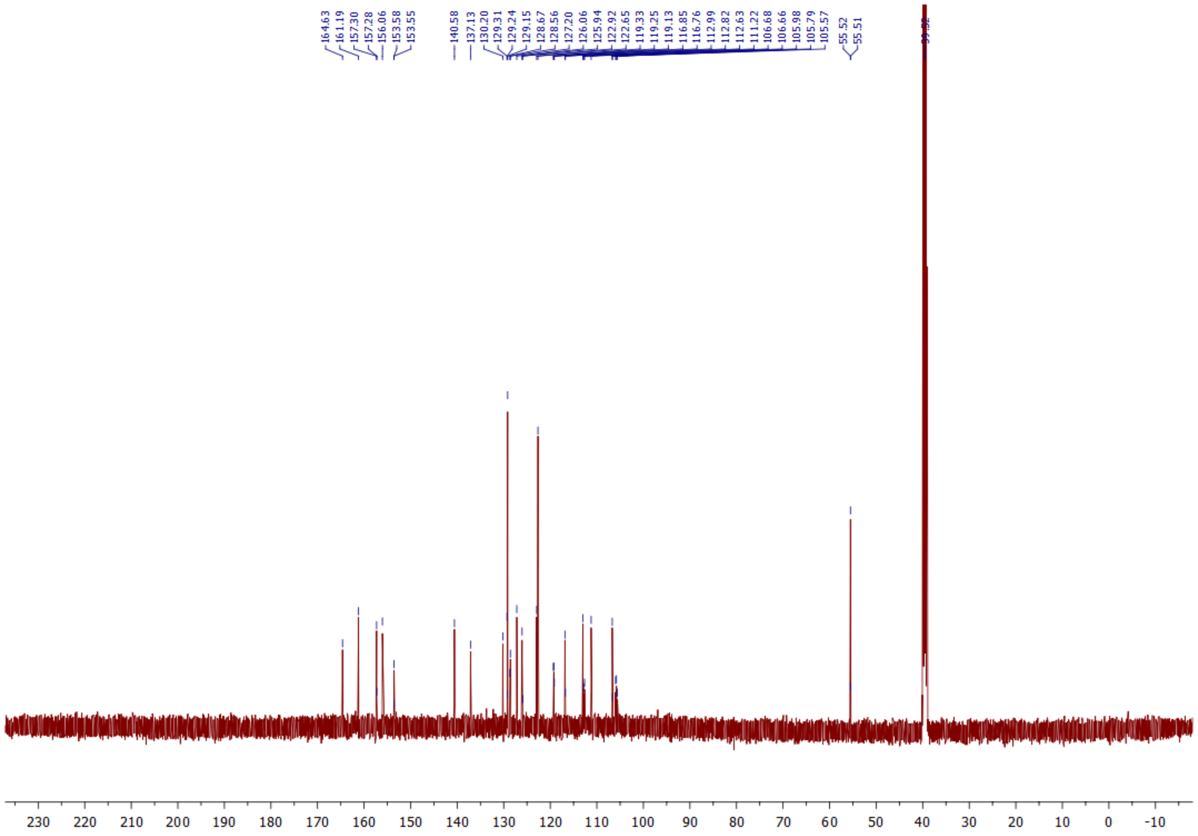
164.63
161.19
157.30
157.28
156.06
153.58
153.55
140.58
137.13
130.20
129.31
129.24
129.15
128.67
128.56
127.20
126.06
125.94
122.92
122.65
119.33
119.25
119.13
116.85
116.76
112.99
112.82
112.63
111.22
106.68
106.66
105.98
105.79
105.57
55.52
55.51
230 220 210 200 190 180 170 160 150 140 130 120 110 100 90 80 70 60 50 40 30 20 10 0 -10

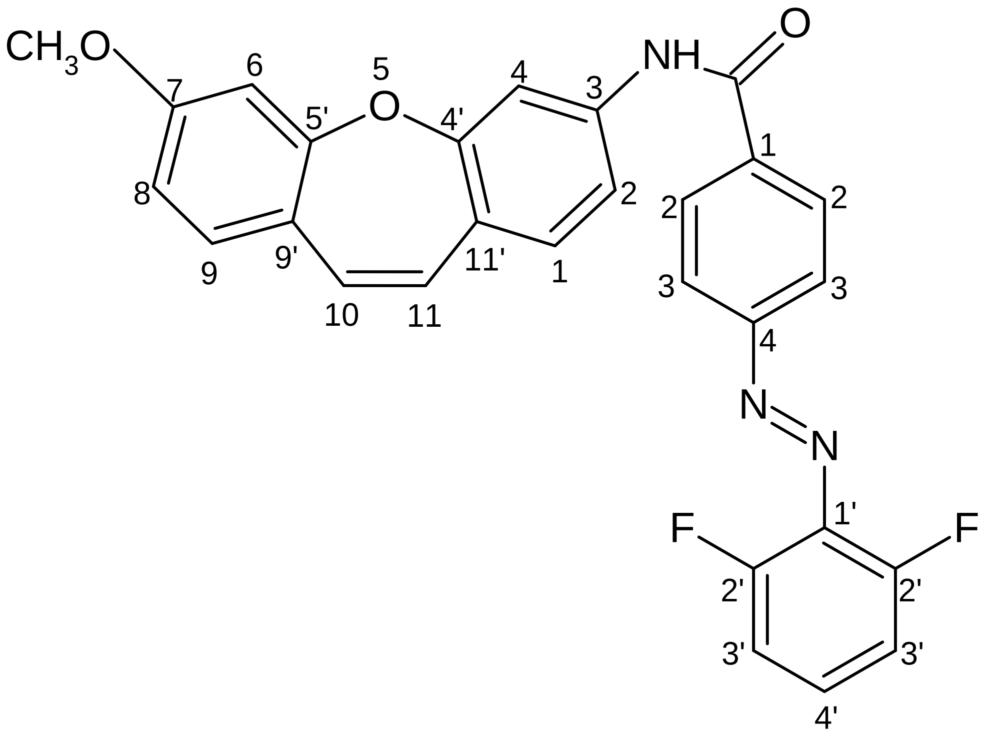
CH3O
7
6
5
O
5'
4'
4
3
NH
O
1
2
8
9
9'
10
11
11'
1
2
2
3
3
4
N
N
1'
F
F
2'
2'
3'
3'
4'

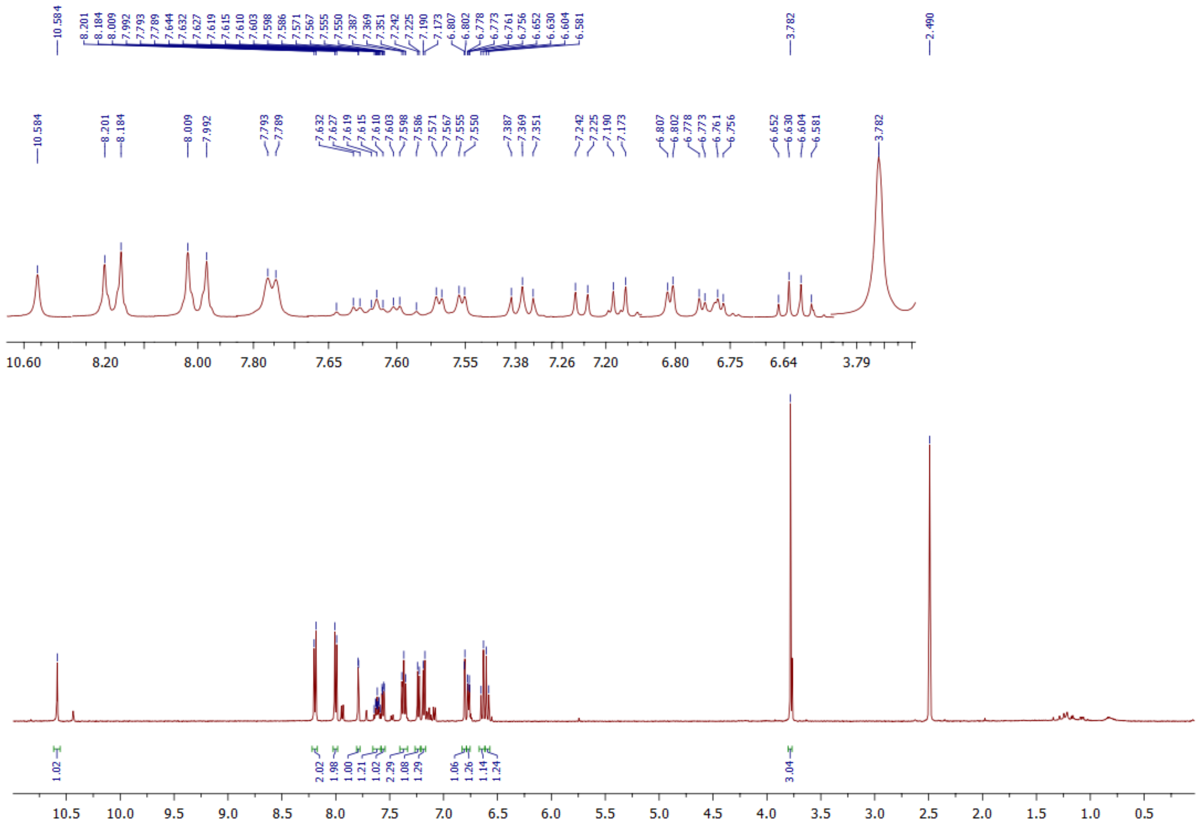

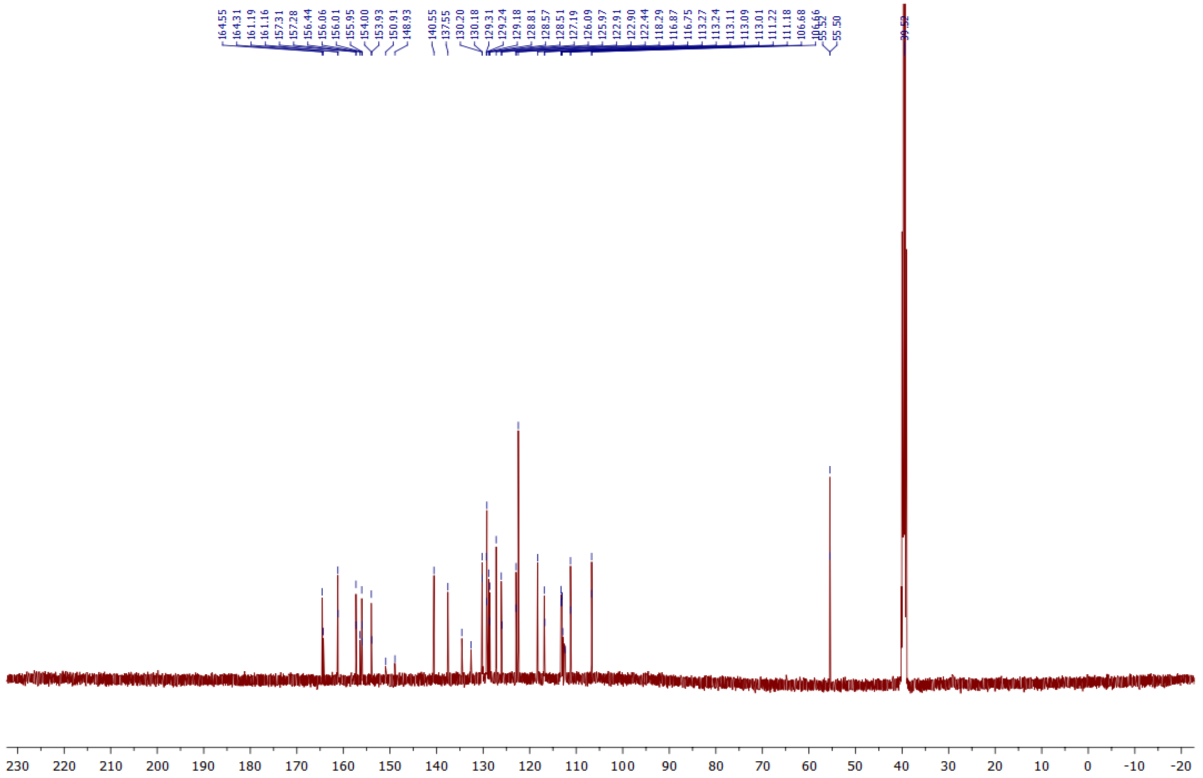

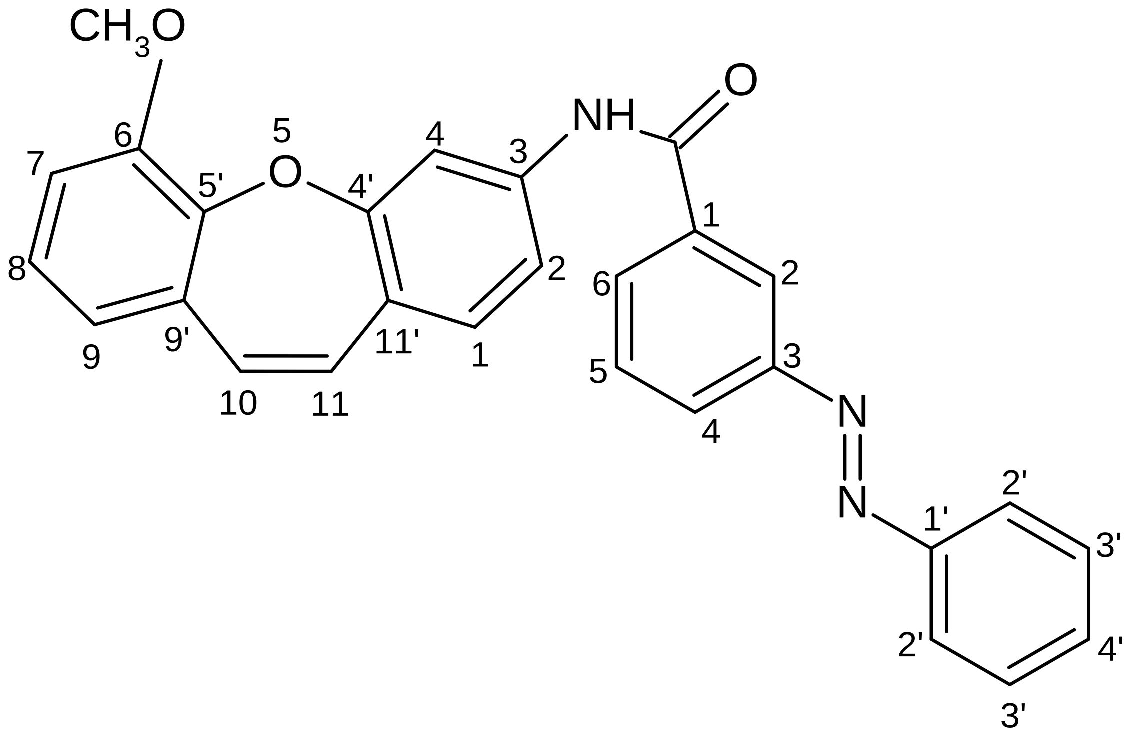

CH3O
6
7
5
O
5'
4'
4
3
NH
O
1
2
8
6
2
9'
11'
9
1
3
5
10
11
N
4
N
2'
1'
3'
2'
4'
3'

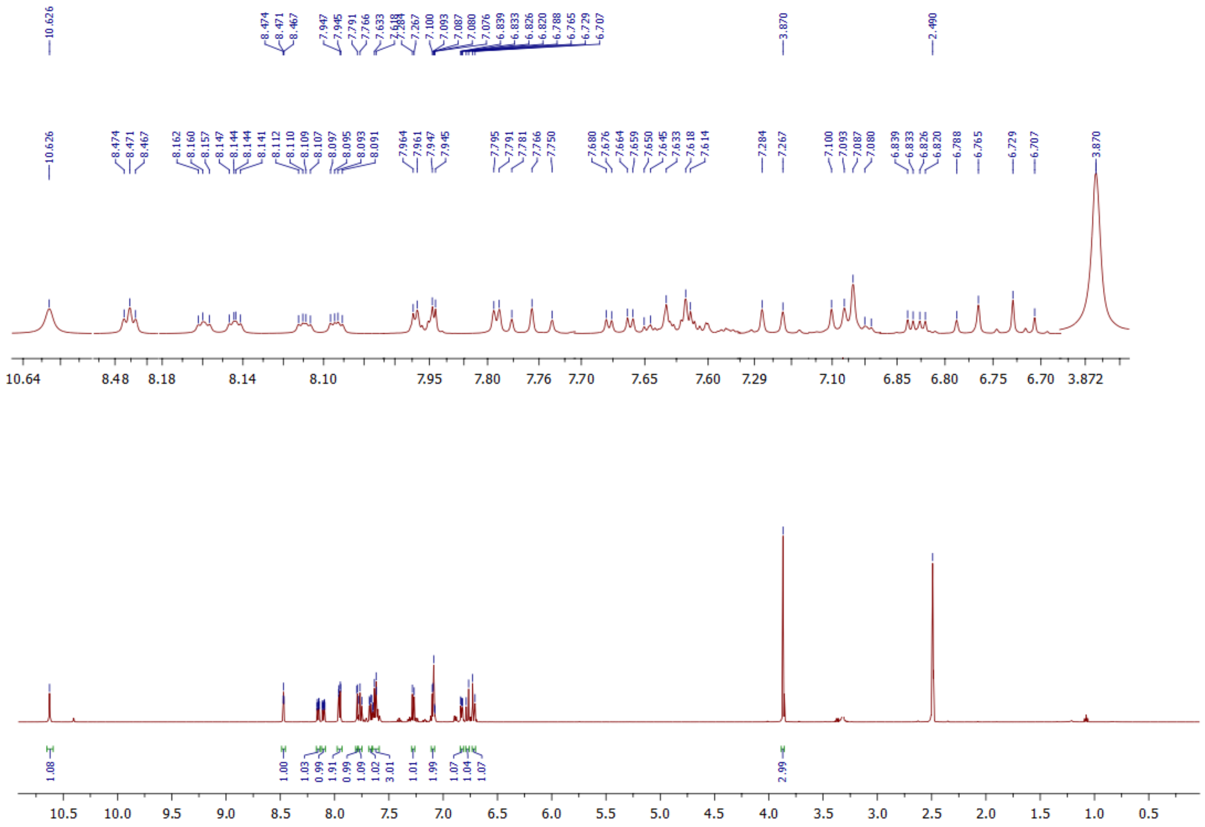

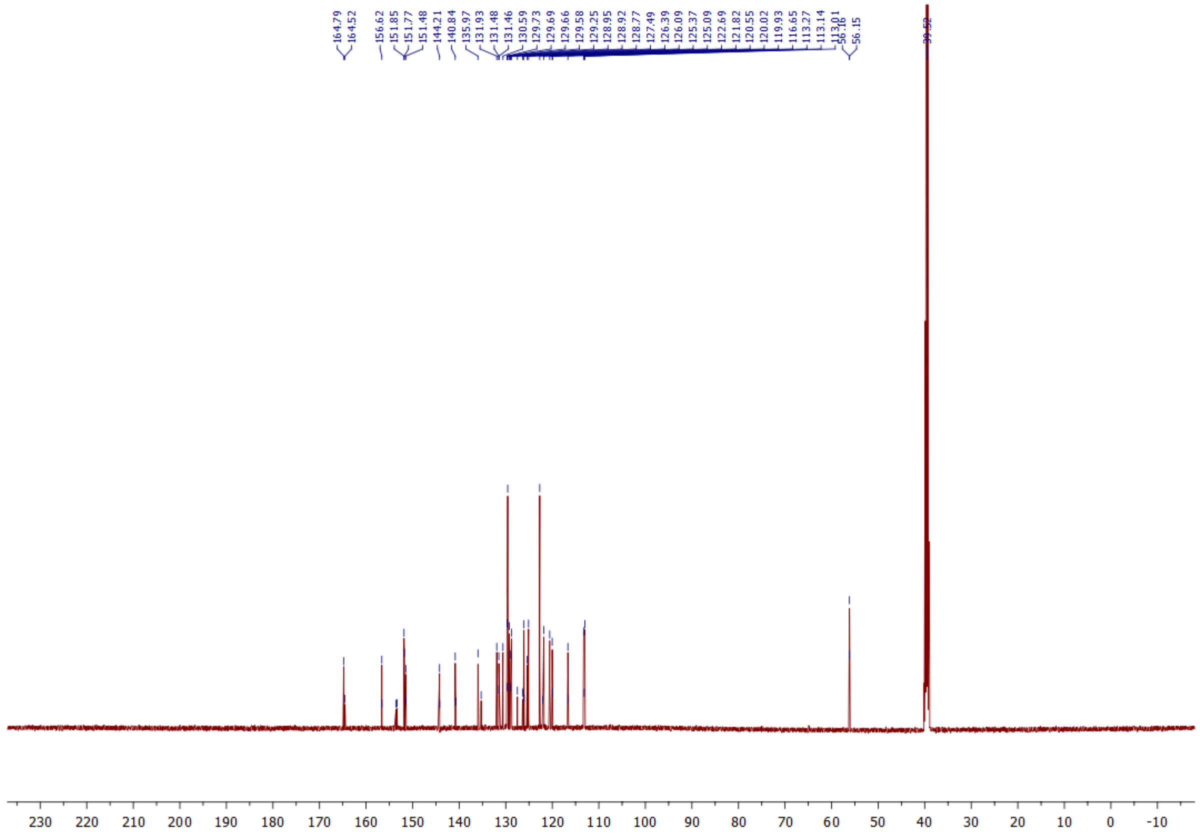
164.79
164.52
156.62
151.85
151.77
151.48
144.21
140.84
135.97
131.93
131.48
131.46
130.59
129.73
129.69
129.66
129.58
129.25
128.95
128.92
128.77
127.49
126.39
126.09
125.37
125.09
122.69
121.82
120.55
120.02
119.93
116.65
113.27
113.14
113.01
56.16
56.15
39.52
230 220 210 200 190 180 170 160 150 140 130 120 110 100 90 80 70 60 50 40 30 20 10 0 -10

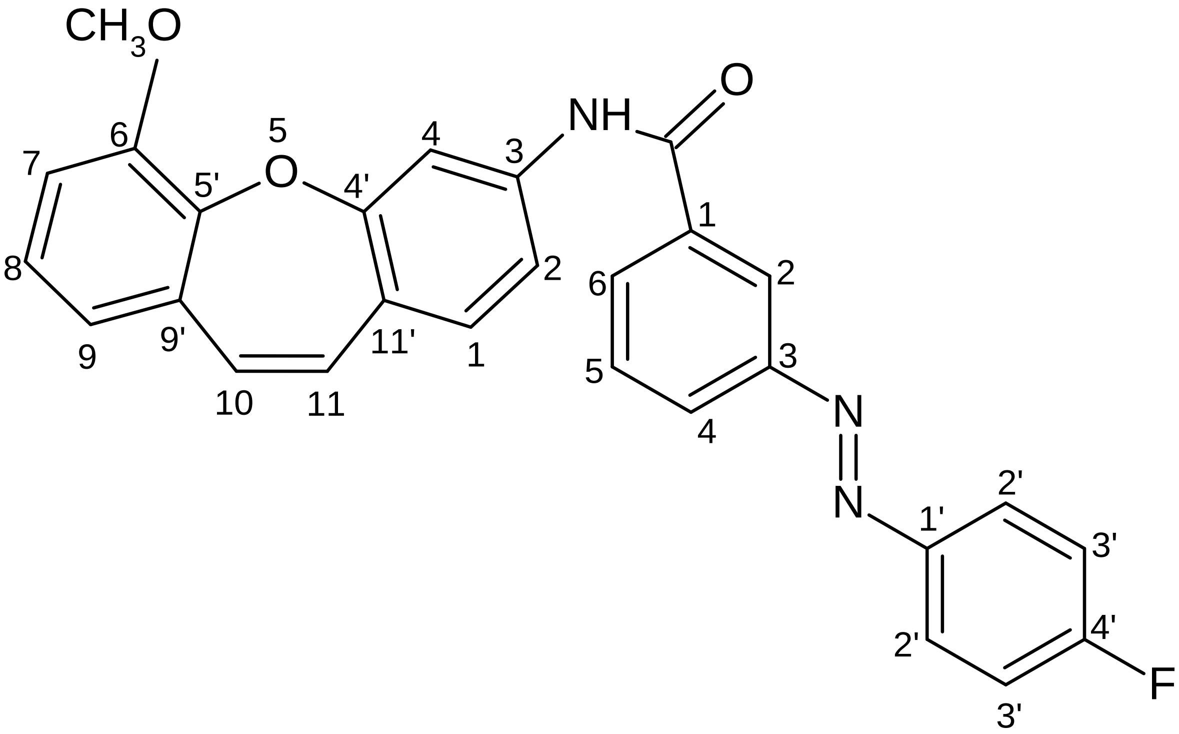
CH3O
5
O
6
7
5'
4'
4
3
NH
O
8
9
9'
10
11
11'
1
2
1
2
6
3
5
4
N
N
2'
1'
3'
2'
4'
3'
F

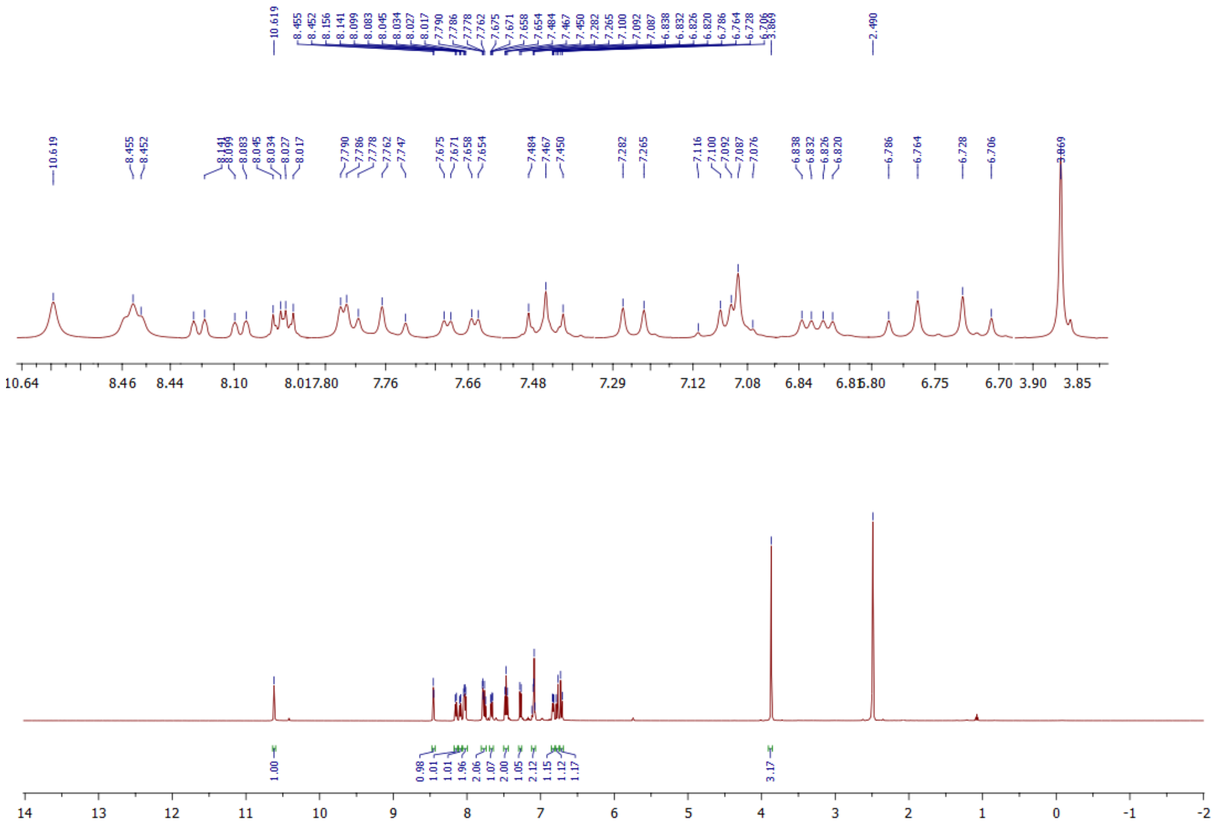

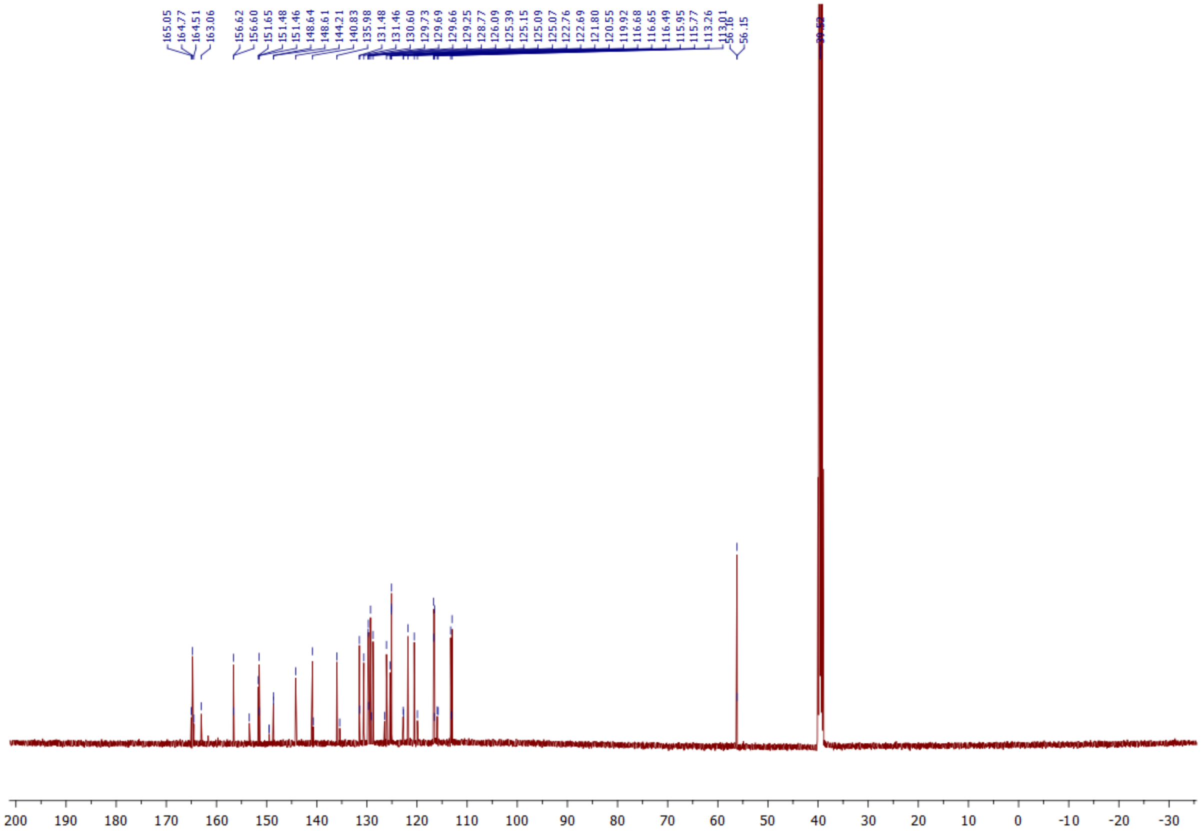
165.05
164.77
164.51
163.06
156.62
156.60
151.65
151.48
151.46
148.64
148.61
144.21
140.83
135.98
131.48
131.46
130.60
129.73
129.69
129.66
129.25
128.77
126.09
125.39
125.15
125.09
125.07
122.76
122.69
121.80
120.55
119.92
116.68
116.65
116.49
115.95
115.77
113.26
113.01
56.16
56.15
200
190
180
170
160
150
140
130
120
110
100
90
80
70
60
50
40
30
20
10
0
-10
-20
-30

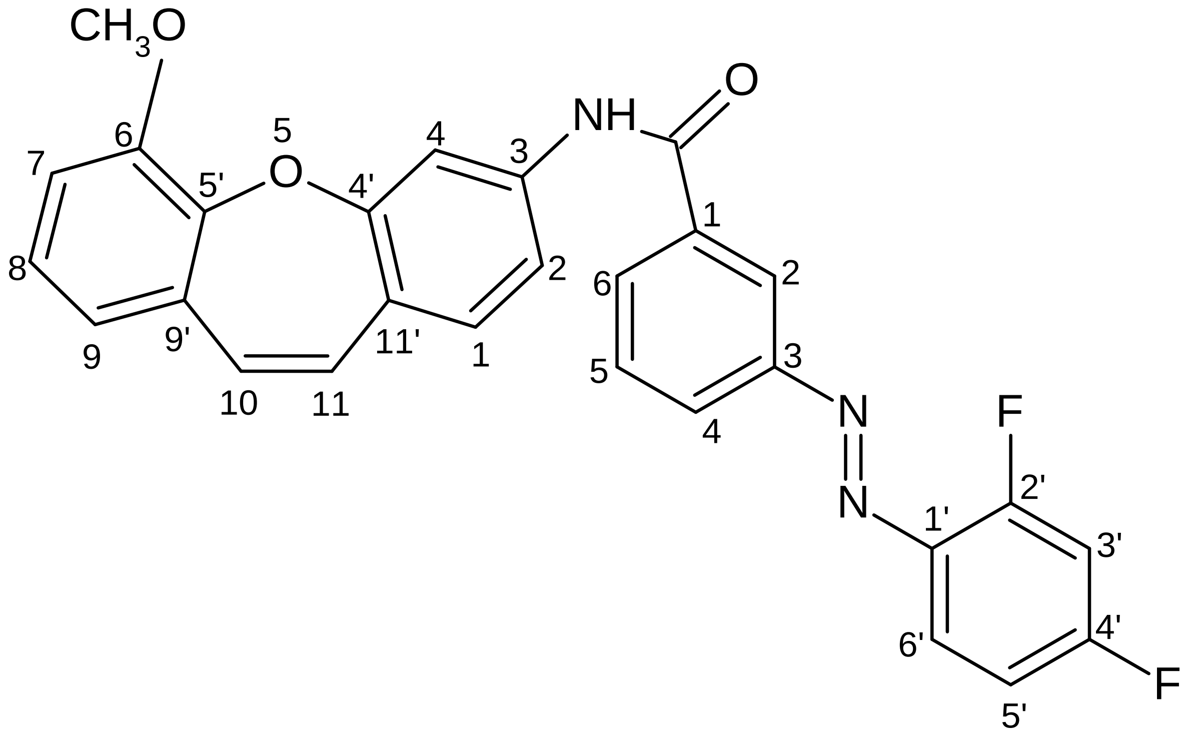
CH3O
O
NH
O
N
N
F
F
5
6
7
8
9
9'
10
11
11'
5'
4'
4
3
2
1
1
2
3
4
5
6
1'
2'
3'
4'
5'
6'

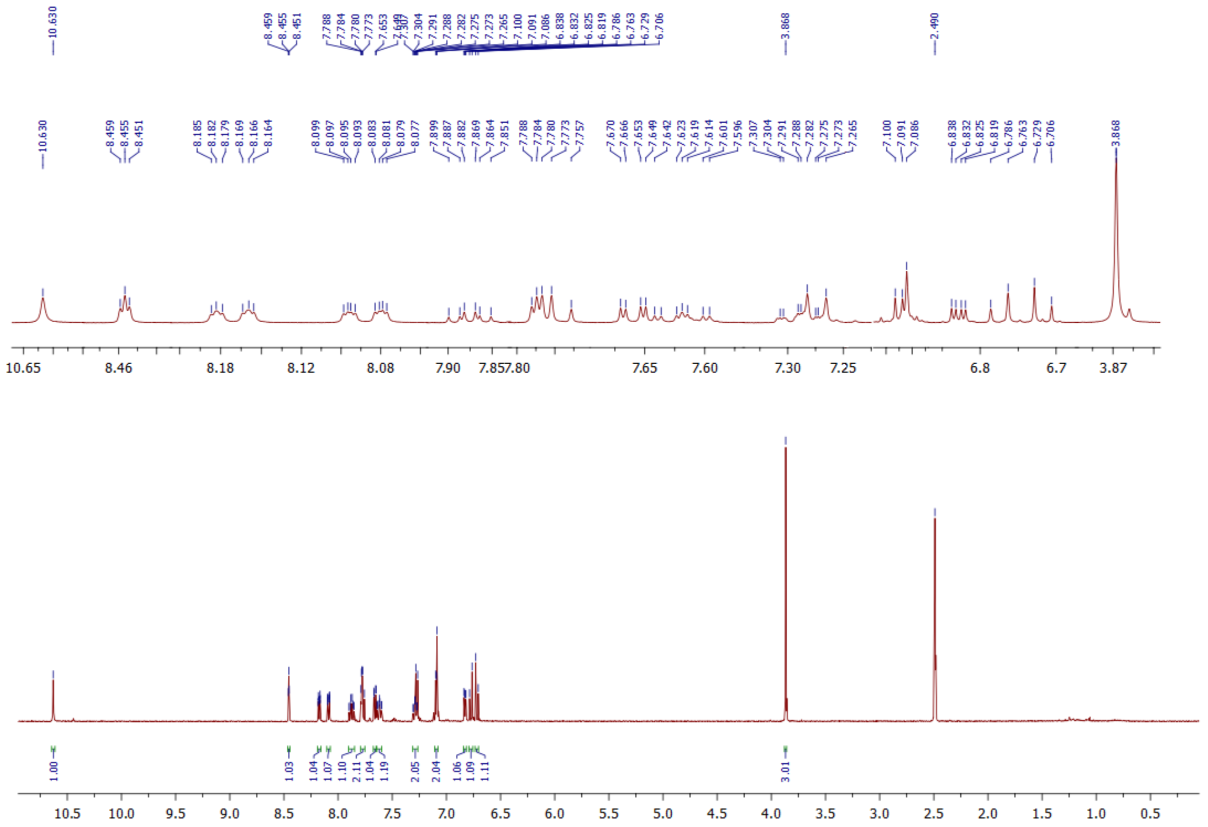

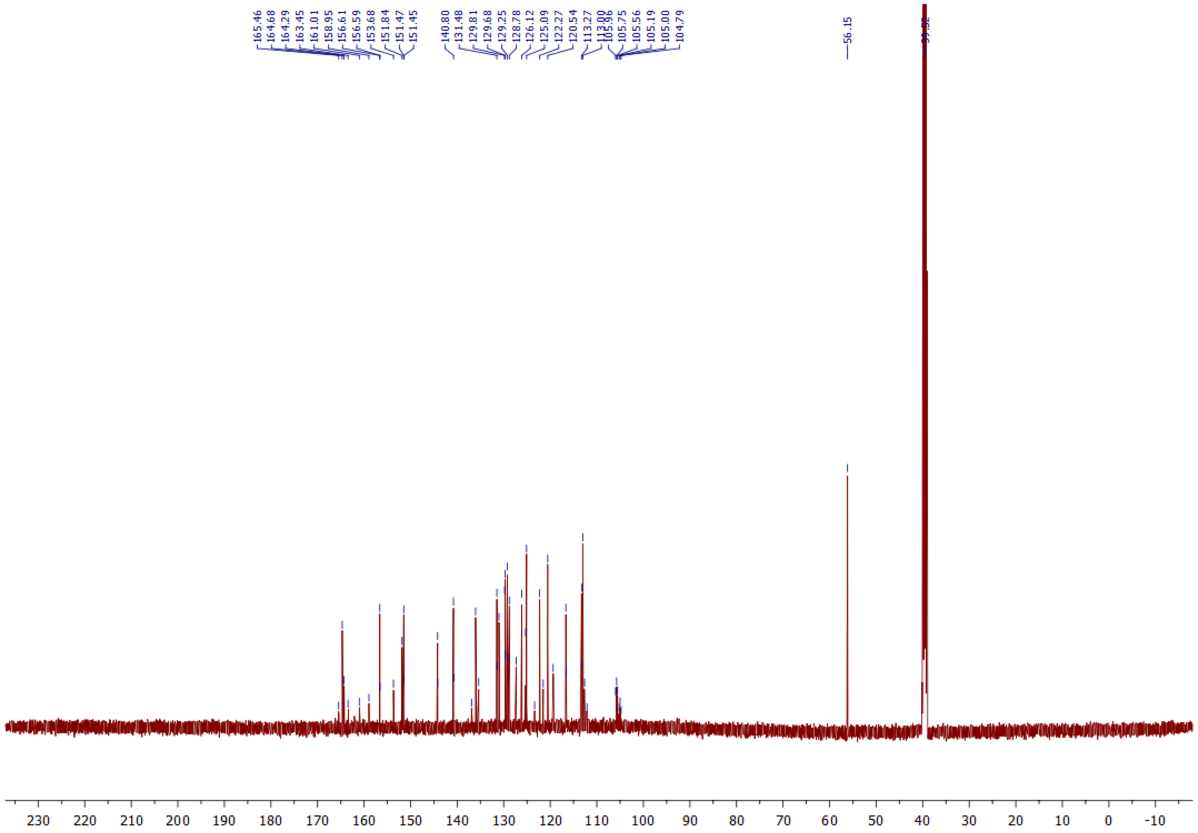
165.46
164.68
164.29
163.45
161.01
158.95
156.61
156.59
153.68
151.84
151.47
151.45
140.80
131.48
129.81
129.68
129.25
128.78
126.12
125.09
122.27
120.54
113.27
113.00
105.96
105.75
105.56
105.19
105.00
104.79
56.15
39.52
230
220
210
200
190
180
170
160
150
140
130
120
110
100
90
80
70
60
50
40
30
20
10
0
-10

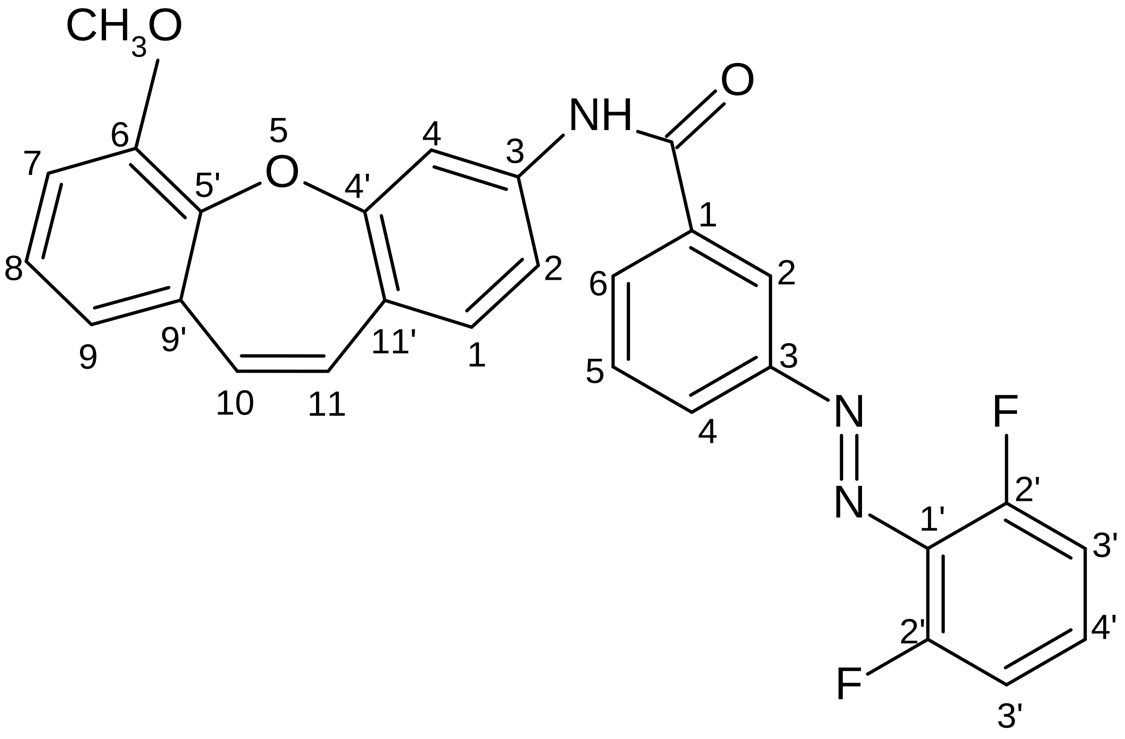
CH3O
5
O
NH
O
F
N
N
F

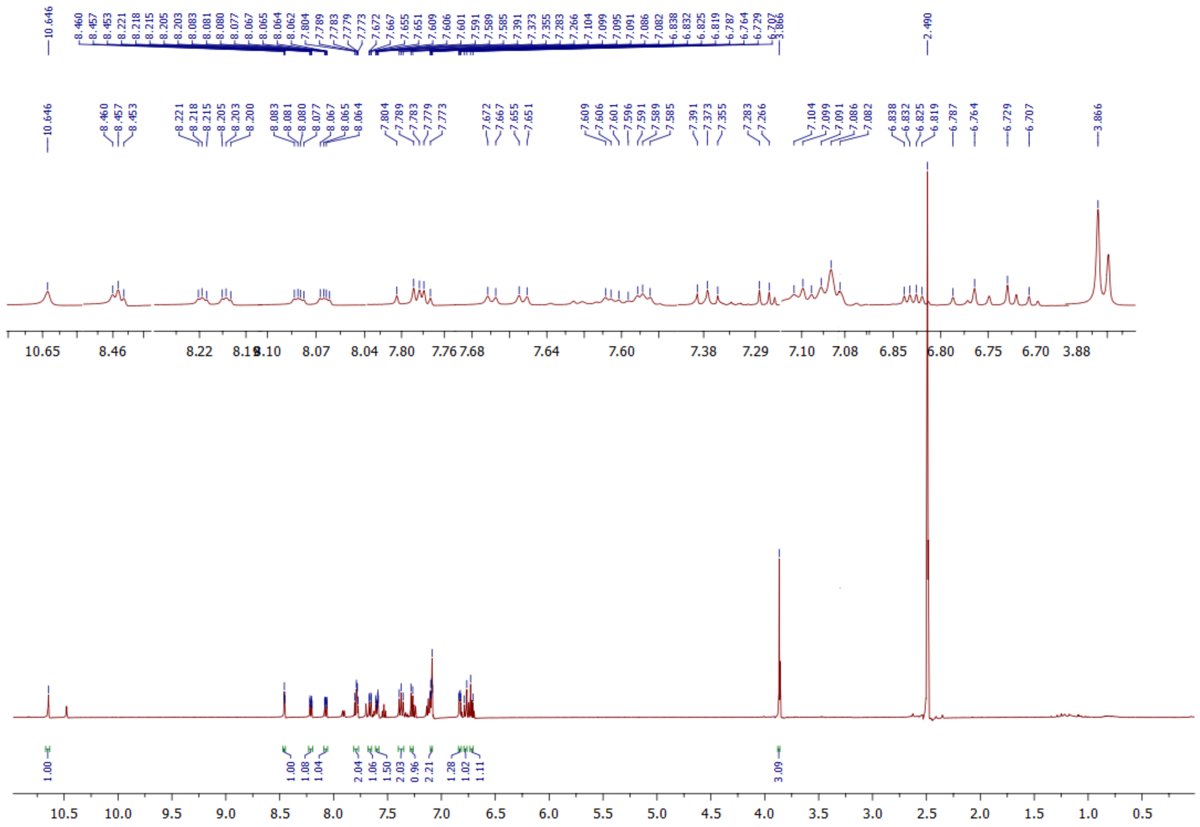

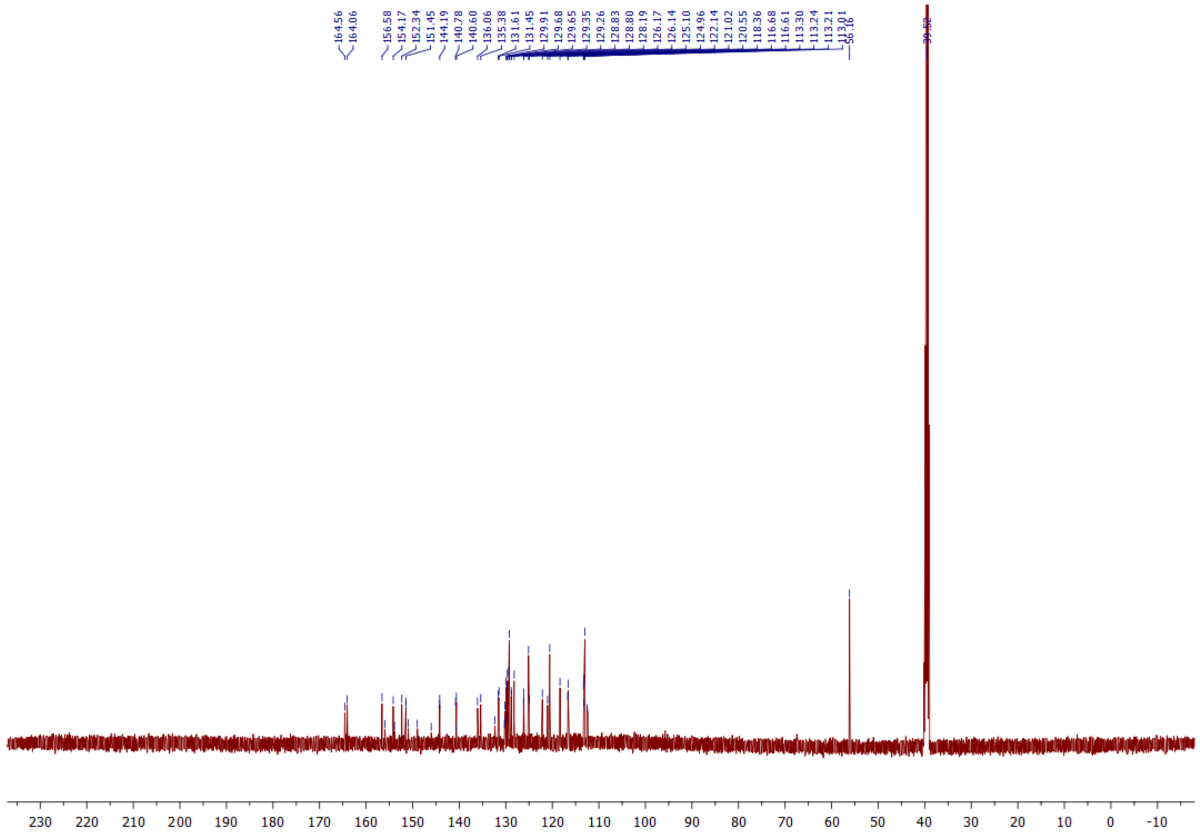
164.56
164.06
156.58
154.17
152.34
151.45
144.19
140.78
140.60
136.06
135.38
131.61
131.45
129.91
129.68
129.65
129.35
129.26
128.83
128.80
128.19
126.17
126.14
125.10
124.96
122.14
121.02
120.55
118.36
116.68
116.61
113.30
113.24
113.21
113.01
56.16
39.52
230 220 210 200 190 180 170 160 150 140 130 120 110 100 90 80 70 60 50 40 30 20 10 0 -10

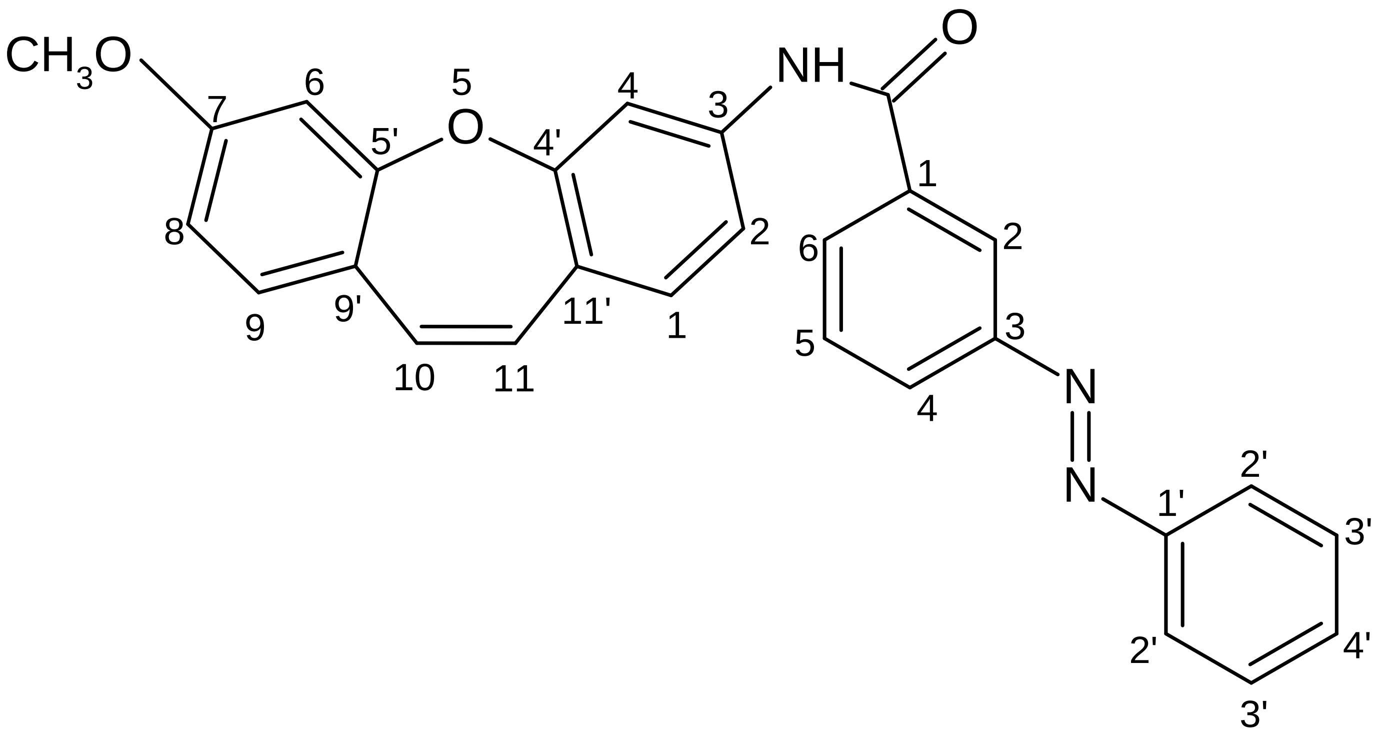

CH3O
6
7
5
O
4
3
NH
O
5'
4'
1
2
8
6
2
9'
11'
1
9
3
5
10
11
4
N
N
2'
1'
3'
2'
4'
3'

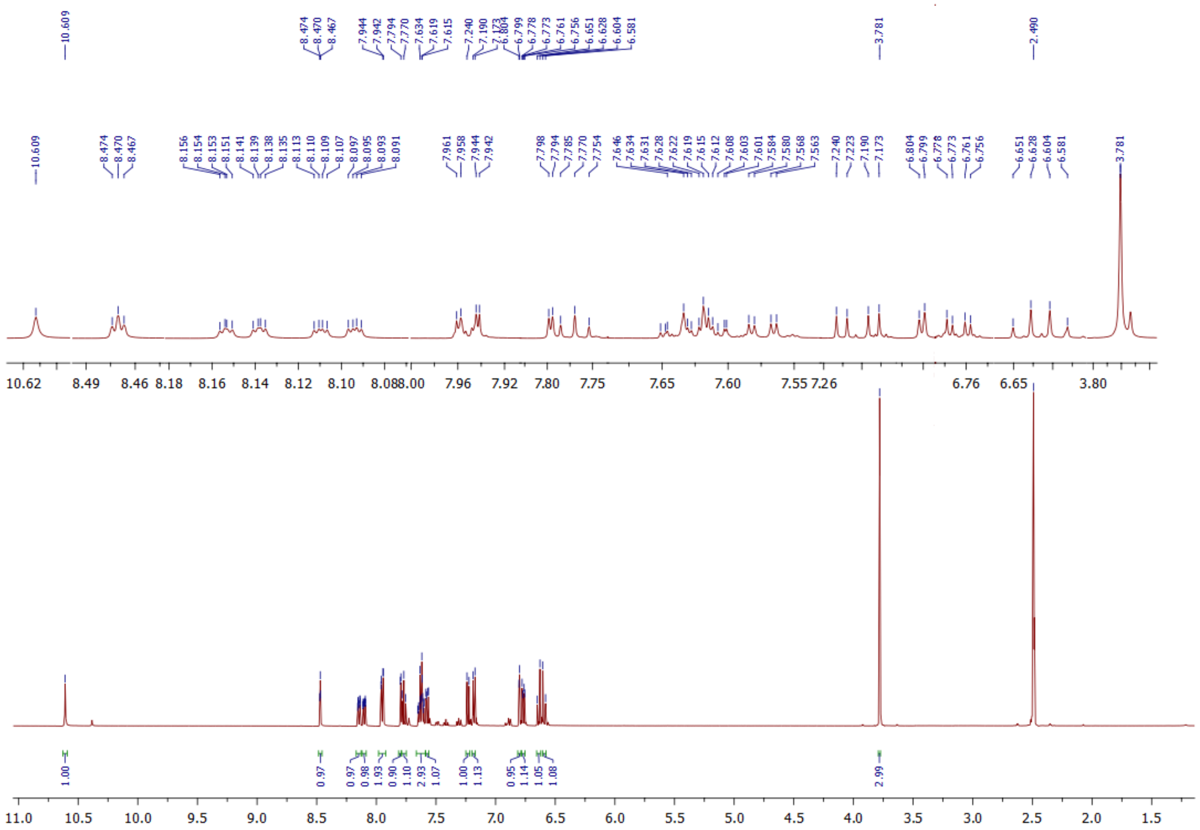

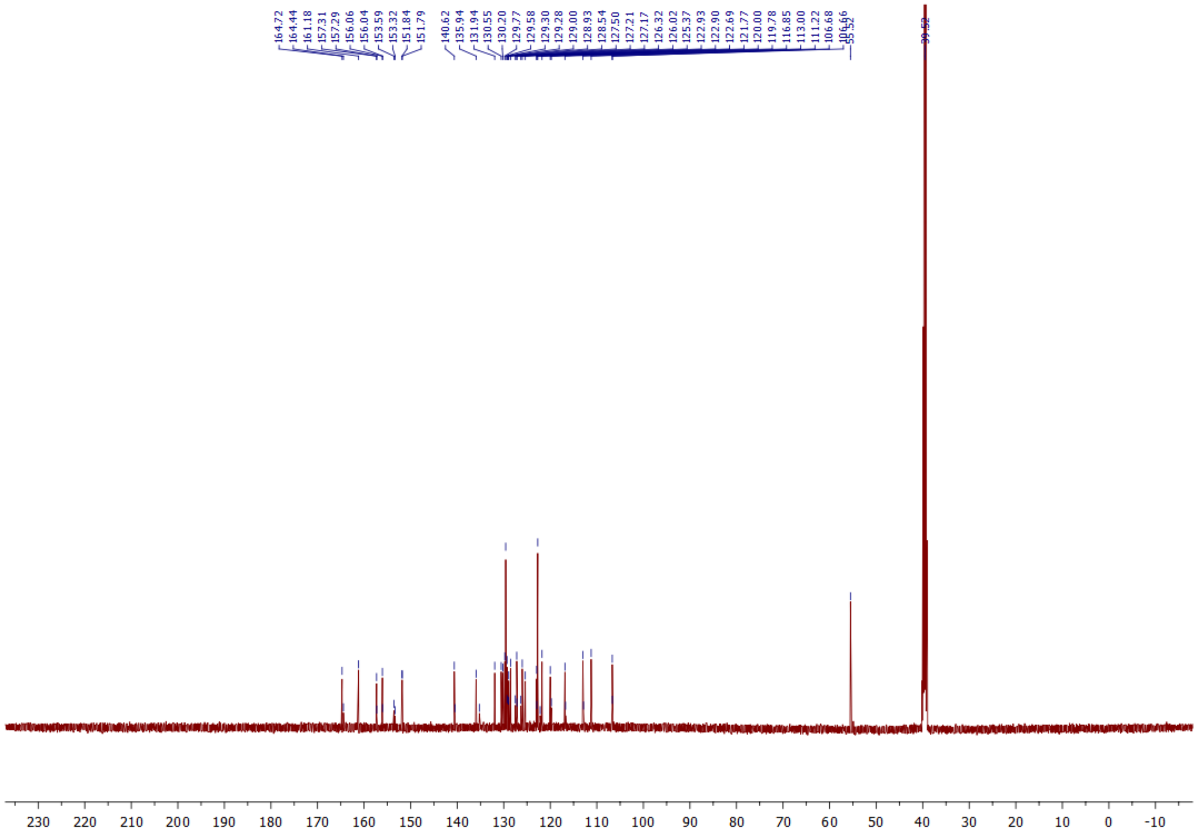

164.72
164.44
161.18
157.31
157.29
156.06
156.04
153.59
153.32
151.84
151.79
140.62
135.94
131.94
130.55
130.20
129.77
129.58
129.30
129.28
129.00
128.93
128.54
127.50
127.21
127.17
126.32
126.02
125.37
122.93
122.90
122.69
121.77
120.00
119.78
116.85
113.00
111.22
106.68
106.66
55.52
39.52
230 220 210 200 190 180 170 160 150 140 130 120 110 100 90 80 70 60 50 40 30 20 10 0 -10

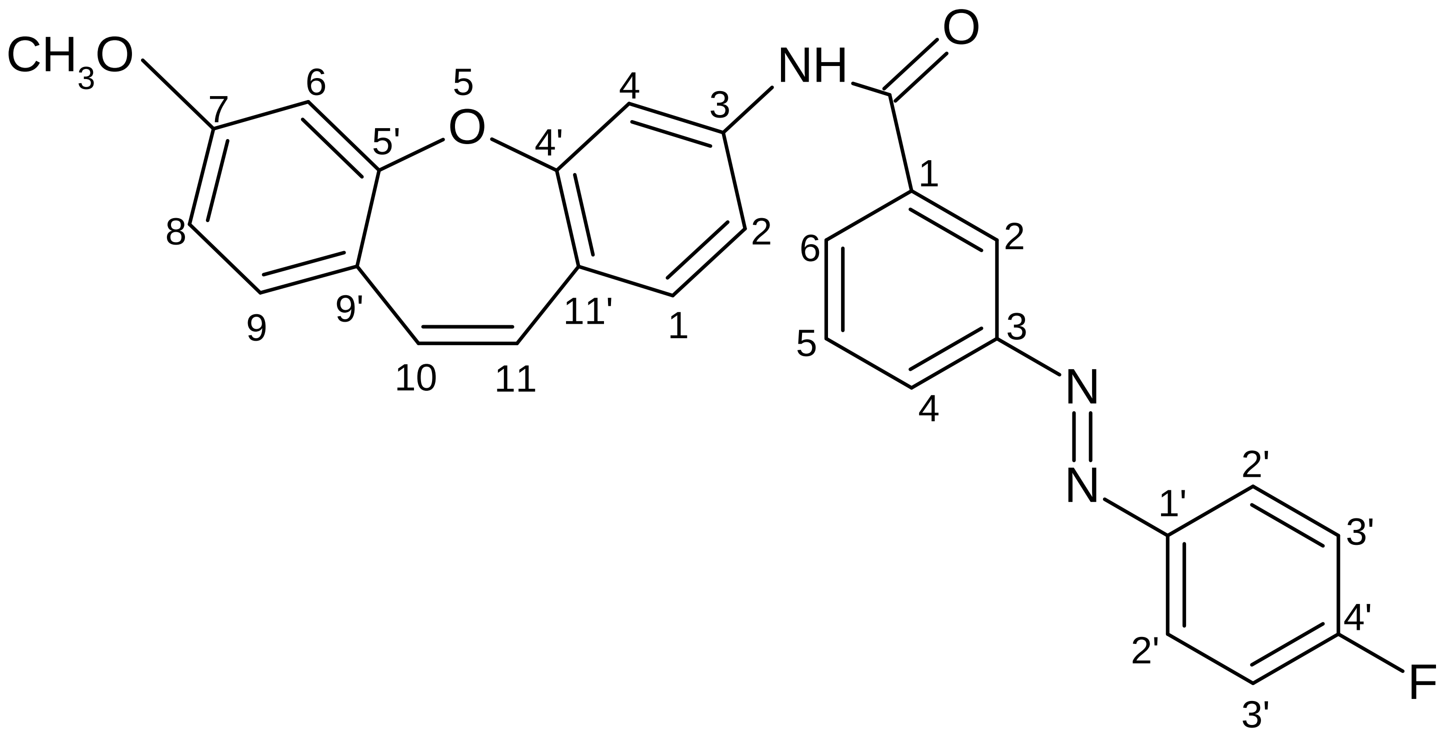
CH3O
7
6
5
O
5'
4'
4
3
NH
O
1
2
6
8
9
9'
10
11
11'
1
2
3
5
4
N
N
1'
2'
3'
4'
2'
3'
F

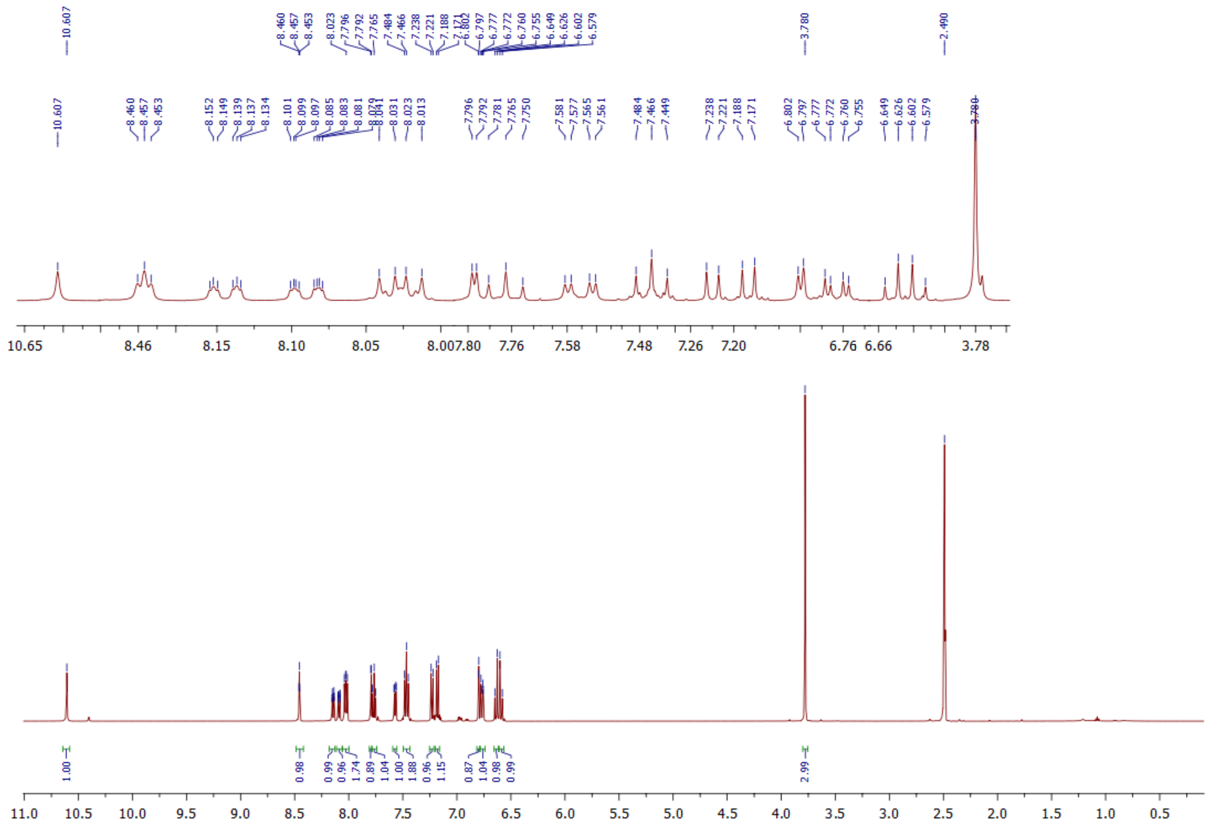

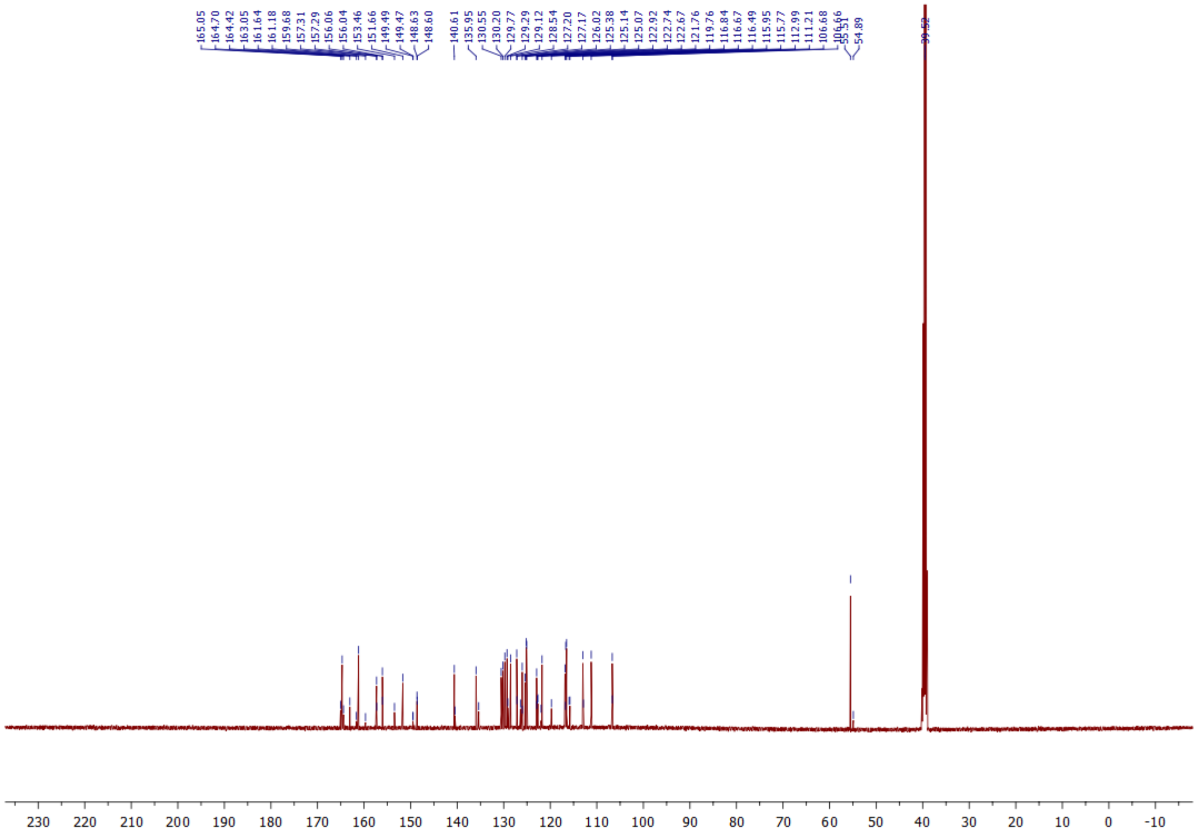
165.05
164.70
164.42
163.05
161.64
161.18
159.68
157.31
157.29
156.06
156.04
153.46
151.66
149.49
149.47
148.63
148.60
140.61
135.95
130.55
130.20
129.77
129.29
129.12
128.54
127.20
127.17
126.02
125.38
125.14
125.07
122.92
122.74
122.67
121.76
119.76
116.84
116.67
116.49
115.95
115.77
112.99
111.21
106.68
106.66
55.51
54.89
39.52
230
220
210
200
190
180
170
160
150
140
130
120
110
100
90
80
70
60
50
40
30
20
10
0
-10

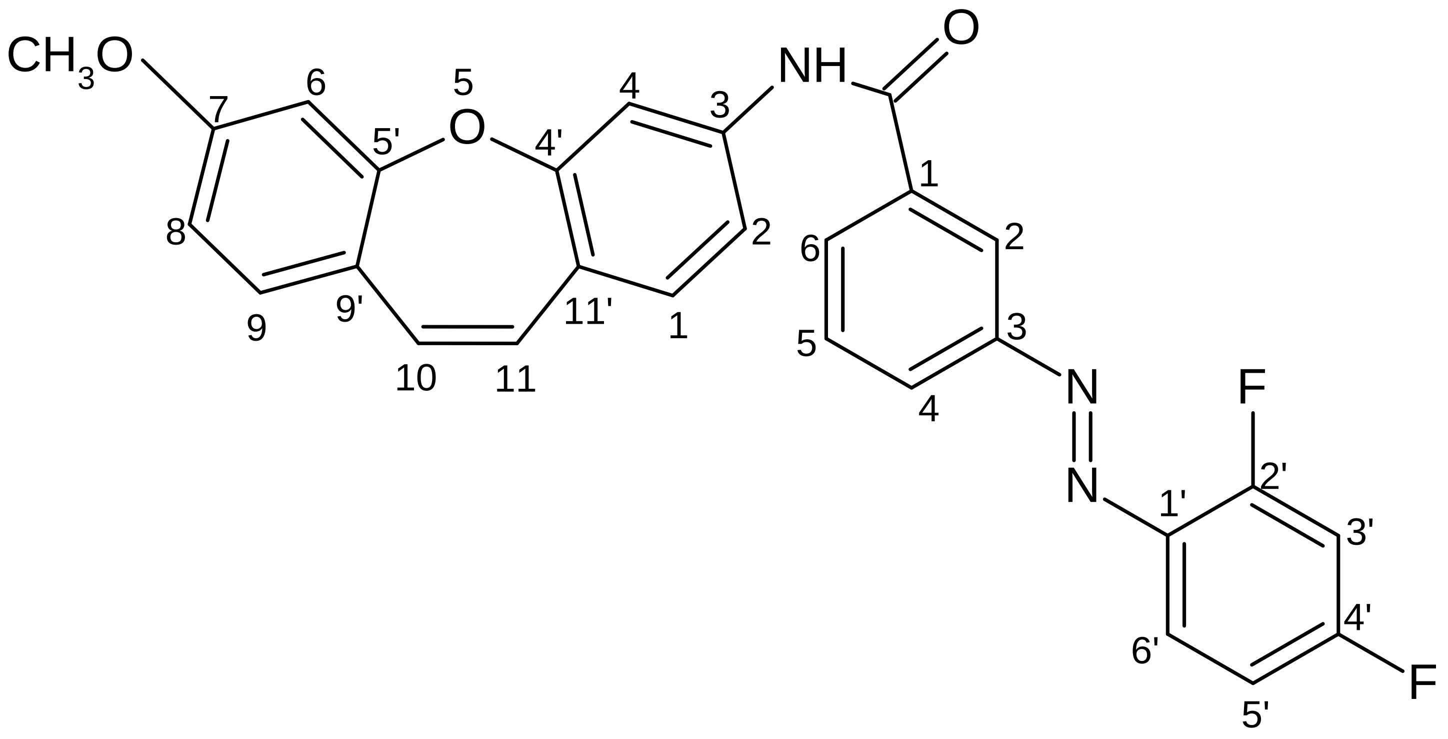

CH3O
O
NH
O
N
N
F
F
5
6
7
8
9
9'
10
11
11'
1
2
3
4
4'
5'
1
2
3
4
5
6
1'
2'
3'
4'
5'
6'

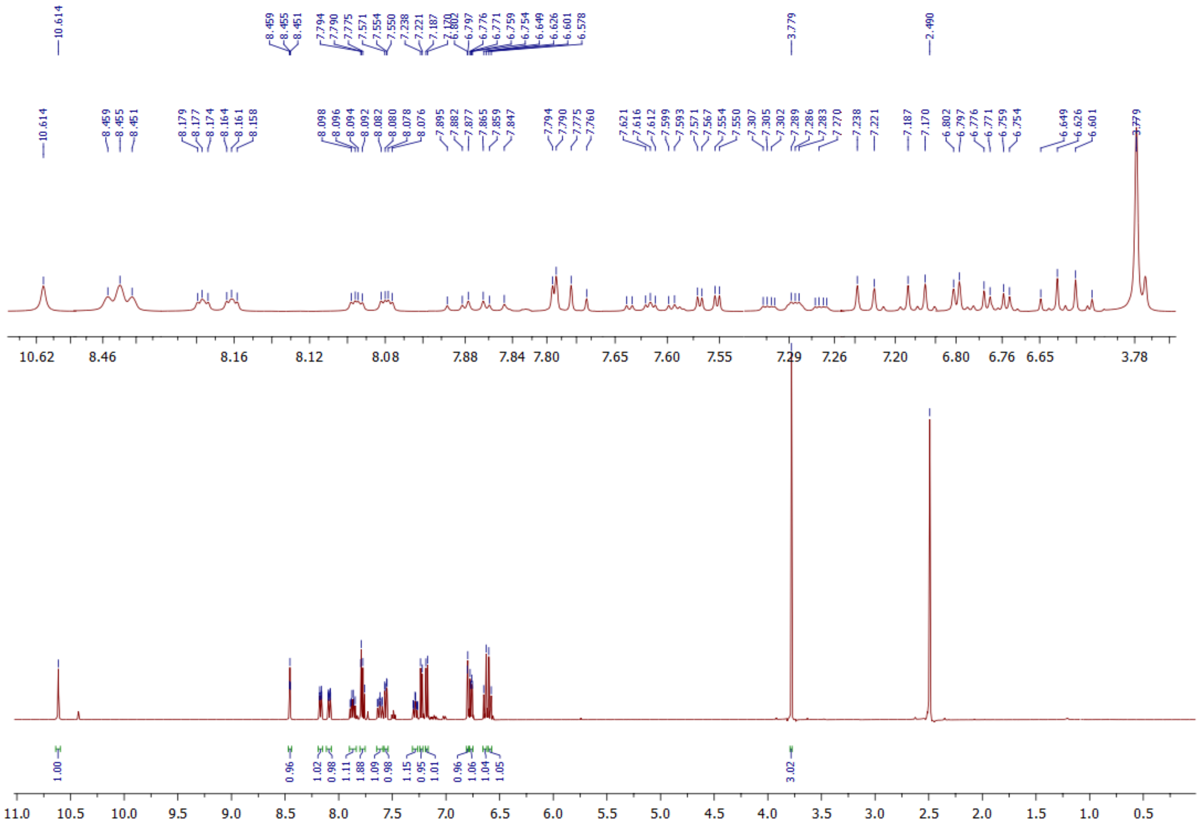

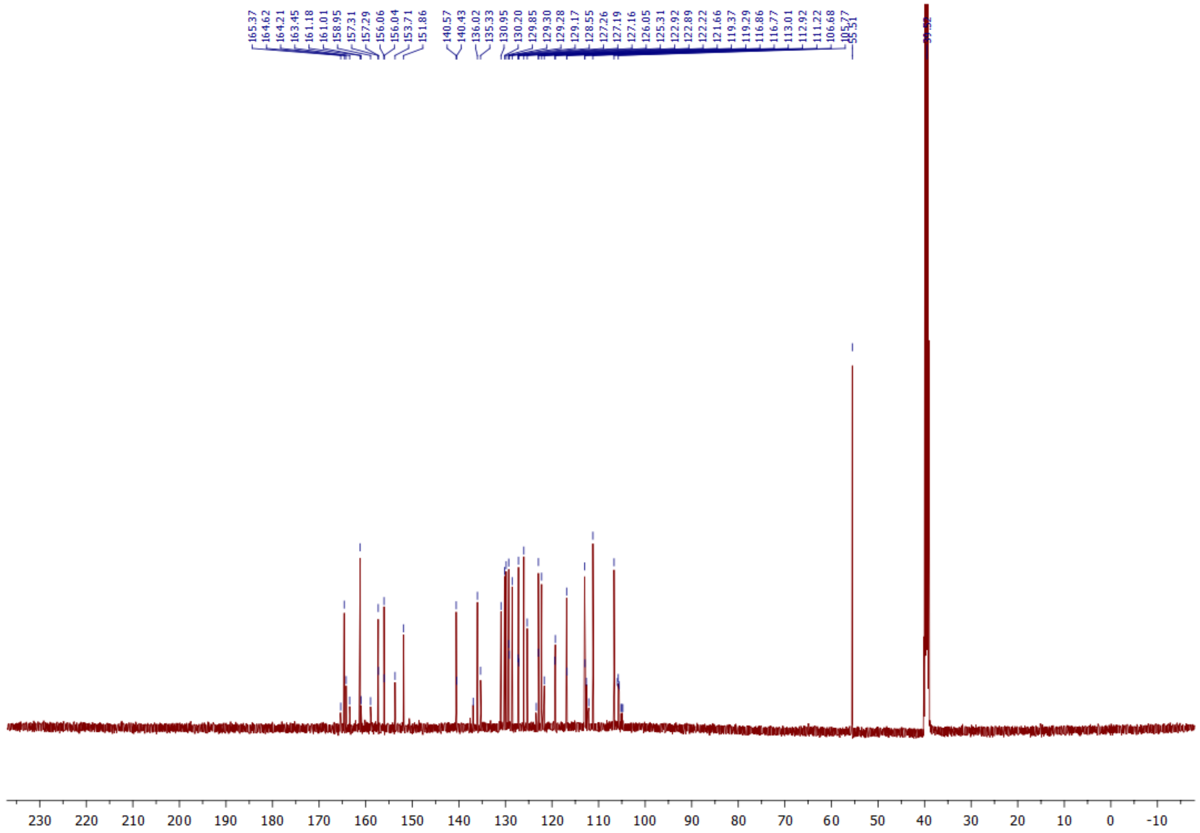

165.37
164.62
164.21
163.45
161.18
161.01
158.95
157.31
157.29
156.06
156.04
153.71
151.86
140.57
140.43
136.02
135.33
130.95
130.20
129.85
129.30
129.28
129.17
128.55
127.26
127.19
127.16
126.05
125.31
122.92
122.89
122.22
121.66
119.37
119.29
116.86
116.77
113.01
112.92
111.22
106.68
105.77
55.51
39.52
230 220 210 200 190 180 170 160 150 140 130 120 110 100 90 80 70 60 50 40 30 20 10 0 -10

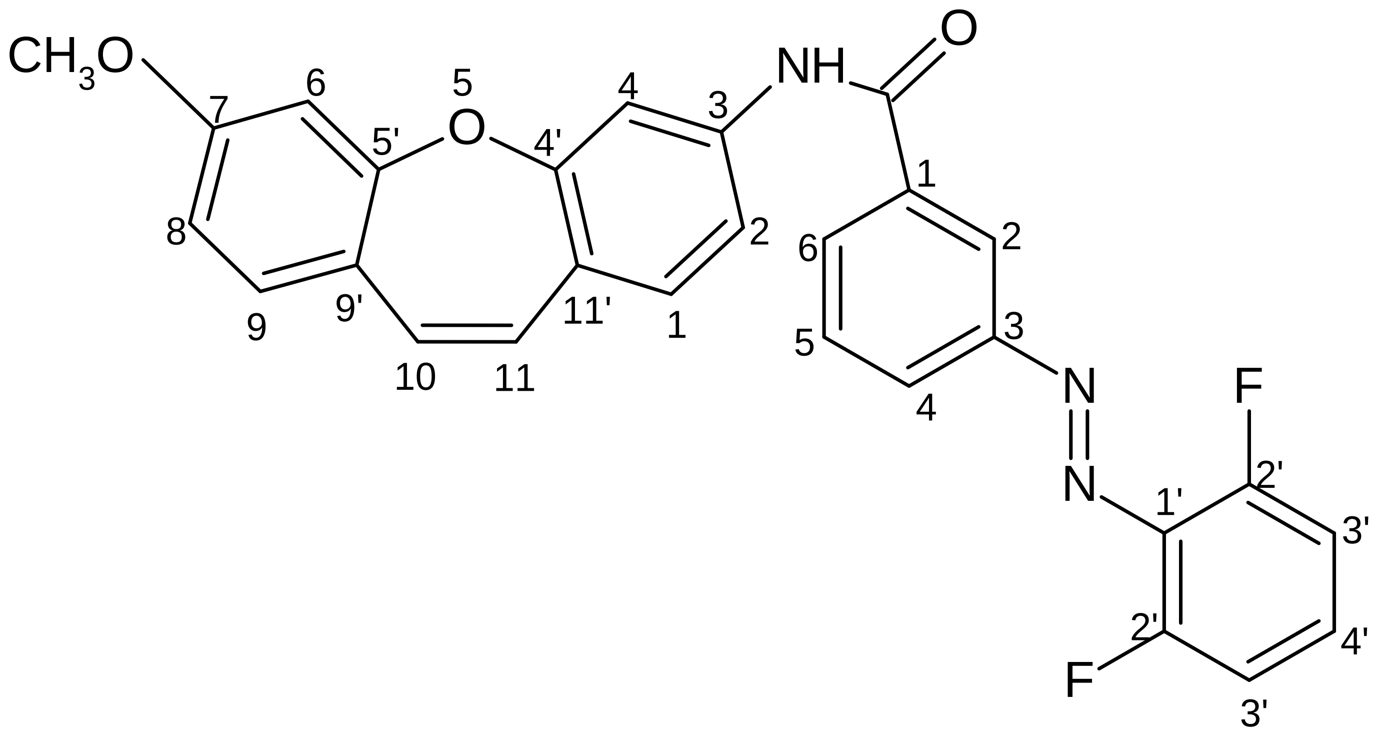

CH3O
NH
O
N
N
F
F

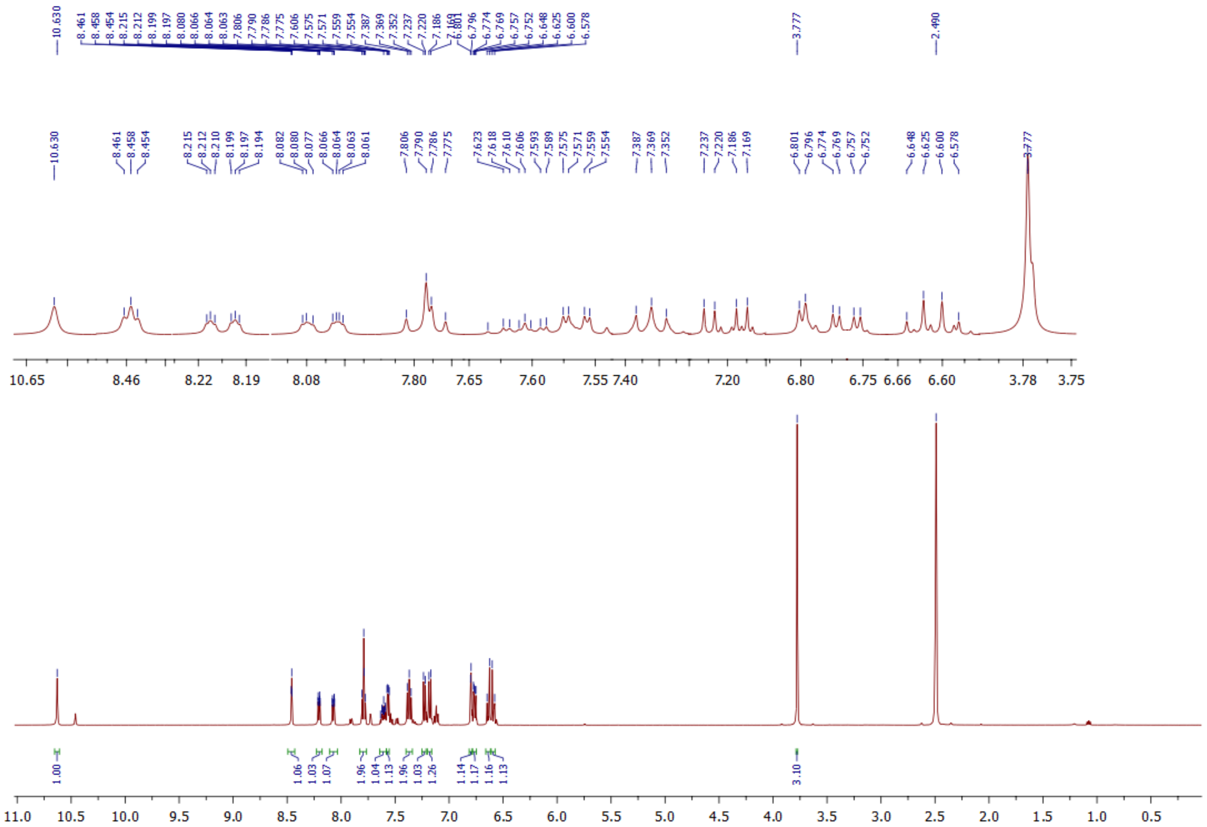

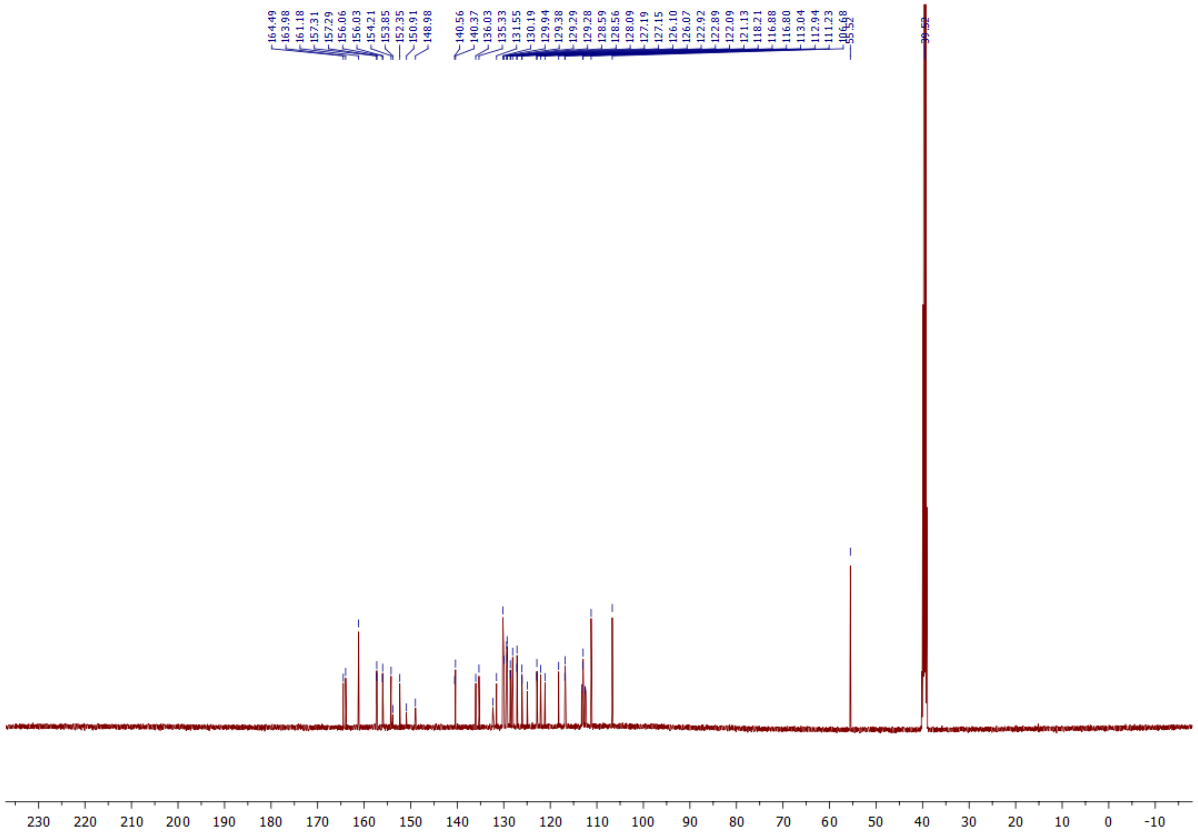
164.49
163.98
161.18
157.31
157.29
156.06
156.03
154.21
153.85
152.35
150.91
148.98
140.56
140.37
136.03
135.33
131.55
130.19
129.94
129.38
129.29
129.28
128.59
128.56
128.09
127.19
127.15
126.10
126.07
122.92
122.89
122.09
121.13
118.21
116.88
116.80
113.04
112.94
111.23
106.68
55.52
39.52
230
220
210
200
190
180
170
160
150
140
130
120
110
100
90
80
70
60
50
40
30
20
10
0
-10

4. Figure 2S. a) Scope of olefinic protons spectrum (DMSO-D_6) of (***5h E***) and (***5h Z***) reaction products measured at different temperatures; b) irradiation with light at wavelength λ= 525, 10 min.*

a) Temp. 80ºC

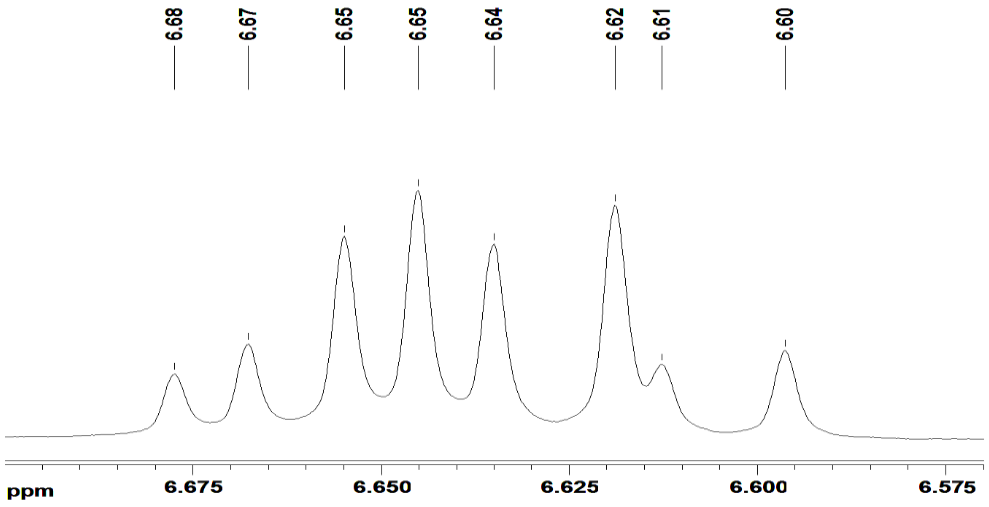

Temp. 100ºC

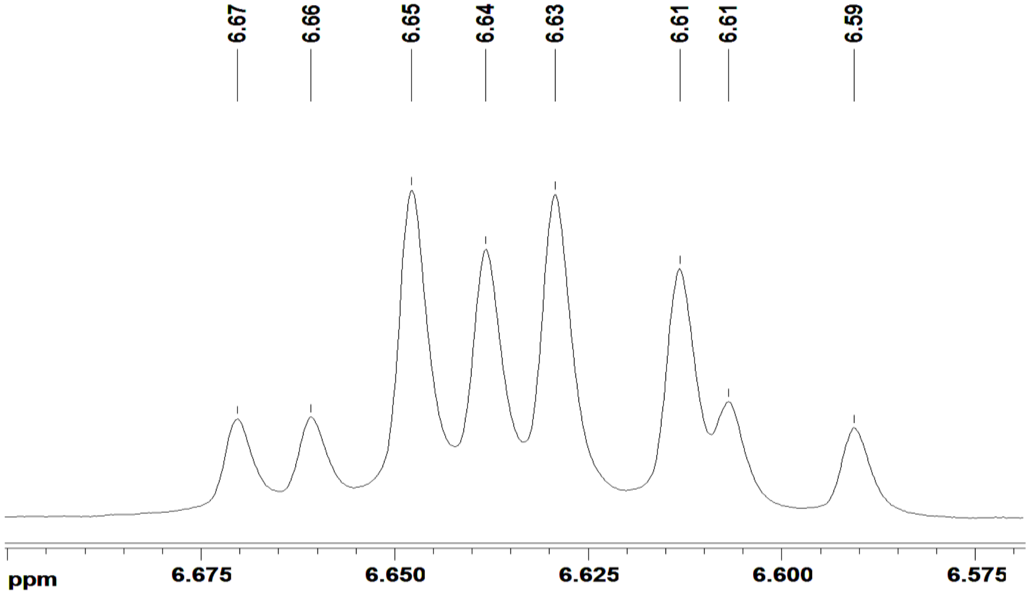

Temp. 110ºC

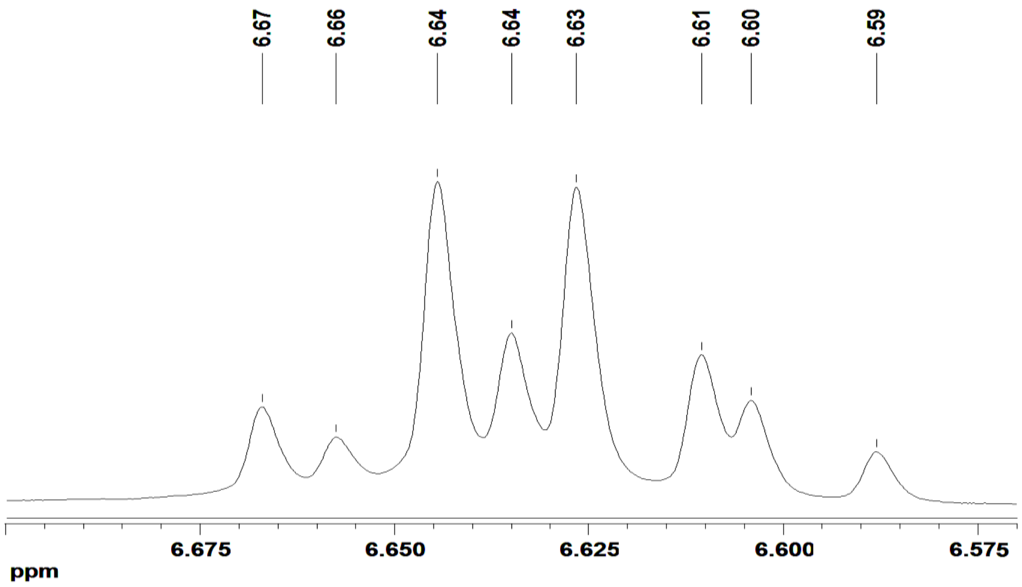

Temp. 150ºC

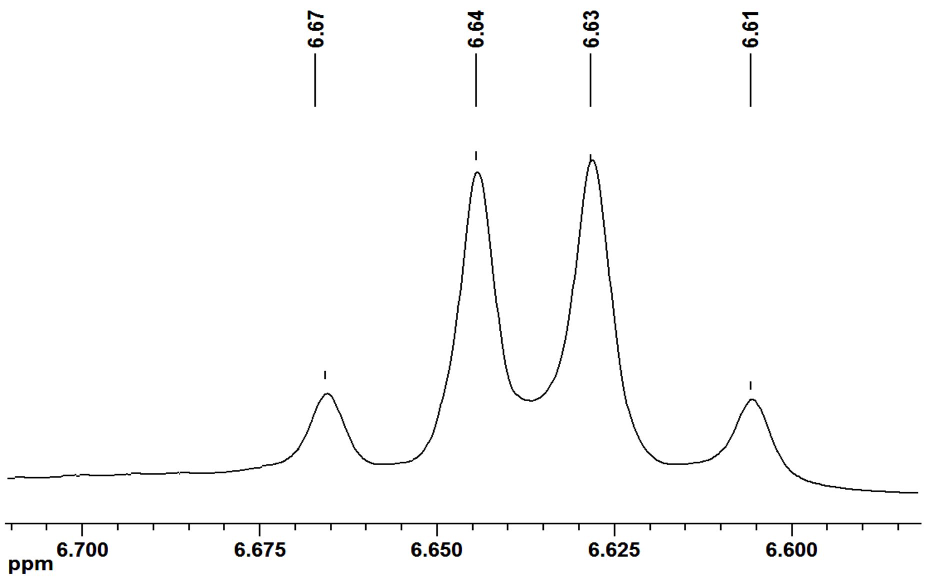

b) irradiation with light at wavelength λ= 525, 10 min (ratio *E/Z* 1:1)

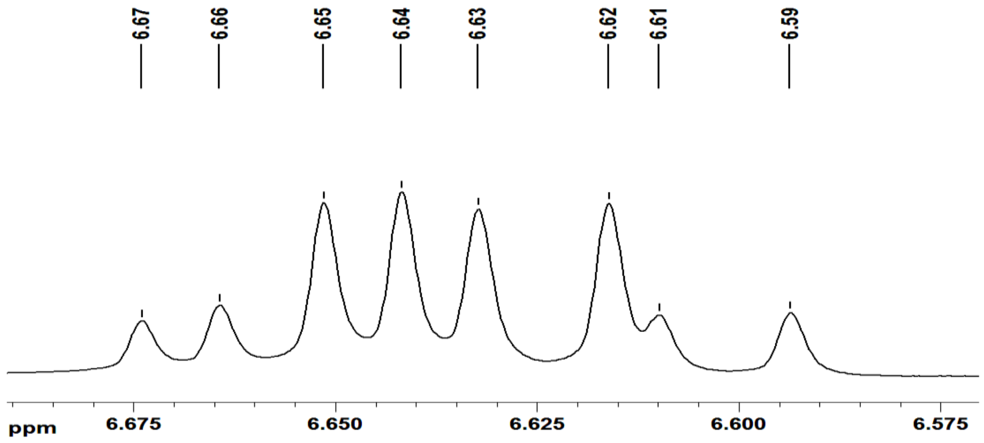

*NMR spectra after irradiation with light with a wavelength of λ = 525, after 10 min was made to determine the ***E*/*Z*** isomer ratio. We presented the spectral range of olefinic protons (DMSO-D_6) of the reaction products (**5h*E***)/(**5h*Z***). The ***E* / *Z*** isomer ratio for (**5d*E***)/(**5d*Z***) and (**5h*E***)/(**5h*Z***), after 10 min was 1 : 1.

5. References

[1] M.J. Frisch, G.W. Trucks, H.B. Schlegel, G.E. Scuseria, M.A. Robb, J.R. Cheeseman, J.A. Montgomery, T. Vreven, K.N. Kudin, J.C. Burant, J.M. Millam, S.S. Iyengar, J.Tomasi, V. Barone, B. Mennucci, M. Cossi, G. Scalmani, N. Rega, G.A. Petersson, H. Nakatsuji, M. Hada, M. Ehara, K. Toyota, R. Fukuda, J. Hasegawa, M. Ishida, T.Nakajima, Y. Honda, O. Kitao, H. Nakai, M. Klene, X. Li, J.E. Knox, H.P. Hratchian, J.B. Cross, V. Bakken, C. Adamo, J. Jaramillo, R. Gomperts, R.E. Stratmann, O. Yazyev, A.J. Austin, R. Cammi, C. Pomelli, J.W. Ochterski, P.Y. Ayala, K. Morokuma, G.A. Voth, P. Salvador, J.J. Dannenberg, V.G. Zakrzewski, S. Dapprich, A.D. Daniels, M.C. Strain, O. Farkas, D.K. Malick, A.D. Rabuck, K.Raghavachari, J.B. Foresman, J.V. Ortiz, Q. Cui, A.G. Baboul, S. Clifford, J. Cioslowski, B.B. Stefanov, G. Liu, A. Liashenko, P. Piskorz, I. Komaromi, R.L. Martin, D.J. Fox, T. Keith, M.A. Al-Laham, C.Y. Peng, A. Nanayakkara, M. Challacombe, P.M.W. Gill, B. Johnson, W. Chen, M.W. Wong, C. Gonzalez, J.A. Pople, Gaussian Inc., Wallingford, CT, **2004** (Gaussian 03, Revision E.01).

[2] J. Tomasi, B. Mennucci, R. Cammi, Quantum mechanical continuum solvation models, *Chem. Rev*. **2005**, *105*, 2999-3094.

[3] RCSB Protein Data Bank—RCSB PDB. Available online: http://www.rcsb.org/pdb/home/home.do (accessed on 23 February **2004**).

[4] O. Trott, A.J. Olson, AutoDock Vina: Improving the speed and accuracy of docking with a new scoring function, efficient optimization and multithreading. *J. Comput. Chem*. **2010**, *31*, 455–461.

[5] E.F. Pettersen, T.D. Goddard, C.C. Huang, G.S. Couch, D.M. Greenblatt, E.C. Meng, T.E. Ferrin, UCSF Chimera-a visualization system for exploratory research and analysis. *J. Comput. Chem*. **2004**, *25*, 1605–1612.

[6] The PyMOL Molecular Graphics System, Version 1.3, Schrödinger LLC, 2010.

[7] S. Salentin, S. Schreiber, V.J. Haupt, M.F. Adasme, M. Schroeder, PLIP: fully automated protein-ligand interaction profiler, Nucleic Acids Res. 43 (2015) W443–W447.
